# Design and synthesis of novel 3-triazolyl-1-thiogalactosides as galectin-1, -3 and -8 inhibitors†

**DOI:** 10.1039/d2ra03163a

**Published:** 2022-06-30

**Authors:** Sjors van Klaveren, Jaka Dernovšek, Žiga Jakopin, Marko Anderluh, Hakon Leffler, Ulf J. Nilsson, Tihomir Tomašič

**Affiliations:** University of Ljubljana, Faculty of Pharmacy, Department of Pharmaceutical Chemistry Aškerčeva cesta 7 1000 Ljubljana Slovenia tihomir.tomasic@ffa.uni-lj.si; Centre for Analysis and Synthesis, Department of Chemistry, Lund University SE-221 00 Lund Sweden ulf.nilsson@chem.lu.se; Department of Laboratory Medicine, Section MIG, Lund University BMC-C1228b Klinikgatan 28 221 84 Lund Sweden

## Abstract

Galectins are galactoside-binding proteins that play a role in various pathophysiological conditions, making them attractive targets in drug discovery. We have designed and synthesised a focused library of aromatic 3-triazolyl-1-thiogalactosides targeting their core site for binding of galactose and a subsite on its non-reducing side. Evaluation of their binding affinities for galectin-1, -3, and -8N identified acetamide-based compound 36 as a suitable compound for further affinity enhancement by adding groups at the reducing side of the galactose. Synthesis of its dichlorothiophenyl analogue 59 and the thiodigalactoside analogue 62 yielded promising pan-galectin inhibitors.

## Abstract 2

A new series of potent galectin ligands based on the galactose and triazole moieties was designed and synthesised.

## Graphical abstract

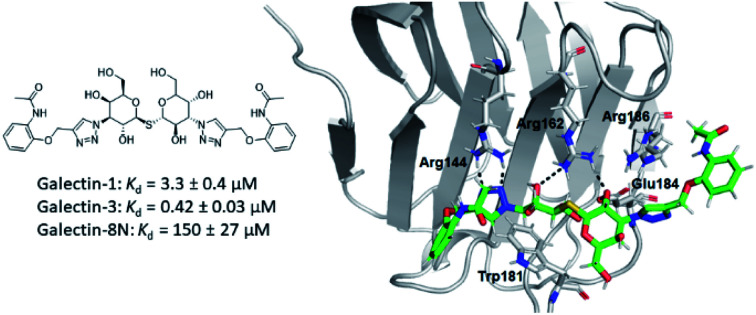

## 1 Introduction

Galectins are lectins that specifically bind β-galactoside-containing glycoconjugates in their carbohydrate recognition domain (CRD).^1^ The mode of action of galectins is thought to involve protein–protein interactions and binding to glycoconjugates both on the cell surface and in the extracellular matrix.^2,3^ Galectins are involved in a variety of processes, including cell differentiation, immune regulation, angiogenesis, and pathogen recognition.^4,5^ These properties also link galectins to a number of pathologies, including the immune response and cancer biology. As these processes are being studied, it is becoming increasingly clear that blocking the CRD of various galectins may have a therapeutic effect.^3^ Nevertheless, galectins and their interactions are still an intensely studied topic, as their purpose and potential are not fully understood. Moreover, many galectins appear to have overlapping or opposing functions.^6–8^

Galectin-1 is one of the members of the galectin family found in humans. It is active both intra- and extracellularly, regulating T-cell behaviour and fate, stimulating inflammation, and is involved in host–pathogen interactions.^9^ Upregulation of galectin-1 in and around tumour cells has been linked to malignant tumour progression, tumour immune escape, and metastasis.^10,11^

Galectin-3 is the only oligomeric-type galectin, making it an excellent protein for cross-linking cell surface glycans and extracellular interaction partners for recognition or immobilisation.^2^ In the lung, small molecules targeting galectin-3 are effective in treating pulmonary fibrosis.^12^ The galectin family is also involved in binding infectious viruses, and galectin-1, -3, and -8 each have a distinct function in HIV-1 infection.^13,14^ Of note, COVID-19 infection-induced hyperinflammation has been linked to galectin-3,^15^ and galectin-8 has been shown to affect viral recognition on the cell surface for SARS-CoV-2.^16^

Galectin-8 has been found to be involved in tumour development and metastasis, in part due to its stimulatory effect on angiogenesis and lymphangiogenesis.^17^ This function also makes galectin-8 an important player in graft rejection, where it interacts with endothelial growth factors.^18^ Galectin-8 also has a broader function in inflammation for the immune response,^19^ and is involved in the neuroprotective environment in the brain.^20^

**Fig. 1:**
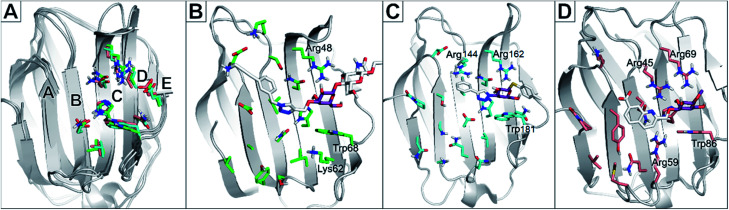
(A): Alignment of galectin CRD crystal structures indicating binding subsites A–E. Residues are shown as green (galectin-1, PDB code: 5MWX), cyan (galectin-3, PDB code: 6QLN), and salmon (galectin-8N, PDB code: 7AEN) coloured sticks. (B): Crystal structure of galectin-1 in complex with ligand JB60 (PDB code 5MWK). (C): Crystal structure of galectin-3 in complex with a fluoroaryltriazole disaccharide (PDB code 6QLN). (D): Crystal structure of galectin-8N in complex with a quinolinyl galactopyranoside (PDB code 7AEN). In all structures, the ligand is shown in sticks with white carbons, with the galactose moiety shown in purple to emphasise the predominant binding of the sugar moiety in subsite C. This galactose moiety of the ligands binds between a Trp68 and Arg48 in galectin-1; Trp181 and Arg162 in galectin-3; and Trp86 and Arg69 in galectin-8N.

Albeit galectin-1 is expressed as a monomer, it is known to homo-dimerise to functionally present two CRDs per unit. Galectin-8 is a tandem repeat-type galectin expressed with two distinct CRDs, designated galectin-8C and -8N for the C- and N-terminal domains, respectively.^21^ The structure of the CRDs is relatively conserved among galectins and always binds d-galactose moieties in the same manner (Fig. 1). In the nomenclature used to compare and describe galectin CRDs, the d-galactose binds in the subsite C. This conserved binding site makes the galactose unit a central core to which other moieties are attached and directed into the subsites AB or DE (Fig. 1A) on either side of the galactose-binding subsite C.^22^

**Fig. 2:**
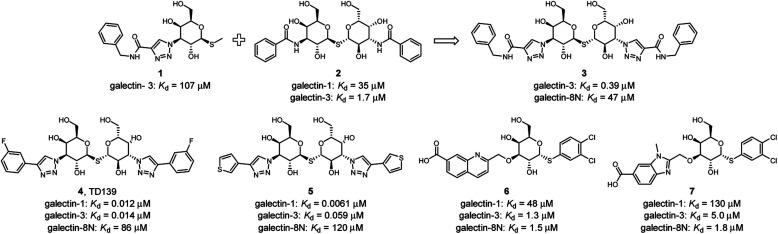
Selected known galectin inhibitors with triazolyl groups or related functionalities.

Several ligands of galectin-1, -3 and -8 are known (Fig. 2). A common feature of many of these structures is an electron-rich aromatic moiety attached to galactose C3 to occupy the subsite B of the galectin CRDs. This subsite often contains cationic amino acids (Fig. 1): lysine in galectin-1, lysine and arginine in galectin-3, and two arginine residues in galectin-8N. For several types of ligands for these galectins, subsite B is targeted with great success with a triazole ring (Fig. 2). 3-Triazolyl-1-thiogalactosides such as triazole 1 were first described as ligands for galectin-3.^23^ Combined with the contemporary discovery of symmetrical thiodigalactosides such as 2,^24^ and their subsequent optimisation,^25^ these results led to the class of 1,2,3-triazolyl thiodigalactosides, including 3.^26^

Optimisation of this class led to the discovery of TD139 (4, Fig. 2) with nanomolar affinity,^12^ which is currently in advanced clinical trials.^27^ The same study also described 3-thienyl-triazolyl thiodigalactoside 5, which is selective and has high affinity for galectin-1.^28^ Other high-affinity binders designed to inhibit galectins include the quinoline derivative 6,^29^ which contains an electron-rich nitrogen atom bound to subsite B of galectins, which most likely forms an ion–dipole interaction with Arg45 in galectin-8N (Fig. 1D). This interaction feature was extended in benzimidazole 7, which was designed to optimise the selectivity for galectin-8N.^30^ Many other galectin inhibitors were recently reported.^31–34^

To explore chemical space in the subsites A and B and to investigate possible selectivity between galectin-1, -3, and -8, we assembled a collection of differently substituted phenols that can be attached *via* a triazole ring to C3 of galactose. The phenols were varied with respect to both the chemical nature of the substituents and the substitution pattern to efficiently explore the subsites A and B. The synthesised compounds were evaluated for binding to galectin-1, -3 and -8N, and their binding interactions were studied by docking and molecular dynamics (MD) simulations.

## 2 Results and discussion

### 2.1 Design and synthesis

Tolyl 1-thio-β-d-galactopyranosides bearing C3-bound triazoles were designed for the synthesis of compounds targeting subsite B (Table 1). The azide group of 8 (ref. 35 and 36) provided an efficient starting point for diversification by copper(i)-catalysed azide–alkyne Huisgen cycloaddition (CuAAC), and the various alkyne counterparts 9–32 (Table 1) were prepared by a facile synthesis using in-house available phenols. The resulting 3-triazolyl-1-thiogalactosides 33–56 have a flexible phenoxymethyl attached to the triazole, which was hypothesised to form productive interactions in subsite AB and was used to identify distinguishable interactions between galectin-1, -3, and -8N that may lead to selective inhibition of these three galectins.

**Table 1:** 3-Triazolyl-1-thiogalactosides 33–56 synthesised from 3-azido-galactopyranoside 8 and phenoxypropargyl intermediates 9–32 using CuAAC chemistry

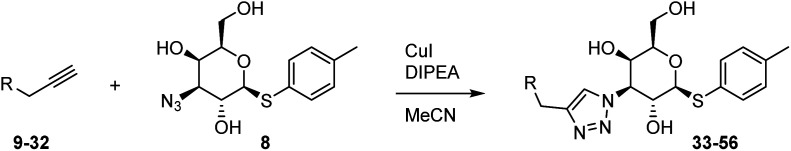							
Compound	R	Compound	R	Compound	R	Compound	R
33	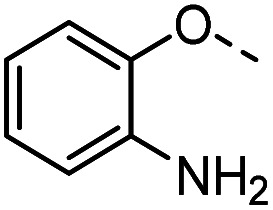	39	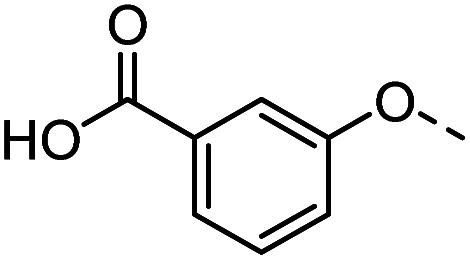	45	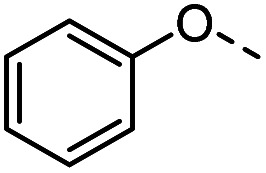	51	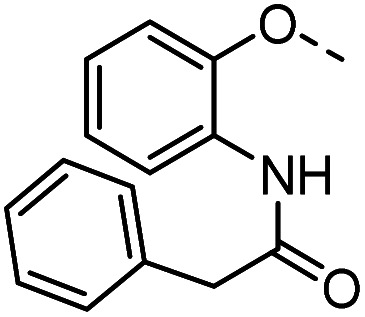
34	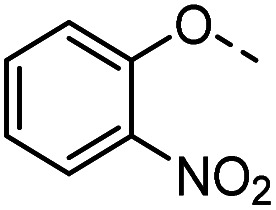	40	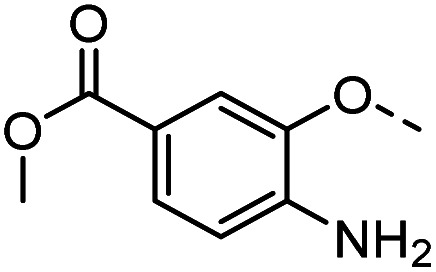	46	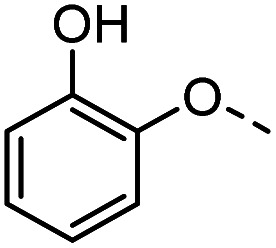	52	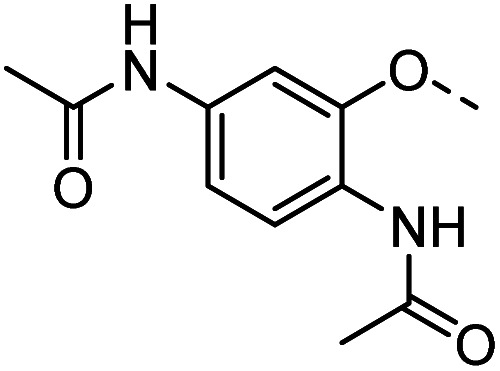
35	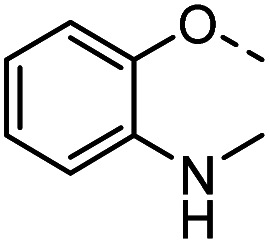	41	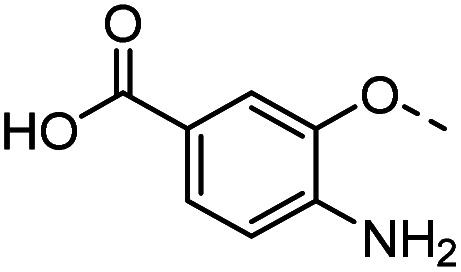	47	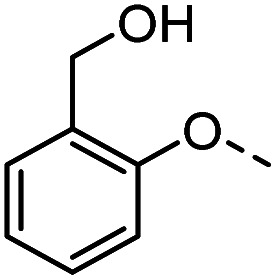	53	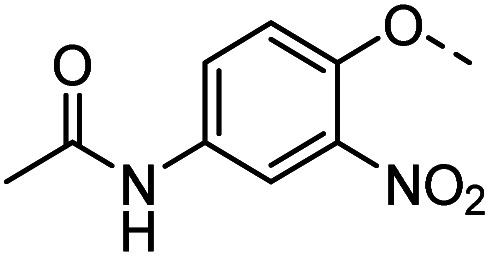
36	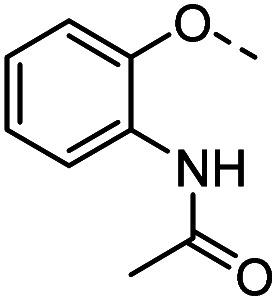	42	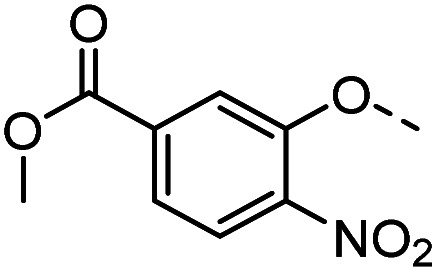	48	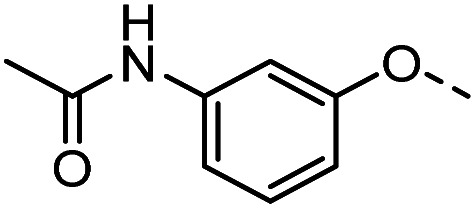	54	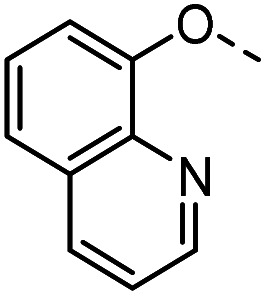
37	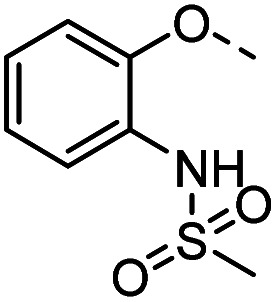	43	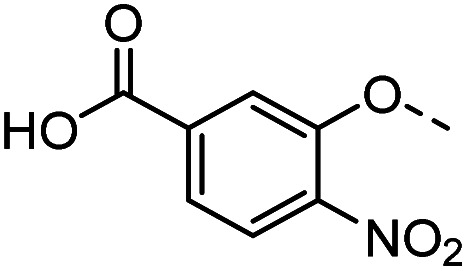	49	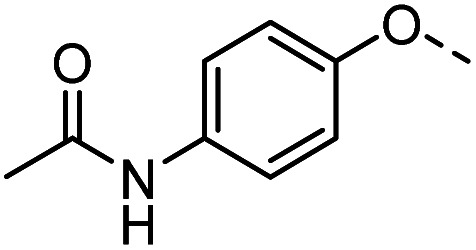	55	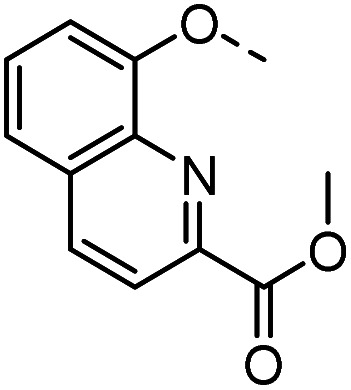
38	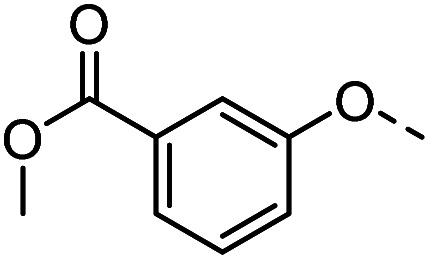	44	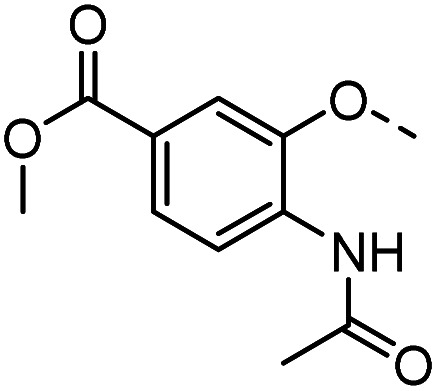	50	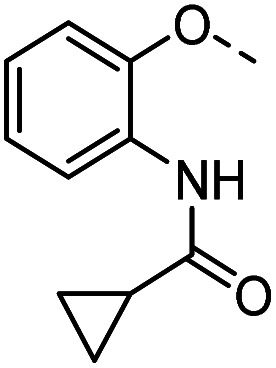	56	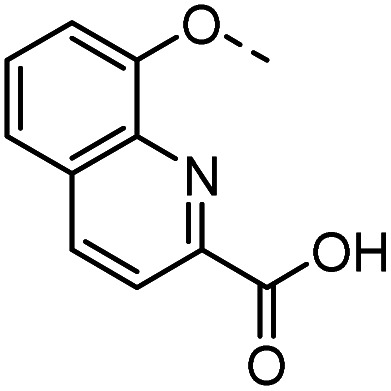

### 2.2 Galectin binding and structure–activity relationships

The affinities of compounds 33–56 for galectin-1, -3, and -8N were determined in a competitive fluorescence anisotropy assay (Table 2).^12,37,38^ Affinities of compounds 33–56 varied significantly between different compounds and between galectins-1, -3, and -8N. Several compounds showed binding well below 1 mM and inter-galectin selectivity indicates specific interactions in the binding sites.

**Table 2:** *K*_d_ values^a^ (μM) obtained by competitive fluorescence anisotropy

Compound	*K*_d_ [μM]		
	Galectin-1	Galectin-3	Galectin-8N
Lactose^41–43^	104	231	91
33	>1500	300 ± 33	>1500
34	550 ± 57	490 ± 43	650 ± 67
35	>1500	1500 ± 80	670 ± 26
36	470 ± 35	810 ± 58	>1500
37	>1500	1500 ± 290	>1500
38	570 ± 110	530 ± 210	910 ± 48
39	1500 ± 180	650 ± 55	>1500
40	840 ± 66	>1500	>1500
41	1100 ± 81	210 ± 55	1500 ± 47
42	N.B.^b^	>1500	>1500
43	>1500	270 ± 43	1400 ± 270
44	>1500	N.B.^b^	N.B.^b^
45	1100 ± 160	1400 ± 57	>1500
46	1200 ± 67	340 ± 40	930 ± 78
47	1300 ± 150	1100 ± 160	>1500
48	>1500	700 ± 140	990 ± 270
49	>1500	1400 ± 360	>1500
50	490 ± 120	500 ± 28	1100 ± 16
51	390 ± 53	320 ± 75	1100 ± 200
52	1300 ± 270	N.B.^b^	440 ± 60
53	520 ± N. A.	850 ± 330	670 ± 120
54	N.B.^b^	N.B.^b^	N.B.^b^
55	980 ± 200	N.B.^b^	N.B.^b^
56	>1500	420 ± 180	1100 ± 140

a Results represent the mean ± SEM of *n* = 4 to 8.

b Non-binding up to the highest tested concentration of at least 1200 μM.

A common feature of many of the compounds with high affinity for the three galectins is an *ortho*-amide moiety present in 36 and 50–52. Molecular docking calculations in FRED (OpenEye Scientific Software Inc.) revealed that an *ortho*-amide carbonyl can form a hydrogen bond in the CRDs of galectin-1, -3 and -8N (see below). Replacement of the *ortho*-acetamide in 36 by a sulphonamide in 37 or methylamine in 35 was detrimental for binding. Moreover, changing the position of the acetamide group from *ortho* in 36 to *meta* in 48 or *para* in 49 did not improve binding affinity. In general, introduction of nitro group increased binding affinity (34*vs.*45, 53*vs.*49 and 43*vs.*39), but not in the ester pair of 42 and 39. Several compounds carrying a carboxylic acid at *meta* position, such as 39, 41, 43 and 56 showed selectivity for galectin-3. The importance of the carboxylic acid for binding galectin-3 is exemplified by the weak binding (>1500) of analogous esters (compare 40*vs.*41 and 42*vs.*43). However, methyl ester 38 displayed comparable potencies against all three galectins tested and was practically equipotent with its free carboxylate analogue 39. Removal of the carboxylic acid group in 39 resulted in the weakly binding 45. Introduction of *ortho*-hydroxyl (46) slightly improved binding to galectin-3 compared to 45, while compound 47 carrying the *ortho*-hydroxymethyl group showed poor affinity for all three galectins. Docking calculations showed that most phenyl groups of 33-56 can stack with the Arg144 of galectin-3, analogously to reported fluorophenyl thiodigalactoside 4.^39^ The carboxylates 39, 41, 43 and 56 also appear to form ionic interaction with Arg144 in galectin-3. Replacement of the phenyl moiety by quinoline did not result in improved binding of compounds 54–56.

### 2.3 Optimisation of compound 36

**Scheme 1:**
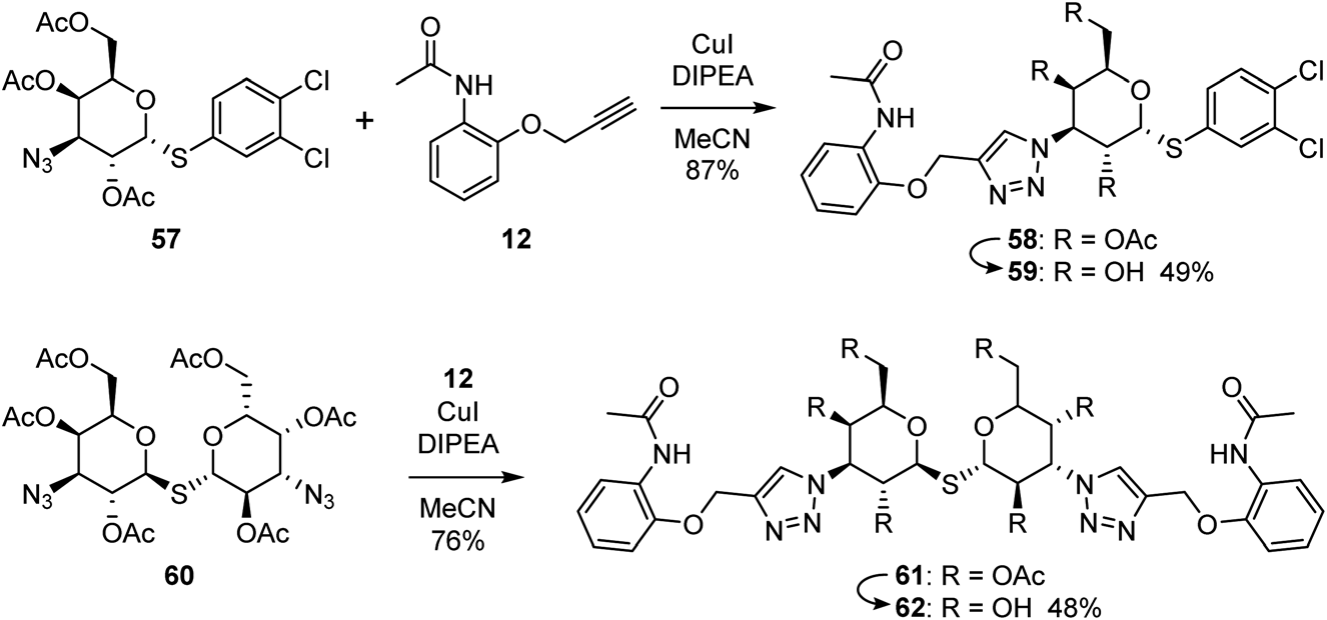
Synthesis of optimised acetamide ligands.

To confirm the effectivity of the screening strategy, we selected the smallest of the *ortho*-acetamide analogues (36) and synthesised two analogues based on the known affinity-enhancing structural elements positioned in subsites D and E; the dichlorophenyl thio-α-galactosides (6 and 7, Fig. 2) and the thiodigalactosides (2–5, Fig. 2). The dichlorophenyl 3-azido-1-thio-α-galactoside 57,^40^ was subjected to Cu(i)-catalysed cycloaddition with the alkyne 12 followed by deprotection of the hydroxyl groups of 58 to give the analogue 59 (Scheme 1). Similarly, the thiodigalactoside analogue 62 was prepared from the 3,3′-diazidothiodigalactoside 60.^12^

The affinity of the dichlorothiophenyl-based compound 59 for galectin-3 was increased around 60-fold, thus inverting the initial selectivity for galectin-1 as expected (Table 3). The affinity of 59 for galectin-8N also drastically improved. The increase of affinity was even more remarkable for the thiodigalactoside 62, in particular for galectin-3, yielding a nanomolar affinity ligand from the original hit 36.

**Table 3:** *K*_d_ values^a^ (μM) obtained by competitive fluorescence anisotropy

Compound	*K*_d_ [μM]		
	Galectin-1	Galectin-3	Galectin-8N
36	470 ± 35	810 ± 58	>2000
59	89 ± 10	14 ± 1.2	100 ± 9
62	3.3 ± 0.4	0.42 ± 0.03	150 ± 27

a Results represent the mean ± SEM of *n* = 4 to 8.

### 2.4 Molecular dynamics simulations

**Fig. 3:**
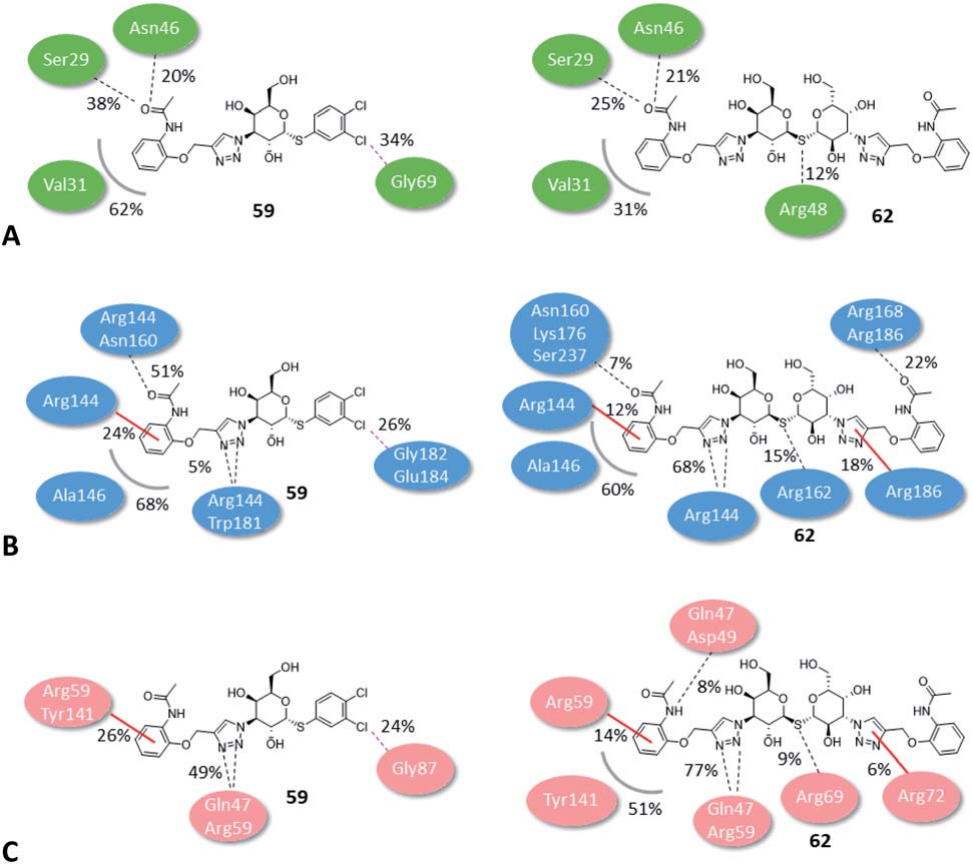
Schematic representation of interaction analysis from MD simulations. (A) Galectin-1, (B) galectin-3 and (C) galectin-8N. Hydrogen and halogen bonds are shown as black and pink dashed lines, respectively. Cation–π interaction is presented as red line, while hydrophobic interactions are presented as grey curved line. For clarity of presentation, interactions with galactose moieties are not shown.

**Fig. 4:**
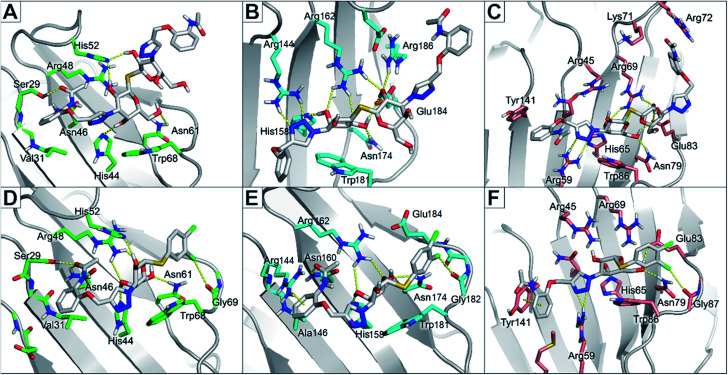
Molecular dynamics snapshots of compounds 62 and 59 in galectin-1, -3, and -8N. (A) and (D) The CRD of galectin-1 with compounds 62 and 59, respectively. (B) and (E) The CRD of galectin-3 with compounds 62 and 59, respectively. (C) and (F) The CRD of galectin-8N with compounds 62 and 59, respectively. Ligands are shown as white sticks with standard atom colouring. Residues are shown as sticks carbon-coloured green (galectin-1), cyan (galectin-3), and salmon (galectin-8N).

The binding modes of the most potent compounds 59 and 62 at binding sites of galectin-1, -3 and -8N were investigated by docking followed by molecular dynamics (MD) simulations (Fig. 3 and 4) to evaluate the complex stability and ligand interactions during a simulation time of 200 ns. The MD simulations showed that the galactose moiety binds consistently to the C-subsite of the CRDs in all three galectins (Fig. 4).

However, subtle differences were found in the binding modes of other structural elements of 59 and 62 in the galectin-1, -3 and -8N complexes. The interaction features of the ligands in the galectin CRDs during the MD simulation were analysed using the MD analysis tool in LigandScout 4.4 Expert.^44^ The results are summarized in Fig. 3, while representative snapshots from MD trajectories are presented in Fig. 4.

In the AB subsite, the acetamide group of compound 62 in complex with galectin-1 (Fig. 3A and 4A) forms hydrogen bonds with Ser29 and Asn46, while the phenyl ring forms hydrophobic interactions with the Val31 side chain. Similar interactions were observed also in the galectin-3 and -8N CRDs, but they differed in their occurrence during the MD trajectories (Fig. 3B and C and Fig. 4B and 4C). In addition, the thioether linking the two galactose moieties of 62 forms a hydrogen bond with the Arg48, Arg162 and Arg69 side chains in galectin-1, -3 and -8N, respectively. Differences can be observed in the case of the triazole and phenylacetamide moieties binding in the E subsite. In galectin-1 and -8N, this part of 62 is in contact with the protein only for a short time, but in case of galectin-3 it forms a cation–π interaction with Arg186 and hydrogen bonds with Arg168 and Arg186 (Fig. 3B and 4B), which may explain its stronger affinity for galectin-3 (Table 3). The overall higher affinity of thiodigalactoside 62 is attributed to the hydrogen bond interactions in the D subsite, analogous to known inhibitors.^12^ Compound 59 forms the most extensive network of interactions in the AB subsite of galectin-3. Its phenylacetamide moiety is tightly bound with hydrogen bonds with Arg144 and Asn160, cation–π interactions with Arg144 side chain and hydrophobic interactions with Ala146 (Fig. 3B and 4E). These interactions are less pronounced in the case of galectin-1 and -8N (Fig. 3A and C and Fig. 4D and F). Compound 59 forms additional halogen bond in the D subsite in all three galectins. Taken together, these interaction analysis results are in agreement with the most potent binding of 59 to galectin-3 CRD (Table 3).

## 3 Conclusions

A collection of 24 C3-triazolyl-1-thiogalactosides was synthesised to investigate subsites A and B of galectins-1, -3, and -8N, using an efficient synthetic approach using available building blocks and reliable chemistry. The flexible phenoxymethyl group carrying common functionalities with variable substitution was attached to the position 4 of the triazole. These inhibitors generally showed the highest affinity for galectin-3. Especially the inhibitors carrying larger, polysubstituted phenyls have a higher binding for galectin-3 compared to galectins-1 and -8N. The phenylacetamide was identified as the smallest member of the promising amide functionalisation and was further optimised using established affinity enhancing strategies. Synthesis and analysis of two optimised structures resulted in thiodigalactoside 62 with nanomolar affinity for galectin-3 and is a prototype pan-galectin inhibitor. The compound still lacks potency for galectin-8, but nevertheless suggests how such an inhibitor can be developed.

## 4 Experimental section

### 4.1 Chemistry

All reagents and solvents were dried before use according to standard methods. Commercial reagents were used without further purification. Unless stated otherwise all reactions were performed in inert atmosphere. TLC analysis was performed on precoated Merck silica gel 60 F254 plates using UV light and charring solution (1 : 9 H_2_SO_4_/EtOH). Flash column chromatography was performed on SiO_2_ purchased from Aldrich (technical grade, 60 Å pore size, 230–400 mesh, 40–63 mm). Preparative HPLC was performed on an Agilent 1260 Infinity system with a SymmetryPrep C18, 5 μM, 19 mm 100 mm column using a gradient (water with 0.1% formic acid and acetonitrile). Monitoring and collection were based on UV absorbance at 210 and 254 nm. NMR spectra ^1^H, ^13^C, 2D COSY, and HMQC were recorded with a Bruker Avance II 400 MHz spectrometer (400 Hz for ^1^H, 100 Hz for ^13^C) or a Bruker Avance III 500 MHz spectrometer (500 Hz for ^1^H, 125 Hz for ^13^C) at ambient temperature. Chemical shifts are reported in *δ* parts per million (ppm), with multiplicity (b = broad, s = singlet, d = doublet, t = triplet, q = quartet, quin = quintet, hept = heptet, m = multiplet, app = apparent), coupling constants (in Hz) and integration. High-resolution mass analyses were performed using a Micromass Q-TOF mass spectrometer (ESI). Purities of final compounds were determined by UPLC (Waters Acquity UPLC system, column Waters Acquity CSHC18, 0.5 mL min-1, H_2_O–MeCN gradient 5–95% 10 min with 0.1% formic acid). Analytical data are given if the compound is novel or not fully characterised in the literature.

#### General procedure A

Tolyl 3-azido-3-deoxy-1-thio-β-d-galactopyranoside 8 (1 equiv.), sodium ascorbate (0.4 equiv.), copper sulphate (0.2 equiv.) and the respective propargyl derivative (1.2 equiv.) were suspended in 5–15 mL H_2_O/MeOH (2 : 3). The reaction mixture was stirred at r. t. overnight. The mixture was filtered and the filtrate concentrated *in vacuo*. The products were isolated using column chromatography and/or preparative HPLC.

#### General procedure B

Tolyl 3-azido-3-deoxy-1-thio-β-d-galactopyranoside 8 (1 equiv.), copper iodide (0.2 equiv.), *N*,*N*-diisopropylethylamine (DIPEA) (1.5 equiv.) and the respective propargyl derivative (1.2 equiv.) were suspended in 5–15 mL H_2_O/MeOH (2 : 3). The reaction mixture was stirred at r. t. overnight. The mixture was filtered, and the filtrate concentrated *in vacuo*. The products were isolated using column chromatography and/or preparative HPLC.

#### Tolyl 3-(4-((2-aminophenoxy)methyl)-1,2,3-triazol-1-yl)-3-deoxy-1-thio-β-d-galactopyranoside (33)

The compound was prepared according to general procedure B using 8 (55 mg, 0.18 mmol) and 10 (45 mg, 0.31 mmol). Column chromatography (1 : 20 MeOH/DCM) gave product 33 as a yellow solid (52 mg, 64% yield). ^1^H NMR (400 MHz, MeOD) *δ* 8.16 (s, 1H), 7.53–7.45 (m, 2H), 7.15 (d, *J* = 7.9 Hz, 2H), 7.00 (d, *J* = 7.9 Hz, 1H), 6.80–6.75 (m, 2H), 6.73–6.65 (m, 1H), 5.19 (s, 2H), 4.84 (dd, *J* = 10.6, 3.0 Hz, 1H), 4.75 (d, *J* = 9.5 Hz, 1H), 4.20 (dd, *J* = 10.5, 9.6 Hz, 1H), 4.11 (d, *J* = 2.9 Hz, 1H), 3.82–3.66 (m, 3H), 2.33 (s, 3H). ^13^C NMR (101 MHz, MeOD) *δ* 147.82, 144.70, 138.78, 138.12, 133.21, 131.57, 130.64, 124.83, 122.95, 119.72, 117.09, 113.73, 91.66, 80.90, 69.56, 69.11, 67.93, 63.07, 62.28, 49.00, 21.11. HRMS (ESI+) calcd for C_22_H_27_N4O_5_S [M + H]^+^: 459.1697, measured: 459.1691; HPLC purity: 96.5%.

#### Tolyl 3-(4-((2-nitrophenoxy)methyl)-1,2,3-triazol-1-yl)-3-deoxy-1-thio-β-d-galactopyranoside (34)

The compound was prepared according to general procedure A using 8 (56 mg, 0.18 mmol) and 9 (35 mg, 0.20 mmol). Column chromatography (2 : 1 EtOAc/Hex.) gave product 34 as a slightly yellow solid (29 mg, 33% yield). ^1^H NMR (400 MHz, DMSO-*d*_6_) *δ* 8.18 (s, 1H), 7.87 (dd, *J* = 8.0, 1.6 Hz, 1H), 7.73–7.60 (m, 2H), 7.41 (d, *J* = 8.1 Hz, 2H), 7.17–7.12 (m, 3H), 5.61 (d, *J* = 7.4 Hz, 1H), 5.39–5.32 (s, 2H), 5.24 (d, *J* = 6.9 Hz, 1H), 4.90–4.80 (m, 2H), 4.71 (t, *J* = 5.5 Hz, 1H), 4.11–4.03 (m, 1H), 3.91 (dd, *J* = 6.9 Hz, 2.9 Hz, 1H), 3.76 (t, *J* = 6.3 Hz, 1H), 3.50 (t, *J* = 5.8 Hz, 2H), 2.29 (s, 3H); HRMS (ESI+) calcd for C_22_H_25_N_4_O_7_S [M + H]^+^: 489.1439, measured: 489.1436; HPLC: purity: >99.9%.

#### Tolyl 3-(4-((2-methylaminophenoxy)methyl)-1,2,3-triazol-1-yl)-3-deoxy-1-thio-β-d-galactopyranoside (35)

The compound was prepared according to general procedure B using 8 (60 mg, 0.19 mmol) and 11 (50 mg, 0.31 mmol). Column chromatography (2 : 1 EtOAc/Hex.) gave product 35 as a slightly yellow solid (55 mg, 60% yield). ^1^H NMR (400 MHz, MeOD) *δ* 8.16 (s, 3H), 7.55–7.44 (m, 6H), 7.15 (d, *J* = 7.9 Hz, 6H), 7.01–6.92 (m, 3H), 6.87 (td, *J* = 7.7, 1.3 Hz, 3H), 6.68–6.57 (m, 6H), 5.16 (s, 6H), 4.84 (dd, *J* = 10.6, 3.0 Hz, 4H), 4.75 (d, *J* = 9.5 Hz, 3H), 4.21 (dd, *J* = 10.4, 9.6 Hz, 3H), 4.10 (d, *J* = 2.9 Hz, 3H), 3.82–3.67 (m, 9H), 2.78 (s, 9H), 2.33 (s, 9H). ^13^C NMR (101 MHz, MeOD) *δ* 145.97, 143.26, 139.80, 137.39, 131.80, 130.18, 129.25, 123.46, 121.79, 116.29, 111.20, 109.87, 90.25, 79.49, 68.19, 67.72, 66.54, 61.60, 60.90, 29.27, 19.71. HRMS (ESI+) calcd for C_23_H_29_N_4_O_5_S [M + H]^+^: 473.1853, found: 473.1849; HPLC purity: 98.2%.

#### Tolyl 3-(4-((2-acetamidophenoxy)methyl)-1,2,3-triazol-1-yl)-3-deoxy-1-thio-β-d-galactopyranoside (36)

The compound was prepared according to general procedure B using 8 (52 mg, 0.16 mmol) and 12 (35 mg, 0.18 mmol). Column chromatography (1 : 20 MeOH/DCM) gave product 36 as a slightly yellow solid (40 mg, 48% yield). ^1^H NMR (400 MHz, DMSO-*d*_6_) *δ* 9.07 (s, 1H), 8.21 (s, 1H), 7.87 (d, *J* = 7.8 Hz, 1H), 7.40 (d, *J* = 8.1 Hz, 2H), 7.33–7.26 (m, 1H), 7.16 (d, *J* = 8.0 Hz, 2H), 7.07 (t, *J* = 7.4 Hz, 1H), 6.92 (t, *J* = 7.7 Hz, 1H), 5.59 (d, *J* = 7.4 Hz, 1H), 5.26–5.18 (m, 3H), 4.88–4.79 (m, 2H), 4.73 (t, *J* = 5.6 Hz, 1H), 4.14–4.04 (m, 1H), 3.91 (dd, *J* = 6.9, 2.7 Hz, 1H), 3.76 (t, *J* = 6.3 Hz, 1H), 3.50 (t, *J* = 5.9 Hz, 2H), 2.29 (s, 3H), 2.07 (s, 3H). ^13^C NMR (101 MHz, DMSO-*d*_6_) *δ* 168.44, 148.91, 142.20, 136.24, 130.98, 130.33, 129.62, 127.80, 124.43, 124.01, 122.69, 120.82, 113.41, 89.06, 79.23, 67.63, 66.98, 66.22, 62.52, 60.23, 39.52, 23.91, 20.64. HRMS (ESI+) calcd for C_24_H_29_N_4_O_6_S [M + H]^+^: 501.1808, found: 501.1809; HPLC purity: >99.9%.

#### Tolyl 3-(4-((methylsulfonamidophenyloxy)methyl)-1,2,3-triazol-1-yl)-3-deoxy-1-thio-β-d-galactopyranoside (37)

The compound was prepared according to general procedure A using 8 (68 mg, 0.22 mmol) and 13 (68 mg, 0.30 mmol). Column chromatography (EtOAc) gave product 37 as a white amorphous solid (45 mg, 38% yield). ^1^H NMR (400 MHz, MeOD) *δ* 8.14 (s, 1H), 7.88 (dd, *J* = 8.0, 1.5 Hz, 1H), 7.53–7.46 (m, 2H), 7.22–7.07 (m, 4H), 6.95 (td, *J* = 7.9, 1.3 Hz, 1H), 5.25 (s, 2H), 4.84 (dd, *J* = 10.6, 3.0 Hz, 1H), 4.75 (d, *J* = 9.5 Hz, 1H), 4.19 (dd, *J* = 10.4, 9.7 Hz, 1H), 4.10 (d, *J* = 2.9 Hz, 1H), 3.82–3.66 (m, 3H), 2.33 (s, 3H), 2.14 (s, 3H). ^13^C NMR (101 MHz, MeOD) *δ* 171.90, 150.46, 144.12, 138.81, 133.25, 131.52, 130.65, 128.75, 126.40, 124.94, 124.28, 122.35, 114.07, 91.62, 80.91, 69.57, 69.12, 67.94, 63.26, 62.30, 49.00, 23.72, 21.11. HRMS (ESI−) calcd for C_23_H_27_N_4_O_7_S_2_ [M − H]^−^: 535.1327, found: 535.1324; HPLC: purity: 99.7%.

#### Tolyl 3-(4-((3-methoxycarbonylphenyloxy)methyl)-1,2,3-triazol-1-yl)-3-deoxy-1-thio-β-d-galactopyranoside (38)

The compound was prepared according to general procedure B using 8 (100 mg, 0.32 mmol) and 14 (90 mg, 0.47 mmol). Column chromatography (1 : 20 MeOH/DCM) gave product 38 as a slightly yellow solid (132 mg, 82% yield). ^1^H NMR (400 MHz, DMSO-*d*_6_) *δ* 8.24 (s, 1H), 7.64 (d, *J* = 8.5 Hz, 2H), 7.52 (t, *J* = 7.9 Hz, 1H), 7.47 (d, *J* = 8.0 Hz, 2H), 7.41 (dd, *J* = 8.2, 1.4 Hz, 1H), 7.21 (d, *J* = 8.0 Hz, 2H), 5.69 (d, *J* = 7.3 Hz, 1H), 5.32 (d, *J* = 6.9 Hz, 1H), 5.27 (s, 2H), 4.98–4.86 (m, 2H), 4.81 (t, *J* = 5.5 Hz, 1H), 4.15 (dd, *J* = 17.5, 9.7 Hz, 1H), 3.98 (dd, *J* = 6.6, 2.3 Hz, 1H), 3.91 (s, 3H), 3.83 (t, *J* = 6.1 Hz, 1H), 3.57 (t, *J* = 5.7 Hz, 2H), 2.35 (s, 3H). ^13^C NMR (101 MHz, DMSO-*d*_6_) *δ* 166.51, 158.72, 142.05, 136.67, 131.48, 131.45, 130.77, 130.49, 130.05, 124.50, 122.16, 120.40, 115.21, 89.52, 79.67, 68.08, 67.36, 66.64, 61.88, 60.69, 52.74, 21.07. HRMS (ESI+) calcd for C_24_H_28_N_3_O_7_S [M + H]^+^: 502.1642, found: 502.1637; HPLC purity: >99.9%.

#### Tolyl 3-(4-((3-carboxyphenyloxy)methyl)-1,2,3-triazol-1-yl)-3-deoxy-1-thio-β-d-galactopyranoside (39)

Compound 38 (60 mg, 0.12 mmol) was dissolved in acetonitrile (6 mL). NaOH_aq_ (1.0 M, 6 mL) was added and the mixture was heated to 50 °C for 1 h. At r. t., HCl_aq_ (2.0 M, 6 mL) was added. The product was filtered off as a precipitate and dried *in vacuo* to give 39 as a white solid (58 mg, 99% yield). ^1^H NMR (400 MHz, DMSO-*d*_6_) *δ* 13.02 (s, 1H), 8.18 (s, 1H), 7.56 (d, *J* = 8.8 Hz, 2H), 7.43 (dd, *J* = 16.1, 8.1 Hz, 3H), 7.32 (d, *J* = 7.8 Hz, 1H), 7.16 (d, *J* = 7.9 Hz, 2H), 5.63 (d, *J* = 7.3 Hz, 1H), 5.26 (d, *J* = 6.9 Hz, 1H), 5.20 (s, 2H), 4.86 (dd, *J* = 14.1, 5.8 Hz, 2H), 4.74 (s, 1H), 4.09 (dd, *J* = 17.5, 9.7 Hz, 1H), 3.92 (d, *J* = 4.3 Hz, 1H), 3.77 (t, *J* = 6.0 Hz, 1H), 3.50 (s, 2H), 2.29 (s, 3H). ^13^C NMR (101 MHz, DMSO-*d*_6_) *δ* 167.58, 158.66, 142.10, 136.66, 132.69, 131.47, 130.76, 130.25, 130.05, 124.48, 122.32, 119.95, 115.30, 89.52, 79.68, 68.08, 67.35, 66.64, 61.79, 60.69, 21.07. HRMS calcd for C_23_H_26_N_3_O_7_S [M + H]^+^: 488.1486, found: 488.1473; HPLC purity: >99.9%.

#### Tolyl 3-(4-((2-amino-5-methoxycarbonyl-phenyloxy)methyl)-1,2,3-triazol-1-yl)-3-deoxy-1-thio-β-d-galactopyranoside (40)

The compound was prepared according to general procedure B using 8 (80 mg, 0.26 mmol) and 16 (60 mg, 0.29 mmol). Column chromatography (1 : 20 MeOH/DCM) gave product 40 as an off-white solid (82 mg, 62% yield). ^1^H NMR (400 MHz, DMSO-*d*_6_) *δ* 8.18 (s, 1H), 8.13 (d, *J* = 1.5 Hz, 1H), 8.00 (d, *J* = 8.3 Hz, 1H), 7.70 (dd, *J* = 8.3, 1.6 Hz, 1H), 7.41 (d, *J* = 8.1 Hz, 2H), 7.16 (d, *J* = 8.0 Hz, 2H), 5.63 (d, *J* = 7.4 Hz, 1H), 5.45 (s, 2H), 5.26 (d, *J* = 6.9 Hz, 1H), 4.90–4.80 (m, 2H), 4.73 (t, *J* = 5.6 Hz, 1H), 4.12–4.02 (m, 1H), 3.96–3.87 (m, 4H), 3.76 (t, *J* = 6.3 Hz, 1H), 3.50 (t, *J* = 5.9 Hz, 2H), 2.29 (s, 3H). ^13^C NMR (101 MHz, DMSO-*d*_6_) *δ* 165.25, 150.81, 143.00, 141.16, 136.68, 134.63, 131.40, 130.81, 130.05, 125.56, 124.87, 122.14, 116.54, 89.47, 79.66, 68.01, 67.38, 66.63, 63.40, 60.68, 53.38, 21.07. HRMS (ESI+) for C_24_H_29_N_4_O_7_S [M + H]^+^: calculated: 517.1751, measured: 517.1746; HPLC purity: 99.2%.

#### Tolyl 3-(4-((2-amino-5-carboxy-phenyloxy)methyl)-1,2,3-triazol-1-yl)-3-deoxy-1-thio-β-d-galactopyranoside (41)

Compound 40 (38 mg, 0.07 mmol) was dissolved in acetonitrile (6 mL). NaOH_aq_ (1.0 M, 6 mL) was added and the mixture was heated to 50 °C for 1 h. At r. t., HCl_aq_ (2.0 M, 6 mL) was added. The product was filtered off as a precipitate and dried *in vacuo* to give 41 as a white solid (37 mg, 99% yield). ^1^H NMR (400 MHz, DMSO-*d*_6_) *δ* 8.17 (s, 1H), 8.10 (s, 1H), 7.97 (d, *J* = 8.3 Hz, 1H), 7.68 (d, *J* = 8.3 Hz, 1H), 7.41 (d, *J* = 8.1 Hz, 2H), 7.16 (d, *J* = 8.1 Hz, 2H), 5.63 (d, *J* = 7.4 Hz, 1H), 5.43 (s, 2H), 5.27 (d, *J* = 6.9 Hz, 1H), 4.92–4.79 (m, 2H), 4.73 (t, *J* = 5.4 Hz, 1H), 4.07 (td, *J* = 9.8, 7.4 Hz, 1H), 3.91 (dd, *J* = 6.7, 2.8 Hz, 1H), 3.76 (t, *J* = 6.2 Hz, 1H), 3.50 (t, *J* = 5.6 Hz, 2H), 2.29 (s, 3H). ^13^C NMR (101 MHz, DMSO-*d*_6_) *δ* 166.29, 150.81, 142.75, 141.23, 136.68, 136.09, 131.40, 130.81, 130.05, 125.42, 124.84, 122.20, 116.54, 89.47, 79.66, 68.02, 67.38, 66.63, 63.30, 60.69, 21.08. HRMS (ESI+) for C_23_H_27_N_4_O_7_S [M + H]^+^: calculated: 503.1595, measured: 503.1593; HPLC purity: 97.0%.

#### Tolyl 3-(4-((5-methoxycarbonyl-2-nitrophenyloxy)methyl)-1,2,3-triazol-1-yl)-3-deoxy-1-thio-β-d-galactopyranoside (42)

The compound was prepared according to general procedure B using 8 (60 mg, 0.19 mmol) and 15 (40 mg, 0.17 mmol). Column chromatography (1 : 20 MeOH/DCM) gave product 42 as a white solid (68 mg, 73% yield). ^1^H NMR (400 MHz, DMSO-*d*_6_) *δ* 8.18 (s, 1H), 8.13 (d, *J* = 1.5 Hz, 1H), 8.00 (d, *J* = 8.3 Hz, 1H), 7.70 (dd, *J* = 8.3, 1.5 Hz, 1H), 7.41 (d, *J* = 8.1 Hz, 2H), 7.16 (d, *J* = 8.0 Hz, 2H), 5.63 (d, *J* = 7.4 Hz, 1H), 5.51–5.40 (m, 2H), 5.26 (d, *J* = 6.9 Hz, 1H), 4.91–4.79 (m, 2H), 4.73 (t, *J* = 5.6 Hz, 1H), 4.07 (td, *J* = 9.9, 7.6 Hz, 1H), 3.97–3.85 (m, 4H), 3.76 (t, *J* = 6.2 Hz, 1H), 3.50 (t, *J* = 5.9 Hz, 2H), 2.29 (s, 3H). ^13^C NMR (101 MHz, DMSO-*d*_6_) *δ* 165.25, 150.81, 143.00, 141.19, 136.68, 134.63, 131.40, 130.81, 130.04, 125.56, 124.89, 122.14, 116.54, 89.47, 79.66, 68.01, 67.39, 66.64, 63.40, 60.68, 53.36, 21.07. HRMS (ESI+) calcd for C_24_H_27_N_4_O_9_S [M + H]^+^: 547.1493, found: 547.1487; HPLC purity: 98.6%.

#### Tolyl 3-(4-((5-carboxy-2-nitrophenyloxy)methyl)-1,2,3-triazol-1-yl)-3-deoxy-1-thio-β-d-galactopyranoside (43)

Compound 42 (35 mg, 0.07 mmol) was dissolved in acetonitrile (6 mL). NaOH_aq_ (1.0 M, 6 mL) was added and the mixture was heated to 50 °C for 1 h. At r. t., HCl_aq_ (2.0 M, 6 mL) was added. The product was filtered off as a precipitate and dried *in vacuo* to give 43 as a yellow solid (33 mg, 97% yield). ^1^H NMR (400 MHz, DMSO-*d*_6_) *δ* 13.66 (s, 1H), 8.17 (s, 4H), 8.11 (d, *J* = 1.5 Hz, 4H), 7.97 (d, *J* = 8.3 Hz, 4H), 7.68 (dd, *J* = 8.3, 1.5 Hz, 4H), 7.41 (d, *J* = 8.1 Hz, 8H), 7.16 (d, *J* = 8.0 Hz, 8H), 5.63 (d, *J* = 7.3 Hz, 4H), 5.44 (s, 8H), 5.26 (d, *J* = 6.9 Hz, 4H), 4.90–4.80 (m, 8H), 4.73 (s, 4H), 4.12–4.01 (m, 4H), 3.91 (dd, *J* = 6.9, 2.9 Hz, 4H), 3.76 (t, *J* = 6.3 Hz, 4H), 3.53–3.46 (m, 8H), 2.29 (s, 12H). ^13^C NMR (101 MHz, DMSO-*d*_6_) *δ* 166.28, 150.80, 142.76, 141.23, 136.68, 136.04, 131.40, 130.81, 130.05, 125.42, 124.83, 122.20, 116.54, 89.47, 79.66, 68.01, 67.38, 66.64, 63.31, 60.69, 21.08. HRMS (ESI+) calcd for C_23_H_25_N_4_O_9_S [M + H]^+^: 533.1337, found: 533.1326; HPLC purity: 85.9%.

#### Tolyl 3-(4-((2-acetamido-5-methoxycarbonyl-phenyloxy)methyl)-1,2,3-triazol-1-yl)-3-deoxy-1-thio-β-d-galactopyranoside (44)

The compound was prepared according to general procedure A using 8 (43 mg, 0.14 mmol) and 18 (41 mg, 0.17 mmol). Column chromatography (1 : 8 EtOAc/Hex.) gave product 44 as an off-white solid (36 mg, 47% yield). ^1^H NMR (400 MHz, DMSO-*d*_6_) *δ* 9.28 (s, 1H), 8.25–8.15 (m, 2H), 7.84 (d, *J* = 1.8 Hz, 1H), 7.59 (dd, *J* = 8.4, 1.7 Hz, 1H), 7.41 (d, *J* = 8.1 Hz, 2H), 7.16 (d, *J* = 8.0 Hz, 2H), 5.56 (d, *J* = 7.4 Hz, 1H), 5.37–5.28 (m, 2H), 5.21 (d, *J* = 6.8 Hz, 1H), 4.89–4.79 (m, 2H), 4.72 (t, *J* = 5.6 Hz, 1H), 4.08 (td, *J* = 9.7, 7.5 Hz, 1H), 3.92 (dd, *J* = 6.8, 2.9 Hz, 1H), 3.84 (s, 3H), 3.76 (t, *J* = 6.3 Hz, 1H), 3.50 (t, *J* = 5.8 Hz, 2H), 2.29 (s, 3H), 2.13 (s, 3H); HRMS (ESI+) for C_26_H_31_N_4_O_8_S [M + H]^+^: calculated 559.1857, measured 559.1855; HPLC purity: >99.9%.

#### Tolyl 2,4,6-tri-*O*-acetyl-3-(4-(phenoxymethyl)-1,2,3-triazol-1-yl)-3-deoxy-1-thio-β-d-galactopyranoside (45)

The compound was prepared according to general procedure B using tolyl 2,4,6-tri-*O*-acetyl-3-azido-3-deoxy-1-thio-β-d-galactopyranoside (90 mg, 0.21 mmol) and 19 (30 mg, 0.23 mmol). Column chromatography (1 : 9 DCM/EtOAc) gave intermediate tolyl 2,4,6-tri-*O*-acetyl-3-(4-(phenoxymethyl)-1,2,3-triazol-1-yl)-3-deoxy-1-thio-β-d-galactopyranoside (45a) as a white solid (96 mg, 82% yield). ^1^H NMR (400 MHz, CDCl_3_) *δ* 7.60 (s, 1H), 7.45–7.40 (m, 2H), 7.30–7.24 (m, 2H), 7.15 (d, *J* = 7.9 Hz, 2H), 7.01–6.89 (m, 3H), 5.63 (dd, *J* = 11.0, 9.7 Hz, 1H), 5.49 (dd, *J* = 3.1, 0.7 Hz, 1H), 5.20 (s, 2H), 5.13 (dd, *J* = 11.0, 3.2 Hz, 1H), 4.77 (d, *J* = 9.7 Hz, 1H), 4.17–4.09 (m, 2H), 4.09–4.02 (m, 1H), 2.36 (s, 3H), 2.03 (s, 3H), 1.90 (s, 3H), 1.86 (s, 3H). Intermediate 45a (88 mg, 0.15 mmol) was suspended in MeOH (6 mL). Sodium methoxide (0.45 mL, 30 wt% in MeOH, 2.40 mmol) was added and the mixture stirred at room temperature for 1 h. Amberlite® IRC120 H was added, and the mixture stirred for another 10 minutes before filtering and removing the solvents *in vacuo*. Column chromatography (1 : 20 MeOH/DCM) gave 45 as a white solid (50 mg, 73% yield). ^1^H NMR (400 MHz, MeOD) *δ* 8.15 (s, 1H), 7.53–7.48 (m, 2H), 7.33–7.26 (m, 2H), 7.16 (d, *J* = 7.9 Hz, 2H), 7.05–7.00 (m, 2H), 7.00–6.93 (m, 1H), 5.18 (s, 2H), 4.86–4.83 (m, 1H), 4.76 (d, *J* = 9.5 Hz, 1H), 4.21 (dd, *J* = 10.5, 9.6 Hz, 1H), 4.12 (d, *J* = 2.9 Hz, 1H), 3.83–3.68 (m, 3H), 2.34 (s, 3H). ^13^C NMR (101 MHz, MeOD) *δ* 158.48, 143.12, 137.39, 131.81, 130.18, 129.24, 129.14, 123.45, 120.81, 114.36, 90.29, 79.52, 68.16, 67.69, 66.55, 60.96, 60.89, 19.70. HRMS (ESI+) calcd for C_22_H_25_O_5_N_3_S [M + H]^+^: 444.1588, found: 444.1592; HPLC purity: 99.2%.

#### Tolyl 3-(4-((2-hydroxyphenyl)methyl)-1,2,3-triazol-1-yl)-3-deoxy-1-thio-β-d-galactopyranoside (46)

The compound was prepared according to general procedure B using 8 (50 mg, 0.16 mmol) and 20 (60 mg, 0.40 mmol). Column chromatography (1 : 20 MeOH/DCM) gave product 46 as an off-white solid (56 mg, 76% yield). ^1^H NMR (400 MHz, DMSO-*d*_6_) *δ* 9.00 (s, 1H), 8.22 (s, 1H), 7.41 (d, *J* = 8.2 Hz, 2H), 7.16 (d, *J* = 8.0 Hz, 2H), 7.14–7.09 (m, 1H), 6.83–6.72 (m, 3H), 5.62 (d, *J* = 7.4 Hz, 1H), 5.27 (d, *J* = 6.9 Hz, 1H), 5.11 (s, 2H), 4.88–4.81 (m, 2H), 4.74 (t, *J* = 5.5 Hz, 1H), 4.09 (dd, *J* = 17.3, 9.9 Hz, 1H), 3.91 (dd, *J* = 6.9, 2.6 Hz, 1H), 3.76 (t, *J* = 6.1 Hz, 1H), 3.50 (t, *J* = 5.9 Hz, 2H), 2.29 (s, 3H). ^13^C NMR (101 MHz, DMSO-*d*_6_) *δ* 147.29, 147.06, 142.79, 136.66, 131.49, 130.74, 130.06, 124.28, 121.92, 119.66, 116.35, 114.70, 89.55, 79.68, 68.09, 67.33, 66.69, 62.61, 60.69, 21.08. HRMS (ESI+) calcd for C_22_H_26_N_3_O_6_S [M + H]^+^: 460.1537, found: 460.1532; HPLC purity: 98.9%.

#### Tolyl 3-(4-((2-(hydroxymethyl)phenyl)methyl)-1,2,3-triazol-1-yl)-3-deoxy-1-thio-β-d-galactopyranoside (47)

The compound was prepared according to general procedure A using 8 (45 mg, 0.14 mmol) and 21 (28 mg, 0.17 mmol). Column chromatography (1 : 20 MeOH/DCM) gave product 47 as an off-white solid (39 mg, 57% yield). ^1^H NMR (400 MHz, DMSO-*d*_6_) *δ* 8.15 (s, 1H), 7.43–7.38 (m, 3H), 7.26–7.20 (m, 1H), 7.19–7.14 (m, 3H), 6.97 (td, *J* = 7.3, 1.0 Hz, 1H), 5.61 (d, *J* = 7.0 Hz, 1H), 5.24 (d, *J* = 7.0 Hz, 1H), 5.15 (s, 2H), 4.97 (t, *J* = 5.7 Hz, 1H), 4.87–4.81 (m, 2H), 4.72 (t, *J* = 5.5 Hz, 1H), 4.49 (d, *J* = 5.6 Hz, 2H), 4.10 (td, *J* = 10.1, 7.2 Hz, 1H), 3.92 (dd, *J* = 6.7, 2.7 Hz, 1H), 3.76 (t, *J* = 6.2 Hz, 1H), 3.50 (t, *J* = 5.8 Hz, 2H), 2.29 (s, 3H); HRMS (ESI+) for C_23_H_27_N_3_O_6_S [M + H]^+^: calculated 474.1693, measured 474.1691; HPLC: purity: 93.0%.

#### Tolyl 3-(4-((3-acetamidophenoxy)methyl)-1,2,3-triazol-1-yl)-3-deoxy-1-thio-β-d-galactopyranoside (48)

The compound was prepared according to general procedure A using 8 (61 mg, 0.20 mmol) and 22 (44 mg, 0.23 mmol). Column chromatography (1 : 20 MeOH/DCM) gave product 48 as an off-white solid (50 mg, 51% yield).^1^H NMR (400 MHz, MeOD) *δ* 8.15 (s, 1H), 7.54–7.44 (m, 2H), 7.37 (t, *J* = 2.2 Hz, 1H), 7.21 (t, *J* = 8.2 Hz, 1H), 7.15 (d, *J* = 7.9 Hz, 2H), 7.06 (ddd, *J* = 8.0, 1.8, 0.8 Hz, 1H), 6.76 (ddd, *J* = 8.2, 2.4, 0.7 Hz, 1H), 5.16 (s, 2H), 4.84 (dd, *J* = 10.6, 2.9 Hz, 1H), 4.75 (d, *J* = 9.5 Hz, 1H), 4.21 (dd, *J* = 10.4, 9.6 Hz, 1H), 4.10 (d, *J* = 2.9 Hz, 1H), 3.82–3.66 (m, 3H), 2.33 (s, 3H), 2.11 (s, 3H). ^13^C NMR (101 MHz, MeOD) *δ* 170.32, 158.75, 142.95, 139.70, 137.38, 131.78, 130.20, 129.25, 123.56, 112.45, 110.01, 106.50, 90.28, 79.52, 68.15, 67.71, 66.55, 61.02, 60.90, 22.48, 19.71. HRMS (ESI+) for C_24_H_29_N_4_O_6_S [M + H]^+^: calculated 501.1802, measured 501.1796; HPLC purity: 98.9%.

#### Tolyl 3-(4-((4-acetamidophenoxy)methyl)-1,2,3-triazol-1-yl)-3-deoxy-1-thio-β-d-galactopyranoside (49)

The compound was prepared according to general procedure A using 8 (49 mg, 0.16 mmol) and 23 (36 mg, 0.19 mmol). Column chromatography (1 : 15 MeOH/DCM) gave product 49 as a yellow solid (34 mg, 43% yield).^1^H NMR (400 MHz, MeOD) *δ* 8.12 (s, 1H), 7.46 (dd, *J* = 18.8, 8.5 Hz, 4H), 7.14 (d, *J* = 7.9 Hz, 2H), 6.96 (d, *J* = 9.0 Hz, 2H), 5.14 (s, 2H), 4.84–4.80 (m, 2H), 4.73 (d, *J* = 9.5 Hz, 1H), 4.18 (t, *J* = 10.0 Hz, 1H), 4.09 (d, *J* = 2.7 Hz, 1H), 3.82–3.65 (m, 3H), 2.32 (s, 3H), 2.09 (s, 3H). ^13^C NMR (101 MHz, CDCl_3_) *δ* 173.95, 159.05, 146.99, 141.33, 135.75, 134.12, 133.18, 127.42, 125.51, 118.51, 94.23, 83.46, 72.09, 71.62, 70.48, 65.24, 64.83, 33.27, 26.08, 23.64. HRMS (ESI+) for C_24_H_28_N_4_O_6_S [M + H]^+^: calculated 501.1802, measured 501.1798; HPLC purity: >99.9%.

#### Tolyl 3-(4-((2-cyclopropanecarboxamidophenyloxy)-methyl)-1,2,3-triazol-1-yl)-3-deoxy-1-thio-β-d-galactopyranoside (50)

The compound was prepared according to general procedure A using 8 (53 mg, 0.17 mmol) and 25 (42 mg, 0.20 mmol). Column chromatography (2 : 1 EtOAc/Hex.) gave product 50 as a white solid (43 mg, 48% yield). ^1^H NMR (400 MHz, CD_3_OD) *δ* 8.17 (s, 1H), 7.92 (d, *J* = 7.6 Hz, 1H), 7.51 (d, *J* = 8.1 Hz, 2H), 7.24–7.07 (m, 4H), 7.01–6.93 (m, 1H), 5.29 (s, 2H), 4.24–4.18 (m, 1H), 4.15–4.09 (m, 1H) 3.84–3.69 (m, 3H) 2.35 (s, 3H), 1.92–1.84 (m, 1H), 0.98–0.91 (m, 2H), 0.88–0.83 (m, 2H); HRMS (ESI+) for C_26_H_30_N_4_O_6_S [M + H]^+^: calculated 527.1959, measured 527.1956; HPLC purity: >99.9%.

#### Tolyl 3-(4-((2-(2-phenyl)acetamidophenyloxy)methyl)-1,2,3-triazol-1-yl)-3-deoxy-1-thio-β-d-galactopyranoside (51)

The compound was prepared according to general procedure A using 8 (72 mg, 0.23 mmol) and 27 (71 mg, 0.27 mmol). Column chromatography (4 : 1 EtOAc/Hex.) gave product 51 as a white solid (64 mg, 48% yield). ^1^H NMR (400 MHz, DMSO-*d*_6_) *δ* 9.16 (s, 1H), 8.20 (s, 1H), 7.89 (d, *J* = 8.0 Hz, 1H), 7.42 (d, *J* = 8.1 Hz, 2H), 7.35–7.28 (m, 5H), 7.26–7.20 (m, 1H), 7.16 (d, *J* = 8.0 Hz, 2H), 7.08 (td, *J* = 7.9, 1.0 Hz, 1H), 6.95–6.90 (m, 1H), 5.59 (d, *J* = 7.5 Hz, 1H), 5.25–5.20 (m, 3H), 4.90–4.84 (m, 2H), 4.74 (t, *J* = 5.5 Hz, 1H), 4.12 (td, *J* = 9.8, 7.6 Hz, 1H), 3.97–3.92 (m, 1H), 3.78 (t, *J* = 6.3 Hz, 1H), 3.72 (s, 2H), 3.52 (t, *J* = 5.9 Hz, 2H), 2.29 (s, 3H); HRMS (ESI+) for C_30_H_32_N_4_O_6_S [M + H]^+^: calculated 577.2115, measured 577.2111; HPLC purity: >99.9%.

#### Tolyl 3-(4-((4-acetamido-2-nitrophenyloxy)methyl)-1,2,3-triazol-1-yl)-3-deoxy-1-thio-β-d-galactopyranoside (52)

The compound was prepared according to general procedure A using 8 (48 mg, 0.15 mmol) and 30 (43 mg, 0.18 mmol). Column chromatography (1 : 15 MeOH/DCM) gave product 52 as a white solid (56 mg, 66% yield). ^1^H NMR (400 MHz, DMSO-*d*_6_) *δ* 9.48 (s, 1H), 8.95 (d, *J* = 2.5 Hz, 1H), 8.28 (s, 1H), 8.02 (dd, *J* = 9.1, 2.9 Hz, 1H), 7.56 (d, *J* = 9.2 Hz, 1H), 7.41 (d, *J* = 8.1 Hz, 2H), 7.16 (d, *J* = 8.0 Hz, 2H), 5.58 (d, *J* = 7.4 Hz, 1H), 5.46 (s, 2H), 5.22 (d, *J* = 6.8 Hz, 1H), 4.89–4.81 (m, 2H), 4.73 (t, *J* = 5.6 Hz, 1H), 4.09 (td, *J* = 9.8, 7.5 Hz, 1H), 3.91 (dd, *J* = 6.8, 2.9 Hz, 1H), 3.77 (t, *J* = 6.2 Hz, 1H), 3.51 (t, *J* = 5.8 Hz, 2H), 2.30 (s, 3H), 2.15 (s, 3H); HRMS (ESI+) for C_24_H_27_N_5_O_8_S [M + H]^+^: calculated 546.1653, measured 546.1651; HPLC purity: >99.9%.

#### Tolyl 3-(4-((2,5-diacetamidophenyloxy)methyl)-1,2,3-triazol-1-yl)-3-deoxy-1-thio-β-d-galactopyranoside (53)

The compound was prepared according to general procedure A using 8 (60 mg, 0.19 mmol) and 29 (50 mg, 0.20 mmol). Column chromatography (1 : 10 MeOH/DCM) gave product 53 as a white solid (82 mg, 76% yield). ^1^H NMR (400 MHz, DMSO-*d*_6_) *δ* 9.83 (s, 1H), 9.05 (s, 1H), 8.20 (s, 1H), 8.02 (d, *J* = 1.5 Hz, 1H), 7.47–7.37 (m, 3H), 7.17 (t, *J* = 9.1 Hz, 3H), 5.58 (s, 1H), 5.28–5.09 (m, 3H), 4.89–4.78 (m, 2H), 4.73 (s, 1H), 4.08 (t, *J* = 10.0 Hz, 1H), 3.91 (s, 1H), 3.76 (t, *J* = 6.3 Hz, 1H), 3.50 (d, *J* = 3.4 Hz, 2H), 2.29 (s, 3H), 2.07 (s, 3H), 1.99 (s, 3H). ^13^C NMR (101 MHz, DMSO-*d*_6_) *δ* 168.81, 168.31, 145.09, 142.65, 136.67, 133.29, 131.44, 130.79, 130.05, 128.31, 124.33, 115.59, 114.42, 114.19, 89.51, 79.68, 68.07, 67.40, 66.67, 63.47, 60.67, 24.29, 21.07. HRMS (ESI+) calcd for C_26_H_32_O_7_N_5_S [M + H]^+^: 558.2017, found: 558.2013. HPLC purity: >99.9%.

#### Tolyl 3-(4-((quinolin-8-oxy)methyl)-1,2,3-triazol-1-yl)-3-deoxy-1-thio-β-d-galactopyranoside (54)

The compound was prepared according to general procedure B using 8 (64 mg, 0.21 mmol) and 31 (50 mg, 0.27 mmol). Column chromatography (1 : 20 MeOH/DCM) gave product 54 as a light brown solid (44 mg, 43% yield). ^1^H NMR (400 MHz, DMSO-*d*_6_) *δ* 8.83 (d, *J* = 2.6 Hz, 1H), 8.33 (dd, *J* = 8.3, 1.5 Hz, 1H), 8.27 (s, 1H), 7.55 (q, *J* = 4.2 Hz, 3H), 7.48–7.40 (m, 3H), 7.17 (d, *J* = 8.0 Hz, 2H), 5.68 (d, *J* = 7.3 Hz, 1H), 5.34 (d, *J* = 5.3 Hz, 3H), 4.91 (dd, *J* = 10.5, 2.8 Hz, 1H), 4.86 (d, *J* = 9.4 Hz, 1H), 4.77 (t, *J* = 5.0 Hz, 1H), 4.12 (dd, *J* = 17.4, 9.8 Hz, 1H), 3.95 (dd, *J* = 6.6, 2.5 Hz, 1H), 3.79 (t, *J* = 6.1 Hz, 1H), 3.52 (t, *J* = 5.3 Hz, 2H), 2.30 (s, 3H). ^13^C NMR (101 MHz, DMSO-*d*_6_) *δ* 154.48, 149.42, 142.23, 140.11, 136.67, 136.30, 131.47, 130.79, 130.05, 129.53, 127.30, 124.71, 122.36, 120.39, 110.24, 89.55, 79.71, 68.11, 67.39, 66.69, 62.29, 60.71, 21.08. HRMS (ESI+) calcd for C_25_H_26_O_5_N_4_S [M + H]^+^: 495.1697, found: 495.1681. HPLC purity: >96.8%.

#### Tolyl 3-(4-((2-methoxycarbonylquinolin-8-oxy)methyl)-1,2,3-triazol-1-yl)-3-deoxy-1-thio-β-d-galactopyranoside (55)

The compound was prepared according to general procedure B using 8 (80 mg, 0.26 mmol) and 32 (70 mg, 0.29 mmol). Column chromatography (1 : 20 MeOH/DCM) gave product 55 as a white solid (139 mg, 98% yield). ^1^H NMR (400 MHz, DMSO-*d*_6_) *δ* 8.52 (d, *J* = 8.6 Hz, 1H), 8.32 (s, 1H), 8.12 (d, *J* = 8.5 Hz, 1H), 7.72–7.56 (m, 3H), 7.43–7.38 (m, 2H), 7.15 (d, *J* = 8.0 Hz, 2H), 5.65 (d, *J* = 7.3 Hz, 1H), 5.42 (s, 2H), 5.34 (d, *J* = 6.9 Hz, 1H), 4.89 (dd, *J* = 10.5, 2.9 Hz, 1H), 4.84 (d, *J* = 9.5 Hz, 1H), 4.72 (t, *J* = 5.6 Hz, 1H), 4.14–4.05 (m, 1H), 3.96–3.90 (m, 4H), 3.77 (t, *J* = 6.3 Hz, 1H), 3.50 (t, *J* = 5.9 Hz, 2H), 2.29 (s, 3H). ^13^C NMR (101 MHz, DMSO-*d*_6_) *δ* 165.91, 155.07, 146.73, 142.02, 139.43, 137.94, 136.65, 131.46, 130.79, 130.65, 130.04, 129.76, 125.07, 121.78, 120.18, 111.34, 89.52, 79.71, 68.02, 67.41, 66.71, 62.35, 60.72, 53.06, 46.06, 21.07. HRMS (ESI+) calcd for C_27_H_29_N_4_O_7_S [M + H]^+^: 553.1751, found: 553.1747; HPLC purity: 95.6%.

#### Tolyl 3-(4-((2-carboxyquinolin-8-oxy)methyl)-1,2,3-triazol-1-yl)-3-deoxy-1-thio-β-d-galactopyranoside (56)

Compound 55 (60 mg, 0.11 mmol) was dissolved in acetonitrile (4 mL). NaOH_aq_ (1.0 M, 4 mL) was added and the mixture was heated to 50 °C for 1 h. At r. t., HCl_aq_ (2.0 M, 4 mL) was added. The product was filtered off as a precipitate and dried *in vacuo* to give 56 as a white solid (48 mg, 82% yield). ^1^H NMR (400 MHz, DMSO-*d*_6_) *δ* 8.50 (d, *J* = 8.4 Hz, 1H), 8.33 (s, 1H), 8.11 (d, *J* = 8.4 Hz, 1H), 7.76–7.52 (m, 3H), 7.41 (d, *J* = 7.8 Hz, 2H), 7.16 (d, *J* = 7.8 Hz, 2H), 5.68 (d, *J* = 6.7 Hz, 1H), 5.48–5.31 (m, 3H), 4.94–4.80 (m, 2H), 4.77–4.69 (m, 1H), 4.10 (dd, *J* = 16.9, 9.5 Hz, 1H), 3.94 (d, *J* = 3.5 Hz, 1H), 3.83–3.74 (m, 1H), 2.29 (s, 3H). HRMS (ESI+) calcd for C_26_H_27_N_4_O_7_S [M + H]^+^: 539.1595, found: 539.1593; HPLC purity: 97.3%.

#### 3,4-Dichlorophenyl 2,4,6-tri-*O*-acetyl-3-(4-((2-acetamidophenoxy)methyl)-1,2,3-triazol-1-yl)-3-deoxy-1-thio-α-d-galactopyranoside (58)

The compound was prepared according to general procedure B using 57 (40 mg, 0.08 mmol) and 12 (23 mg, 0.12 mmol). Column chromatography (1 : 2 EtOAc/Hept.) gave 58 as a colourless solid (48 mg, 87% yield). ^1^H NMR (400 MHz, CDCl_3_) *δ* 8.40–8.30 (m, 1H), 7.59 (d, *J* = 2.1 Hz, 1H), 7.40 (d, *J* = 8.4 Hz, 1H), 7.31 (dd, *J* = 8.4, 2.1 Hz, 1H), 7.08–6.92 (m), 6.08 (d, *J* = 5.5 Hz, 1H), 6.02 (dd, *J* = 11.5, 5.6 Hz, 1H), 5.54 (d, *J* = 2.1 Hz, 1H), 5.24 (s, 2H), 5.20 (dd, *J* = 11.3, 2.1 Hz, 1H), 4.83–4.77 (m, 1H), 4.76 (d, *J* = 2.4 Hz, 1H), 4.16–4.08 (m, 1H), 4.04 (dd, *J* = 11.6, 7.4 Hz, 1H), 2.17 (s, 3H), 2.00–1.95 (m, 3H), 1.93 (s, 3H), 1.91 (s, 3H).

#### 3,4-Dichlorophenyl 3-(4-((2-acetamidophenoxy)methyl)-1,2,3-triazol-1-yl)-3-deoxy-1-thio-α-d-galactopyranoside (59)

Compound 58 (48 mg, 0.050 mmol) was suspended in MeOH (4 mL) and sodium methoxide (65 mg, 1.21 mmol) was added and the mixture stirred at room temperature for 1 h. Amberlite® IRC120 H was added, and the mixture stirred for another 10 minutes before filtering and removing the solvents *in vacuo*. Preparative column chromatography, followed by lyophilisation from acetonitrile/H_2_O gave 59 as a white solid (22 mg, 48% yield). ^1^H NMR (400 MHz, MeOD) *δ* 8.20 (s, 1H), 7.88 (dd, *J* = 8.0, 1.4 Hz, 1H), 7.79 (d, *J* = 2.0 Hz, 1H), 7.53 (dd, *J* = 8.4, 2.1 Hz, 1H), 7.47 (d, *J* = 8.4 Hz, 1H), 7.20 (dd, *J* = 8.2, 0.8 Hz, 1H), 7.12 (td, *J* = 8.0, 1.5 Hz, 1H), 6.96 (td, *J* = 7.9, 1.1 Hz, 1H), 5.82 (d, *J* = 5.3 Hz, 1H), 5.27 (s, 2H), 4.96 (dd, *J* = 11.4, 2.8 Hz, 1H), 4.85–4.81 (m, 1H), 4.47 (t, *J* = 6.2 Hz, 1H), 4.17 (d, *J* = 2.0 Hz, 1H), 3.75–3.64 (m, 2H), 2.14 (s, 3H). HRMS (ESI+) calcd for C_23_H_25_N_4_O_6_SCl_2_ [M + H]^+^: 555.0872, found: 555.0880; HPLC purity: 98.6%.

#### Di-(2,4,6-tri-*O*-acetyl-3-(4-((2-acetamidophenyloxy)methyl)-1,2,3-triazol-1-yl)-3-deoxy-β-d-galactopyranosyl) sulfane (61)

The compound was prepared according to general procedure B using 60 (ref. 45) (50 mg, 0.08 mmol) and 12 (43 mg, 0.227 mmol). Column chromatography (1 : 1 EtOAc/Hept.) gave 61 as a colourless oil (64 mg, 81% yield). ^1^H NMR (400 MHz, DMSO-*d*_6_) *δ* 8.33 (dd, *J* = 7.6, 2.0 Hz, 2H), 7.88 (s, 2H), 7.65 (s, 2H), 7.05–6.91 (m, 6H), 5.67 (dd, *J* = 10.8, 10.0 Hz, 2H), 5.55 (d, *J* = 3.2 Hz, 2H), 5.28–5.14 (m, 6H), 4.99 (d, *J* = 9.8 Hz, 2H), 4.27–4.17 (m, 2H), 4.16–4.05 (m, 4H), 2.19 (s, 6H), 2.06 (s, 6H), 1.89 (s, 6H), 1.86 (s, 6H).

#### Di-(3-(4-((2-acetamidophenyloxy)methyl)-1,2,3-triazol-1-yl)-3-deoxy-β-d-galactopyranosyl) sulfane (62)

Compound 61 (60 mg, 0.06 mmol) was suspended in MeOH (5 mL). Sodium methoxide (50 mg, 0.926 mmol) was added and the mixture stirred at room temperature for 1 h. Amberlite® IRC120 H was added, and the mixture stirred for another 10 minutes before filtering and removing the solvents *in vacuo.* Preparative column chromatography, followed by lyophilisation from acetonitrile/H_2_O gave 62 as a white solid (22 mg, 48% yield). ^1^H NMR (400 MHz, MeOD) *δ* 8.24 (s, 2H), 7.86 (dd, *J* = 8.0, 1.5 Hz, 2H), 7.17 (dd, *J* = 8.3, 1.2 Hz, 2H), 7.15–7.07 (m, 2H), 6.94 (td, *J* = 7.9, 1.4 Hz, 2H), 5.25 (s, 4H), 4.58 (t, *J* = 10.1 Hz, 2H), 4.10 (d, *J* = 2.6 Hz, 2H), 3.87–3.73 (m, 4H), 3.66 (dd, *J* = 11.4, 4.5 Hz, 2H), 2.12 (s, 6H). HRMS (ESI+) calcd for C_34_H_43_N_8_O_12_S [M + H]^+^: 787.2721, found: 787.2734; HPLC purity: 99.8%.

### 4.2 Molecular docking

A library of ligand conformers was generated with OMEGA (OMEGA version 4.1.1.1 OpenEye Scientific Software, Santa Fe, NM; https://www.eyesopen.com),^46^ with a maximum number of 200 conformations set as default. Ligand docking was performed using galectin-1 (PDB code: 4Y24),^39^ galectin-3 (PDB code: 5H9P)^39^ and galectin-8N (PDB code: 7AEN)^47^ crystal structures. Docking was done inside a grid box surrounding the ligand with the volume of 8795 Å^3^ for galectin-1, 8891 Å^3^ for galectin-3 and 6001 Å^3^ for galectin-8N, and outer contour of 4173 Å^3^ for galectin-1, 4522 Å^3^ for galectin-3 and 1204 Å^3^ for galectin-8N using Make Receptor 4.0.1.

HYBRID (OEDocking version 4.1.0.1. OpenEye Scientific Software, Santa Fe, NM. https://www.eyesopen.com)^48,49^ was used for ligand docking with the default settings. The ten highest ranked docking poses per ligand were evaluated using Chemgauss4 score and relative position to the native ligand.

### 4.3 Molecular dynamics simulations

The molecular dynamics software NAMD (version 3.0)^50^ and CHARMM36m force field^51,52^ were used for MD simulations using the docking complexes of galectin-1, galectin-3 and galectin-8N with 59 and 62 as input structures. Molecular mechanics parameters for ligands were estimated using ParamChem tool.^53–55^ Steepest descent (10 000 steps) and adopted basis Newton–Raphson (10 000 steps) energy minimizations were performed to remove atomic clashes and optimize the atomic coordinates of the complexes. The structure of the energy minimized complex for MD simulation was prepared using psfgen in VMD (version 1.9.1.).^56^ The complex was embedded in box of TIP3P water molecules. The system was neutralized by addition of NaCl. The MD simulation was carried out in the NPT ensemble employing periodic boundary conditions. Langevin dynamics and Langevin piston methods were used for temperature (300 K) and pressure (1 atm) control, respectively. Short- and long-range forces were calculated every 1 and 2 time steps, respectively, with a time step of 2.0 ps. The smooth particle mesh Ewald method^57^ was used to calculate electrostatic interactions. The short-range interactions were cut off at 12 Å. All chemical bonds between hydrogen and heavy atoms were held fixed using the SHAKE algorithm.^58^ The simulation consisted of three consecutive steps: (i) solvent equilibration for 0.5 ns with ligand and protein constrained harmonically around the initial structure, (ii) equilibration of the complete system for 0.5 ns with ligand and protein released, and (iii) an unconstrained 200 ns production run. For structure-based pharmacophore modelling 1000 frames from the production run were saved separately and used for interaction analysis.

### 4.4 Structure-based pharmacophore modelling

The MD trajectory of galectin in complex with the ligand was used for chemical feature interaction analysis using LigandScout 4.4 Expert.^44^ An ensemble of structure-based pharmacophore models was generated from each protein-ligand trajectory. LigandScout 4.4 Expert was used to generate 1000 structure-based pharmacophore models from the 200 ns MD simulation. The graphical representations of the calculated binding poses were obtained using VIDA (VIDA version 5.0.0.1. OpenEye Scientific Software, Santa Fe, NM. https://www.eyesopen.com).

### 4.5 Competitive fluorescence polarisation experiments

Human galectins -1, -3, and -8N were expressed and purified as previously described. Fluorescence polarisation experiments were performed using the PHERAstar FS plate reader (software version 2.10 R3), and the fluorescence anisotropy of fluorescein tagged probes was measured by excitation at 485 nm and emission at 520 nm. The specific conditions for galectins -1, -3, and -8N were kept as reported.^12,37,38^ The synthesised compounds were dissolved in pure DMSO at 20 or 40 mM concentration and diluted in PBS to 3–6 different concentrations, and each concentration was tested in duplicate. The highest inhibitor concentrations tested were 1.2 or 1.5 mM. The average values of *K*_d_ and SEM were calculated from 4 to 8 duplicate measurements, showing 10–90% inhibition.

## Conflicts of interest

H. L. and U. J. N. are shareholders in Galecto Biotech AB, a company developing galectin inhibitors. The other authors have no conflicts to declare.

## Supplementary Material

RA-012-D2RA03163A-s001
