# Supplementary material for: Design and synthesis of novel 3-triazolyl-1-thiogalactosides as galectin-1, -3 and -8 inhibitors
Source: RSC Adv. 2022 Jun 30;12(29):18973–84. doi: 10.1039/d2ra03163a (PMC9245910; doi:10.1039/d2ra03163a)

Electronic Supplementary Material (ESI) for RSC Advances.
This journal is © The Royal Society of Chemistry 2022

Supporting information for

Design and synthesis of novel 3-triazolylgalactosides as galectin-1, -3 and -8 inhibitors

Sjors van Klaveren, Jaka Dernovšek, Žiga Jakopin, Marko Anderluh, Hakon Leffler, Ulf J. Nilsson and Tihomir Tomašič

2-(Propargyloxy)nitrobenzene (9)

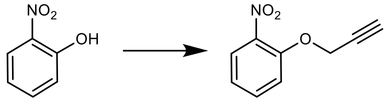

2-Nitrophenol (500 mg, 3.59 mmol) and K_2CO_3 (1 g, 7.24 mmol) were suspended in acetone (15 mL). Propargyl bromide (0.5 mL, 80 wt% in toluene 4.49 mmol) was added and the reaction was refluxed overnight. Solvent was removed *in vacuo* and water was added to precipitate the product. Solids were filtered and dried *in vacuo* to give intermediate **9** (570 mg, 90% yield). ^{1}H NMR (400 MHz, $CDCl_3$) δ 7.86 (dd, *J* = 8.1, 1.7 Hz, 2H), 7.56 (ddd, *J* = 8.5, 7.5, 1.7 Hz, 2H), 7.29 – 7.23 (m, 3H), 7.10 (ddd, *J* = 8.5, 7.6, 1.1 Hz, 2H), 4.86 (d, *J* = 2.4 Hz, 4H), 2.58 (t, *J* = 2.4 Hz, 2H).

2-(Propargyloxy)aniline (10)

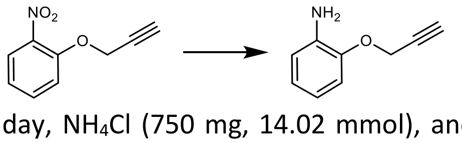

9 (500 mg, 2.82 mmol), NH_4Cl (750 mg, 14.02 mmol), and iron powder (475 mg, 8.51 mmol) were suspended in ethanol (10 mL). The reaction was heated to reflux overnight. The following day, NH_4Cl (750 mg, 14.02 mmol), and iron powder (475 mg, 8.51 mmol) were added to the reaction mixture and the reaction refluxed overnight. The reaction mixture was filtered, and the solvent was removed from the filtrate *in vacuo*. The mixture was extracted (EtOAc/ 0.5 M HCl_{aq}), aqueous layers were basified to pH 11, extracted with EtOAc. Organic layers were combined, dried over Na_2SO_4, filtered, and concentrated *in vacuo* to give intermediate **9** as a light brown oil (313 mg, 75% yield).

^{1}H NMR (400 MHz, $CDCl_3$) δ 6.92 (dd, *J* = 7.8, 1.1 Hz, 1H), 6.88 – 6.80 (m, 1H), 6.77 – 6.69 (m, 2H), 4.72 (d, *J* = 2.4 Hz, 2H), 3.81 (s, 2H), 2.52 (t, *J* = 2.4 Hz, 1H).

N-Methyl 2-(propargyloxy)aniline (11)

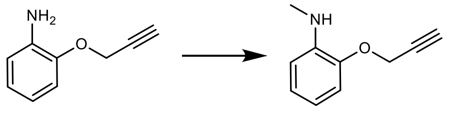

10 (190 mg, 1.29 mmol), and NaH (62 mg, 1.55 mmol) were suspended in dry DMF (1.5 mL) and MeI (81 µL, 1.30 mmol) was added slowly over 30 minutes at 50 °C. The mixture was heated to 50 °C for another 5 h. Solvent was removed *in vacuo*. The product was extracted (EtOAc/H_2O), organic layers were combined dried over Na_2SO_4, filtered, and concentrated *in vacuo*. Column chromatography (1:9 EtOAc/Hex.) gave intermediate **11** as a slightly yellow solid (57 mg, 27% yield).

^{1}H NMR (400 MHz, $CDCl_3$) δ 6.95 (td, *J* = 7.7, 1.3 Hz, 1H), 6.88 (dd, *J* = 7.9, 1.3 Hz, 1H), 6.66 (td, *J* = 7.7, 1.6 Hz, 1H), 6.63 (dd, *J* = 7.9, 1.5 Hz, 1H), 4.71 (d, *J* = 2.4 Hz, 2H), 4.26 (s, 1H), 2.86 (s, 3H), 2.52 (t, *J* = 2.4 Hz, 1H).

N-(2-(Propargyloxy)phenyl)acetamide (12)

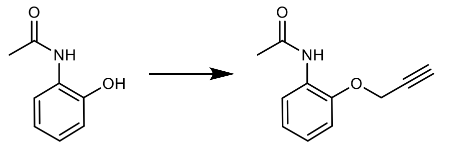

2-Acetamidophenol (500 mg, 3.33 mmol) and K_2CO_3 (1.84 g, 13.31 mmol) were suspended in acetone (15 mL). Propargyl bromide (1.11 mL, 80% wt in toluene, 9.97 mmol) was added and the reaction was refluxed overnight. Solvent was removed *in vacuo*. The product was extracted (EtOAc/H_2O), organic layers were combined, dried over Na_2SO_4, filtered, and concentrated *in vacuo*. Crystallisation from Et_2O-Heptane gave intermediate **12** as long colourless needles (430 mg, 69% yield).

^{1}H NMR (400 MHz, $CDCl_3$) δ 8.37 (dd, *J* = 7.4, 1.7 Hz, 1H), 7.74 (s, 1H), 7.09 – 6.94 (m, 3H), 4.76 (d, *J* = 2.4 Hz, 2H), 2.56 (t, *J* = 2.4 Hz, 1H), 2.20 (s, 3H).

N-(2-(Propargyloxy)phenyl)methanesulfonamide (13)

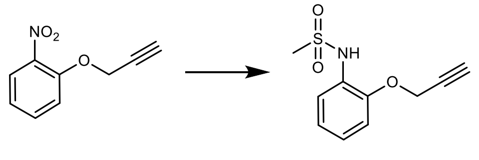

9 (404 mg, 2.28 mmol) and iron powder (1.27 g, 22.8 mmol) were suspended in acetic acid (15 mL). The reaction was stirred at r.t. overnight. Afterwards, methanesulfonyl chloride (200 µL, 2.58 mmol) and $NaHCO_3$ (506 mg, 5.75 mmol) were added and the reaction stirred at r.t. overnight. Solids were removed by filtration over Celite® and solvent was removed *in vacuo*. Column chromatography (2:1 EtOAc/Hex.) gave intermediate **13** as a yellow amorphous solid (67 mg, 13 % yield).

^{1}H NMR (400 MHz, $CDCl_3$) δ 8.41 – 8.35 (m, 1H), 7.73 (s, 1H), 7.06 – 6.97 (m, 3H), 4.77 (d, *J* = 2.4 Hz, 2H), 2.56 (m, 1H), 2.21 (s, 3H).

Methyl 3-(propargyloxy)benzoate (14)

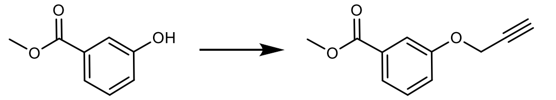

Methyl 3-hydroxybenzoate (80 mg, 0.53 mmol) and K_2CO_3 (290 mg, 2.10 mmol) were suspended in acetone (8 mL). Propargyl bromide (0.20 mL, 80 wt% in toluene 1.80 mmol) was added and the reaction was refluxed overnight. Solvent was removed *in vacuo*. The product was extracted (EtOAc/H_2O), organic layers were combined, dried over Na_2SO_4, filtered, and concentrated *in vacuo*. Column chromatography (1:6 EtOAc/Hex.) gave product **14** as a slightly yellow solid (92 mg, 92% yield).

^{1}H NMR (400 MHz, $CDCl_3$) δ 7.73 – 7.66 (m, 1H), 7.63 (dd, *J* = 2.6, 1.5 Hz, 1H), 7.41 – 7.33 (m, 1H), 7.18 (ddd, *J* = 8.3, 2.7, 0.9 Hz, 1H), 4.74 (d, *J* = 2.4 Hz, 2H), 3.92 (s, 3H), 2.54 (t, *J* = 2.4 Hz, 1H).

Methyl 4-nitro-3-(propargyloxy)benzoate (15)

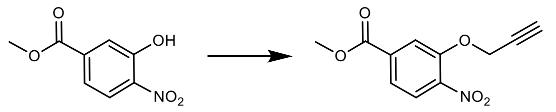

Methyl 3-hydroxy-4-nitrobenzoate (400 mg, 2.03 mmol) and K_2CO_3 (1.12 mg, 8.12 mmol) were suspended in acetone (10 mL). Propargyl bromide (0.68mL, 80 wt% in toluene, 6.10 mmol) was added and the reaction was refluxed overnight. Solvent was removed *in vacuo*. The product was extracted (EtOAc/ 0.1 M $NaOH_{aq}$), organic layers were combined dried over Na_2SO_4, filtered, and concentrated *in vacuo*. Crystallisation from EtOAc/heptane gave product **15** as a slightly yellow solid (350 mg, 73% yield).

^{1}H NMR (400 MHz, $CDCl_3$) δ 7.91 (d, *J* = 1.5 Hz, 1H), 7.86 (d, *J* = 8.4 Hz, 1H), 7.76 (dd, *J* = 8.4, 1.6 Hz, 1H), 4.91 (d, *J* = 2.4 Hz, 2H), 3.98 (s, 3H), 2.61 (t, *J* = 2.4 Hz, 1H).

Methyl 4-amino-3-(propargyloxy)benzoate (16)

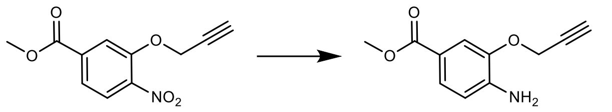

15 (80 mg, 0.34 mmol), NH_4Cl (90 mg, 1.68 mmol), and iron powder (57 mg, 1.02 mmol) were suspended in ethanol (6 mL). The reaction was heated for 4 h. Solvent was removed *in vacuo*, and the product was extracted (EtOAc/ 0.5 M HCl_{aq}), organic layers were combined, dried over Na_2SO_4, filtered, and concentrated *in vacuo* to give intermediate **16** as a light brown oil (67 mg, 96 % yield).

^1H NMR (400 MHz, $CDCl_3$) δ 7.58 (dd, *J* = 8.1, 1.8 Hz, 1H), 7.55 (d, *J* = 1.7 Hz, 1H), 6.68 (d, *J* = 8.2 Hz, 1H), 4.76 (d, *J* = 2.4 Hz, 2H), 4.27 (s, 2H), 3.86 (s, 3H), 2.54 (t, *J* = 2.4 Hz, 1H).

Methyl 3-hydroxy-4-acetamidobenzoate (17)

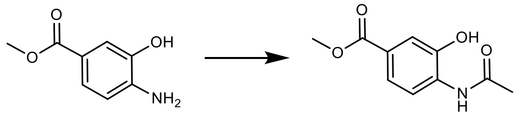

Methyl 3-hydroxy-4-aminobenzoate (500 mg, 2.99 mmol) was dissolved in water (10 mL). The mixture was cooled in an ice bath, acetic anhydride (320 µL, 3.39 mmol) was added dropwise, and the mixture allowed to come to room temperature over 2 h. The product was filtered off as a precipitate and dried *in vacuo* to give intermediate **17** as a brown solid (457 mg, 73% yield).

^1H NMR (400 MHz, DMSO-d_6) δ 10.31 (s, 1H), 9.34 (s, 1H), 8.09 (d, *J* = 8.4 Hz, 1H), 7.45 (d, *J* = 1.9 Hz, 1H), 7.40 (dd, *J* = 8.4 Hz, 1.9 Hz, 1H), 3.80 (s, 3H), 2.13 (s, 3H).

Methyl 3-(propargyloxy)-4-acetamidobenzoate (18)

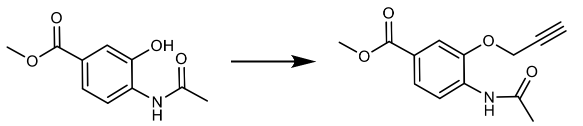

17 (392 mg, 1.87 mmol) and K_2CO_3 (609 mg, 4.40 mmol) were suspended in acetone (15 mL). Propargyl bromide (260 mL, 80 wt% in toluene 2.33 mmol) was added and the reaction was refluxed overnight. Solvent was removed *in vacuo* and column chromatography (1:2 EtOAc/Hex.) gave intermediate **18** as a brown solid (260 mg, 56% yield).

^1H NMR (400 MHz, DMSO-d_6) δ 9.40 (s, 1H), 8.23 (d, *J* = 8.5 Hz, 1H), 7.67 (d, *J* = 1.8 Hz, 1H), 7.60 (dd, *J* = 8.5, 1.8 Hz, 1H), 4.97 (d, *J* = 2.4 Hz, 2H), 3.84 (s, 3H), 3.66 (t, *J* = 2.3 Hz, 1H), 2.14 (s, 3H).

(Propargyloxy)benzene (19)

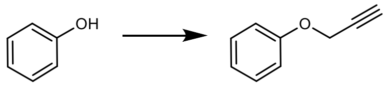

Phenol (400 mg, 4.25 mmol) and K_2CO_3 (1.17 g, 8.50 mmol) were suspended in acetonitrile (5 mL). Propargyl bromide (0.568 mL, 80 wt% in toluene, 5.10 mmol) was added and the reaction was refluxed overnight. Solvent was removed *in vacuo*, and the product was extracted (EtOAc/H_2O), organic layers were combined, dried over Na_2SO_4, filtered, and concentrated *in vacuo* to give intermediate **19** as a yellow oil (429 mg, 76% yield).

^1H NMR (400 MHz, $CDCl_3$) δ 7.35 – 7.28 (m, 2H), 7.03 – 6.96 (m, 3H), 4.70 (d, *J* = 2.4 Hz, 2H), 2.52 (t, *J* = 2.4 Hz, 1H).

2-(Propargyloxy)phenol (20)

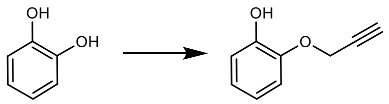

Catechol (80 mg, 0.73 mmol) and Na_2CO_3 (240 mg, 2.26 mmol) were suspended in acetone (8 mL). Propargyl bromide (0.160 mL, 80 wt% in toluene, 1.44 mmol) was added and the reaction was refluxed

overnight. Solvent was removed *in vacuo*, and the product was extracted (EtOAc/ 1 M HCl_{aq}), organic layers were combined, dried over Na_2SO_4, filtered, and concentrated *in vacuo*. Column chromatography (1:4 EtOAc/Hex.) gave intermediate **20** as a clear oil (68 mg, 63% yield).

^{1}H NMR (400 MHz, $CDCl_3$) δ 6.98 – 6.88 (m, 2H), 6.86 – 6.82 (m, 1H), 6.82 – 6.74 (m, 1H), 4.72 (d, *J* = 2.4 Hz, 2H), 2.52 (t, *J* = 2.4 Hz, 1H).

2-((Propargyloxy)phenyl)methanol (21)

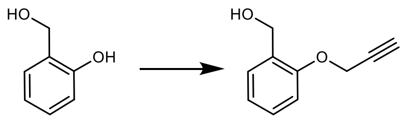

2-(Hydroxymethyl)phenol (200 mg, 1.61 mmol) and K_2CO_3 (520 mg, 3.76 mmol) were suspended in acetone (10 mL). Propargyl bromide (0.225 mL, 80 wt% in toluene, 2.02 mmol) was added and the reaction was refluxed overnight. Solvent was removed *in vacuo*, and the product was extracted (EtOAc/ 1 M HCl_{aq}), organic layers were combined, dried over Na_2SO_4, filtered, and concentrated *in vacuo* to give intermediate **21** as an orange oil (141 mg, 54% yield).

^{1}H NMR (400 MHz, $CDCl_3$) δ 7.37 – 7.26 (m, 2H), 7.04 – 6.97 (m, 2H), 4.76 (d, *J* = 2.4 Hz, 2H), 4.71 (d, *J* = 6.1 Hz, 1H), 2.52 (t, *J* = 2.4 Hz, 1H), 2.18 (m, 1H).

***N*-(3-(Propargyloxy)phenyl)acetamide (22)**

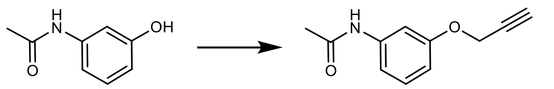

N-(3-Hydroxyphenyl)acetamide (200 mg, 1.32 mmol) and K_2CO_3 (430 mg, 3.11 mmol) were suspended in acetone (10 mL). Propargyl bromide (0.185 mL, 80 wt% in toluene, 1.66 mmol) was added and the reaction was refluxed overnight. Solvent was removed *in vacuo*, and the product was extracted (EtOAc/ 1 M $NaOH_{aq}$), organic layers were combined, dried over Na_2SO_4, filtered, and concentrated *in vacuo* to give intermediate **22** as a light yellow solid (165 mg, 66% yield).

^{1}H NMR (400 MHz, CDCl3) δ 7.33 (s, 1H), 7.23 (t, *J* = 8.2 Hz, 1H), 7.13 (s, 1H), 7.03 (dd, *J* = 8.1, 0.6 Hz, 1H), 6.74 (dd, *J* = 8.1, 1.8 Hz, 1H), 4.69 (d, *J* = 2.4 Hz, 2H), 2.52 (t, *J* = 2.4 Hz, 1H), 2.18 (s, 3H).

***N*-(4-(Propargyloxy)phenyl)acetamide (23)**

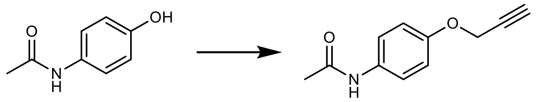

N-(4-Hydroxyphenyl)acetamide (220 mg, 1.46 mmol) and K_2CO_3 (455 mg, 3.29 mmol) were suspended in acetone (10 mL). Propargyl bromide (0.20 mL, 80 wt% in toluene, 1.80 mmol) was added and the reaction was refluxed overnight. Solvent was removed *in vacuo*, and the product was extracted (EtOAc/ 1 M $NaOH_{aq}$), organic layers were combined, dried over Na_2SO_4, filtered, and concentrated *in vacuo* to give intermediate **23** as a white solid (212 mg, 77% yield).

^{1}H NMR (400 MHz, $CDCl_3$) δ 7.44 – 7.39 (m, 2H), 7.13 (s, 1H), 6.96 – 6.92 (m, 2H), 4.67 (d, *J* = 2.4 Hz, 2H), 2.51 (t, *J* = 2.4 Hz, 1H), 2.16 (s, 3H).

***N*-(2-Hydroxyphenyl)cyclopropanecarboxamide (24)**

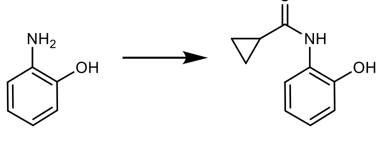

2-Aminphenol (315 mg, 2.89 mmol) was dissolved in ethyl acetate (25 mL). On an ice bath, triethylamine (500 µL, 3.61 mmol) and cyclopropanecarbonyl chloride (278 µL, 3.06 mmol) were added and the mixture stirred for 5 h. The precipitate was filtered off, and the filtrate was extracted (EtOAc/1 M $NaOH_{aq}$), aqueous layers were combined, acidified to pH 3 using 1 M HCl_{aq}, and the product was extracted (EtOAc), organic layers were combined, dried over Na_2SO_4, filtered, and concentrated *in vacuo* to give intermediate **24** as a yellow solid (404 mg, 79 % yield).

^{1}H NMR (400 MHz, $CDCl_3$) δ 8.99 (s, 1H), 7.66 (s, 1H), 7.16 – 7.07 (m, 1H), 7.04 – 6.95 (m, 2H), 6.89 – 6.81 (m, 1H), 1.21 – 1.12 (m, 2H), 1.01 – 0.89 (m, 2H).

***N*-(2-(Propargyloxy)phenyl)cyclopropanecarboxamide (25)**

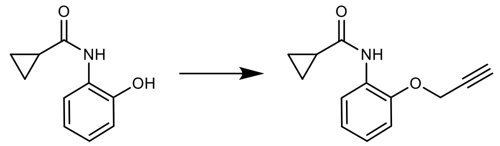

24 (155 mg, 875 µmol) and K_2CO_3 (285 mg, 2.06 mmol) were suspended in acetone (8 mL). Propargyl bromide (0.125 mL, 80 wt% in toluene, 1.12 mmol) was added and the reaction was refluxed overnight. Solvent was removed *in vacuo*, and the product was extracted (EtOAc/ 1 M $NaOH_{aq}$), organic layers were combined, dried over Na_2SO_4, filtered, and concentrated *in vacuo* to give intermediate **25** as a white solid (175 mg, 93% yield).

^{1}H NMR (400 MHz, $CDCl_3$) δ 8.37 (d, *J* = 6.0 Hz, 1H), 7.96 (s, 1H), 7.05 – 6.97 (m, 3H), 4.78 (d, *J* = 2.4 Hz, 2H), 2.56 (t, *J* = 2.4 Hz, 1H), 1.11 – 1.07 (m, 2H), 0.87 – 0.82 (m, 2H).

***N*-(2-Hydroxyphenyl)-2-phenylacetamide (26)**

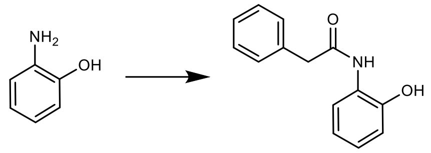

2-Aminophenol (510 mg, 4.67 mmol) was dissolved in dry dichloromethane (25mL). On an ice bath, *N*,*N*-diisopropylethylamine (685 µL, 5.14 mmol) and 2-phenylacetyl chloride (977 µL, 5.61 mmol) were added and the mixture stirred overnight. The precipitate was filtered off, and the filtrate was extracted (EtOAc/1 M $NaOH_{aq}$), aqueous layers were combined, acidified to pH 1 using 4 M HCl_{aq}. The product was filtered off as a precipitate and dried *in vacuo* to give intermediate **26** as a yellow solid (234 mg, 22% yield).

^{1}H NMR (400 MHz, $CDCl_3$) δ 8.69 (s, 1H), 7.48 – 7.33 (m, 5H), 7.15 – 7.06 (m, 1H), 7.00 (dd, *J* = 8.1, 1.2 Hz, 1H), 6,85 – 6,72 (m, 2H), 3,83 (s, 2H).

***N*-(2-(Propargyloxy)phenyl)-2-phenylacetamide (27)**

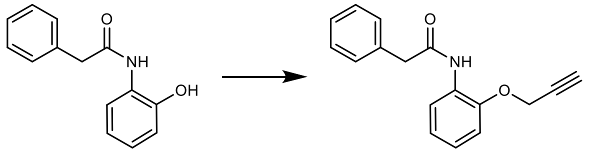

26 (212 mg, 0.93 mmol) and K_2CO_3 (300 mg, 2.17 mmol) were suspended in acetone (8 mL). Propargyl bromide (0.130 mL, 80 wt% in toluene, 1.17 mmol) was added and the reaction was refluxed overnight. Solvent was removed *in vacuo*, and the product was extracted (EtOAc/ 1 M $NaOH_{aq}$), organic layers were combined, dried over Na_2SO_4, filtered, and concentrated *in vacuo*. Column chromatography (DCM) gave intermediate **27** as a yellow oil (200 mg, 81% yield).

^{1}H NMR (400 MHz, $CDCl_3$) δ 8,40 – 8,32 (m, 1H), 7,78 (s, 1H), 7,43 – 7.30 (m, 5H), 7.03 – 6.96 (m, 2H), 6.93 – 6.89 (m, 1H), 4.57 (d, *J* = 2.4 Hz, 2H), 3.76 (s, 2H), 2.49 (t, *J* = 2.4 Hz, 1H).

***N*-(4-Hydroxy-3-nitrophenyl)acetamide (28)**

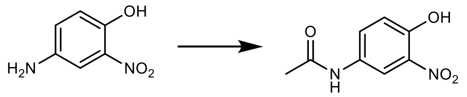

4-Amino-2-nitrophenol (912 mg, 5.92 mmol) was dissolved in water (10 mL). The mixture was cooled in an ice bath, acetic anhydride (620 µL, 6.57 mmol) was added dropwise, and the mixture allowed to come to room temperature over 2 h. The product was filtered off as a precipitate and dried *in vacuo* to give intermediate **28** as a brown solid (905 mg, 78% yield).

^{1}H NMR (400 MHz, DMSO-d_6) δ 11.57 (s, 1H), 9.46 (s, 1H), 8.93 (d, *J* = 2.8 Hz, 1H), 7.89 (dd, *J* = 8.9, 2.9 Hz, 1H), 7.02 (d, *J* = 9.0 Hz, 1H), 2.14 (s, 3H).

N-(4-Propargyloxy-3-nitrophenyl)acetamide (29)

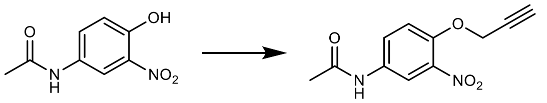

28 (840 mg, 4.28 mmol) and K_2CO_3 (1.40 g, 10.13 mmol) were suspended in acetone (15 mL). Propargyl bromide (600 µL, 80 wt% in toluene, 5.39 mmol) was added and the reaction was refluxed overnight. Solvent was removed *in vacuo*, and the product was extracted (EtOAc/ 1 M $NaOH_{aq}$), organic layers were combined, dried over Na_2SO_4, filtered, and concentrated *in vacuo*. Column chromatography (1:2 EtOAc/Hex.) gave intermediate **29** as an orange solid (752 mg, 75% yield).

^{1}H NMR (400 MHz, DMSO-d_6) δ 9.57 (s, 1H), 8.98 (d, *J* = 2.6 Hz, 1H), 8.04 (dd, *J* = 9.1, 2.9 Hz, 1H), 7.33 (d, *J* = 9.2 Hz, 1H), 5.08 (d, *J* = 2.4 Hz, 2H), 3.73 (t, *J* = 2.4 Hz, 1H), 2.15 (s, 3H).

***N,N'*-(3-(Propargyloxy)phenylene)-1,4-diacetamide (30)**

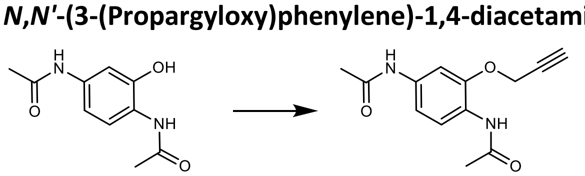

29 (396 mg, 1.69 mmol) and iron powder (944 mg, 16.90 mmol) were suspended in acetic acid (15 mL). The reaction was stirred at r.t. overnight. Afterwards, acetic anhydride (0.31 mL, 3.28 mmol) was added and the reaction mixture was heated to 55 °C and stirred overnight Solids were removed by filtration over Celite® and solvent was removed *in vacuo*. Column chromatography (1:15 MeOH/DCM) gave intermediate **30** as a grey solid (308 mg, 74 % yield).

^{1}H NMR (400 MHz, DMSO-d_6) δ 9.84 (s, 1H), 9.12 (s, 1H), 8.05 (d, *J* = 2.3 Hz, 1H), 7.42 (dd, *J* = 8.9, 2.3 Hz, 1H), 7.02 (d, *J* = 8.9 Hz, 1H), 4.80 (d, *J* = 2.3 Hz, 2H), 3.59 (t, *J* = 2.3 Hz, 1H), 2.08 (s, 3H), 1.99 (s, 3H).

8-(Propargyloxy)quinoline (31)

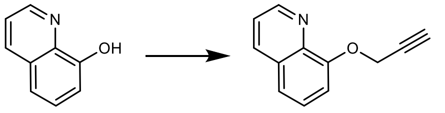

8-Hydroxyquinoline (80 mg, 0.55 mmol) and K_2CO_3 (305 mg, 2.20 mmol) were suspended in THF (5 mL). Propargyl bromide (600 µL, 80 wt% in toluene, 5.39 mmol) was added and the reaction was refluxed for two days. Solvent was removed *in vacuo*. Column chromatography (1:2 EtOAc/Hex.) gave intermediate **31** as a light yellow oil (68 mg, 67% yield).

^{1}H NMR (400 MHz, $CDCl_3$) δ 8.95 (dd, *J* = 4.2, 1.7 Hz, 1H), 8.15 (dd, *J* = 8.3, 1.7 Hz, 1H), 7.55 – 7.39 (m, 3H), 7.29 – 7.25 (m, 1H), 5.04 (d, *J* = 2.4 Hz, 2H), 2.53 (t, *J* = 2.4 Hz, 1H).

8-(Propargyloxy)quinoline-2-carboxylic acid (32)

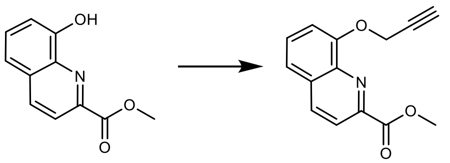

Methyl 8-(propargyloxy)quinoline-2-carboxylate (75 mg, 0.37 mmol) and K_2CO_3 (205 mg, 1.48 mmol) were suspended in acetone (5 mL). Propargyl bromide (120 µL, 80 wt% in toluene, 1.08 mmol) was added and the reaction was refluxed overnight. Solvent was removed *in vacuo*, and the product was extracted (EtOAc/ 1 M $NaOH_{aq}$), organic layers were combined, dried over Na_2SO_4, filtered, and concentrated *in vacuo*. Crystallisation (DCM/Hex.) gave intermediate **32** as light yellow solids (752 mg, 75% yield).

^{1}H NMR (400 MHz, $CDCl_3$) δ 8.30 (d, *J* = 8.6 Hz, 1H), 8.24 (d, *J* = 8.5 Hz, 1H), 7.64 – 7.57 (m, 1H), 7.51 (dd, *J* = 8.3, 1.2 Hz, 1H), 7.34 (dd, *J* = 7.7, 1.2 Hz, 1H), 5.05 (d, *J* = 2.4 Hz, 2H), 4.06 (s, 3H), 2.55 (d, *J* = 2.4 Hz, 1H).

2-(Propargyloxy)nitrobenzene (9)

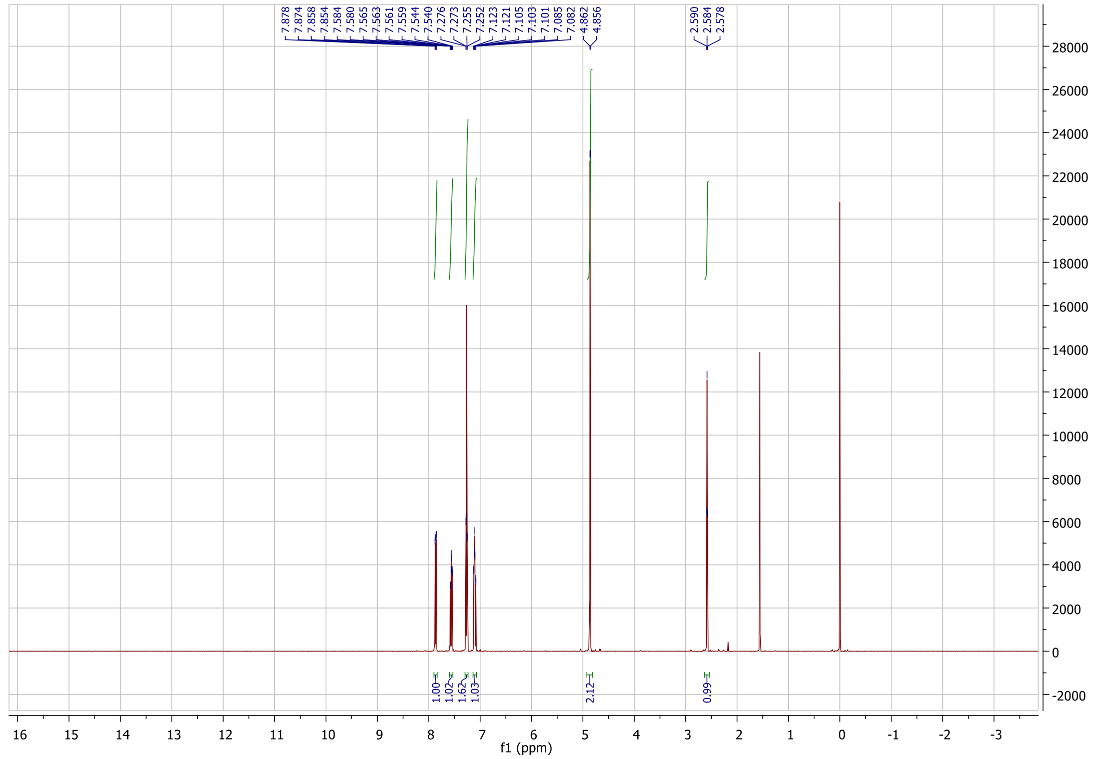

2-(Propargyloxy)aniline (10)

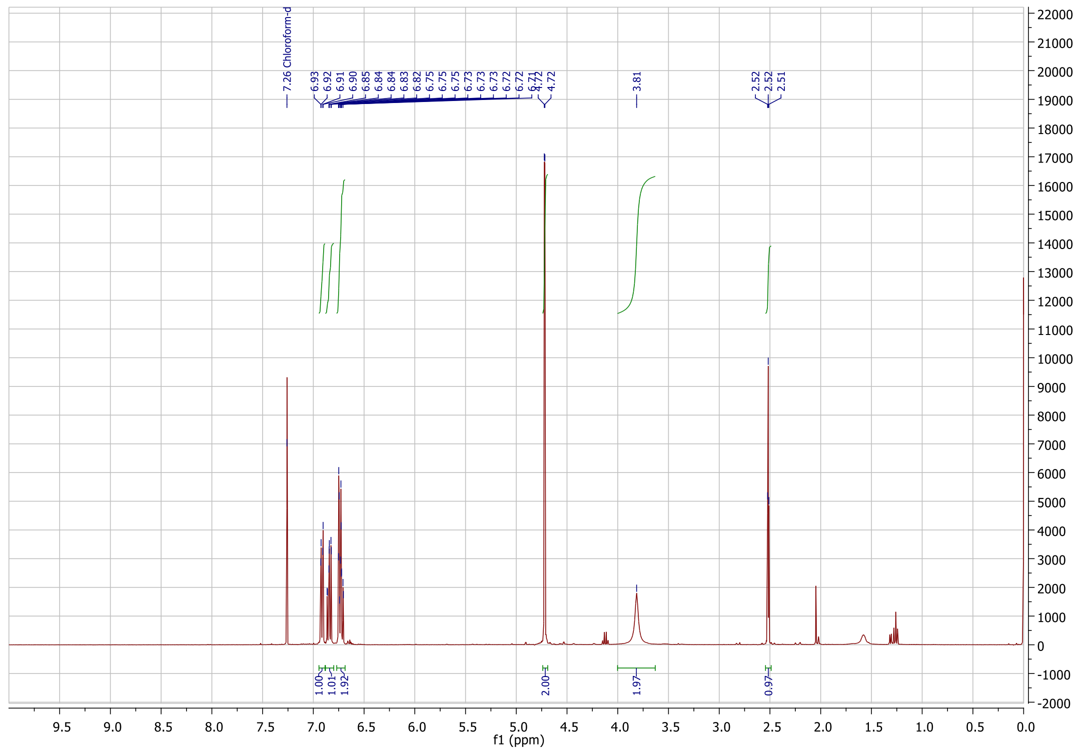

N-Methyl 2-(propargyloxy)aniline (11)

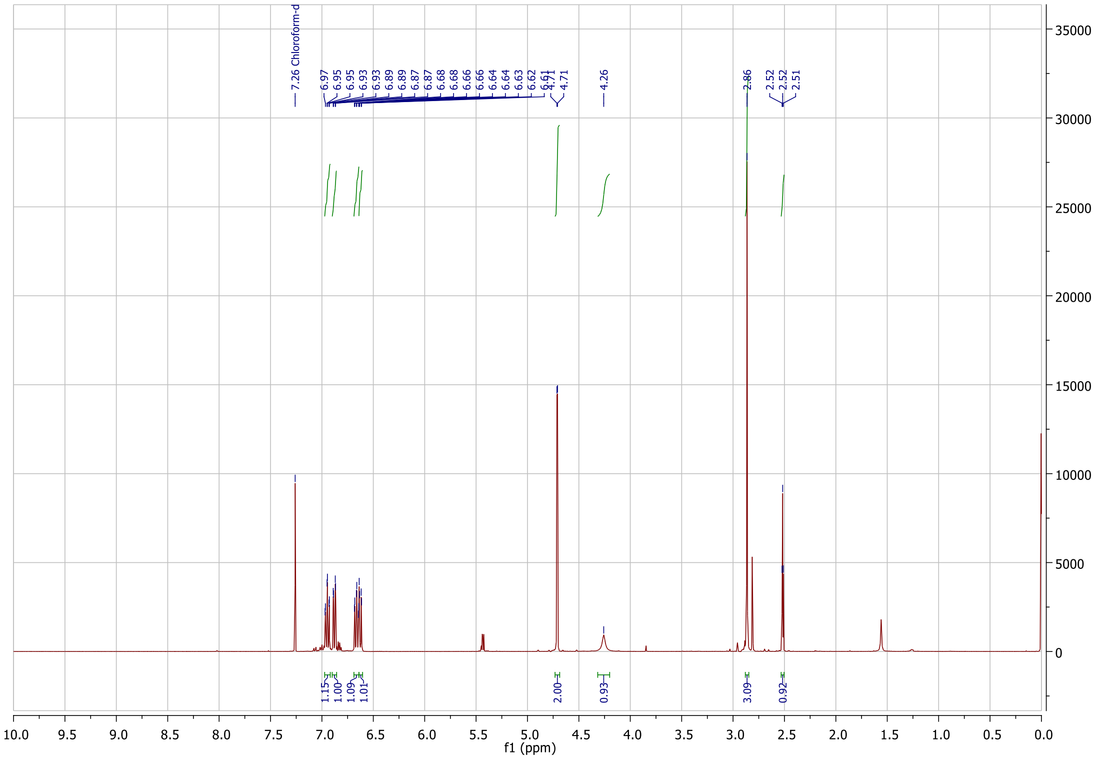

N-(2-(Propargyloxy)phenyl)acetamide (12)

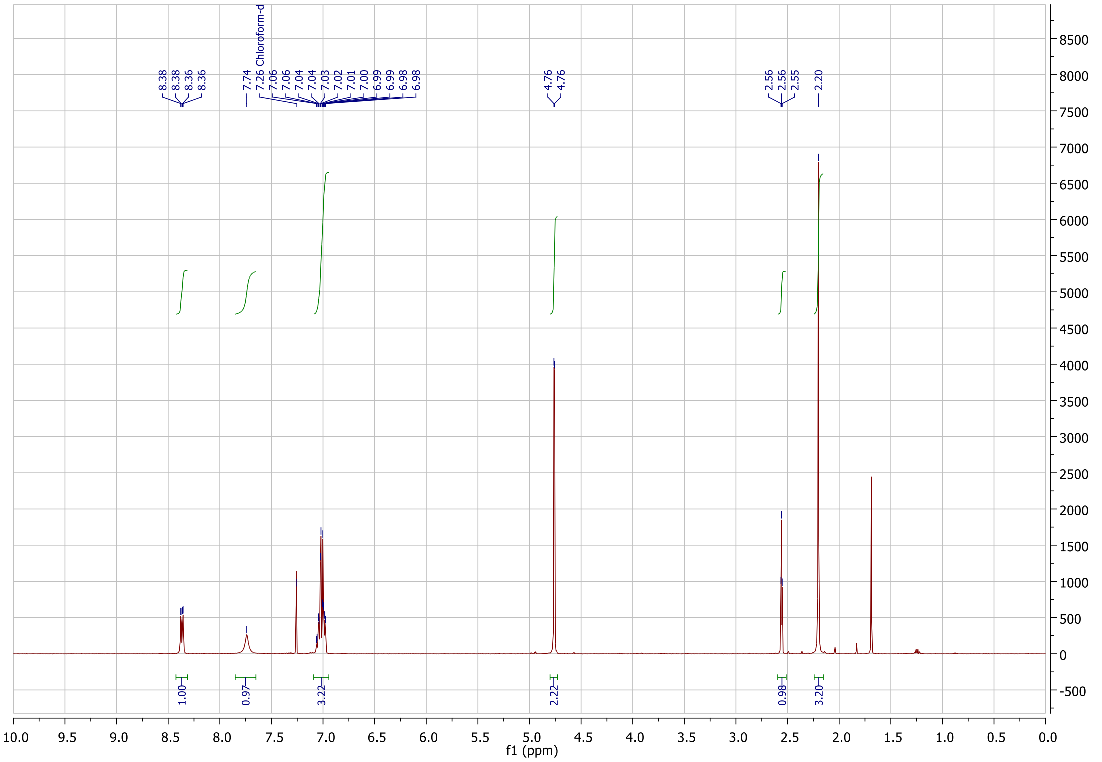

N-(2-(Propargyloxy)phenyl)methanesulfonamide (13)

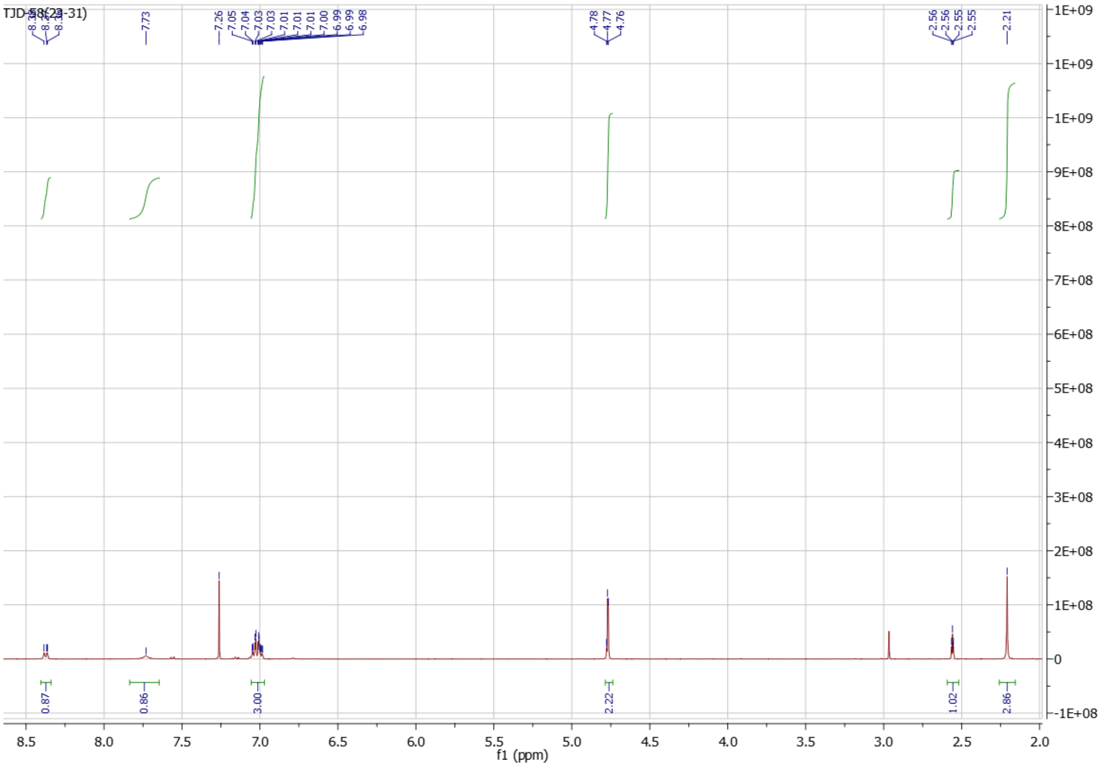

Methyl 3-(propargyloxy)benzoate (14)

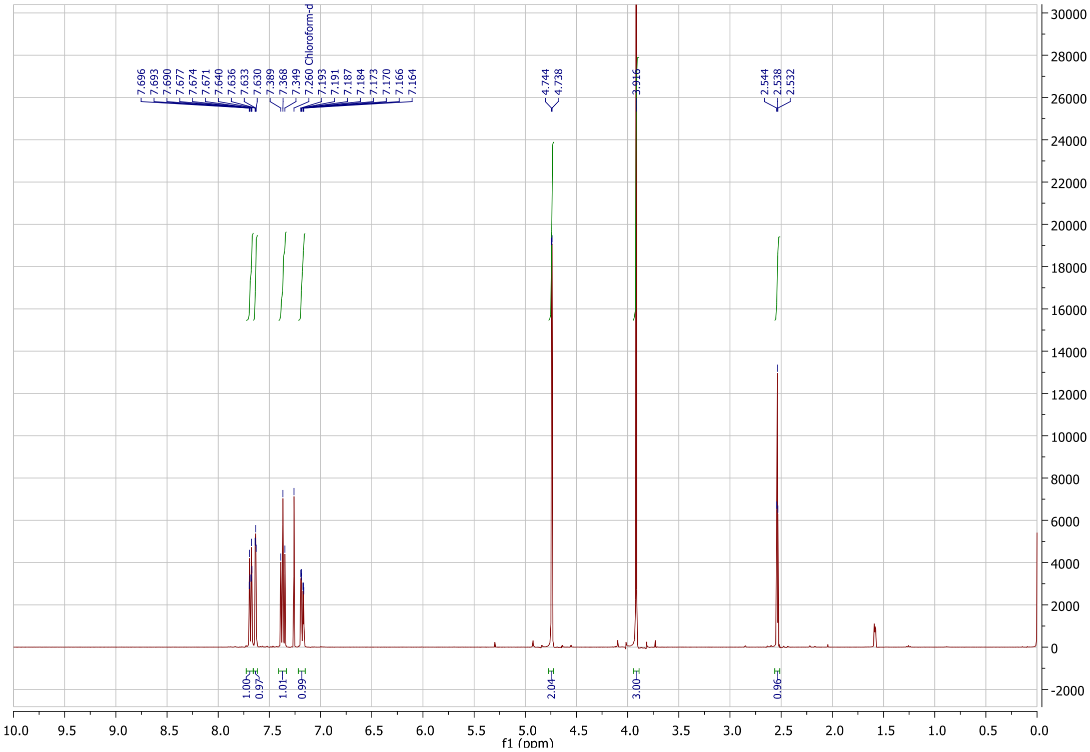

Methyl 4-nitro-3-(propargyloxy)benzoate (15)

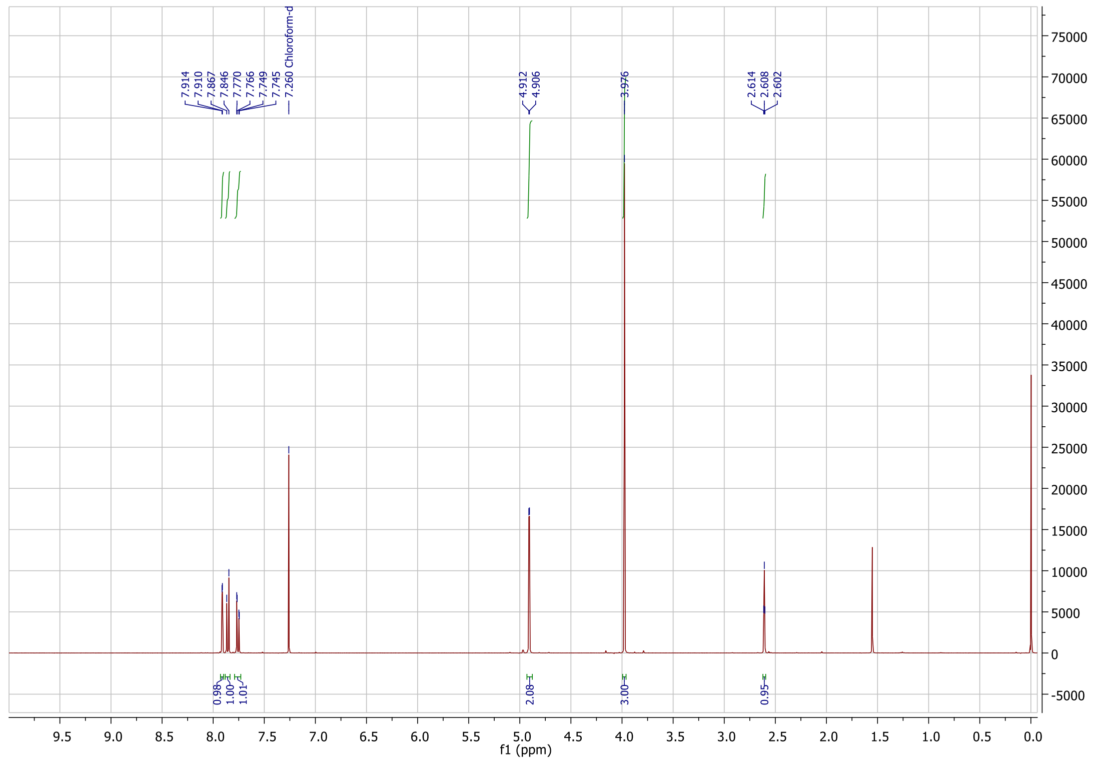

Methyl 4-amino-3-(propargyloxy)benzoate (16)

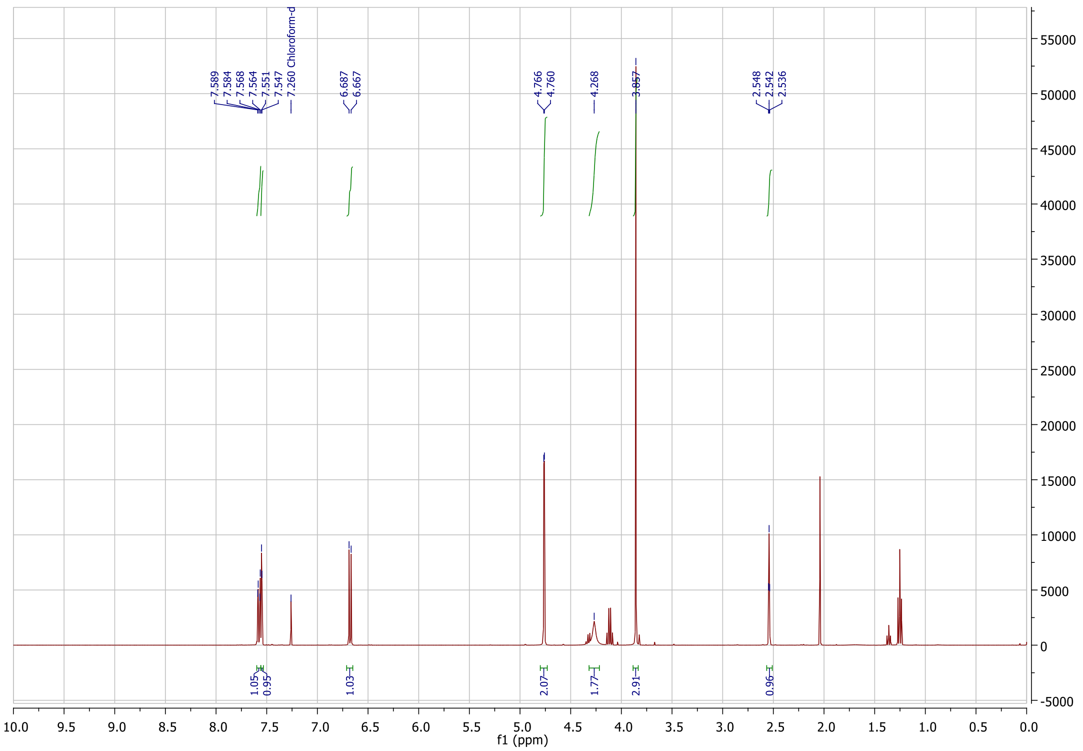

Methyl 3-hydroxy-4-acetamidobenzoate (17)

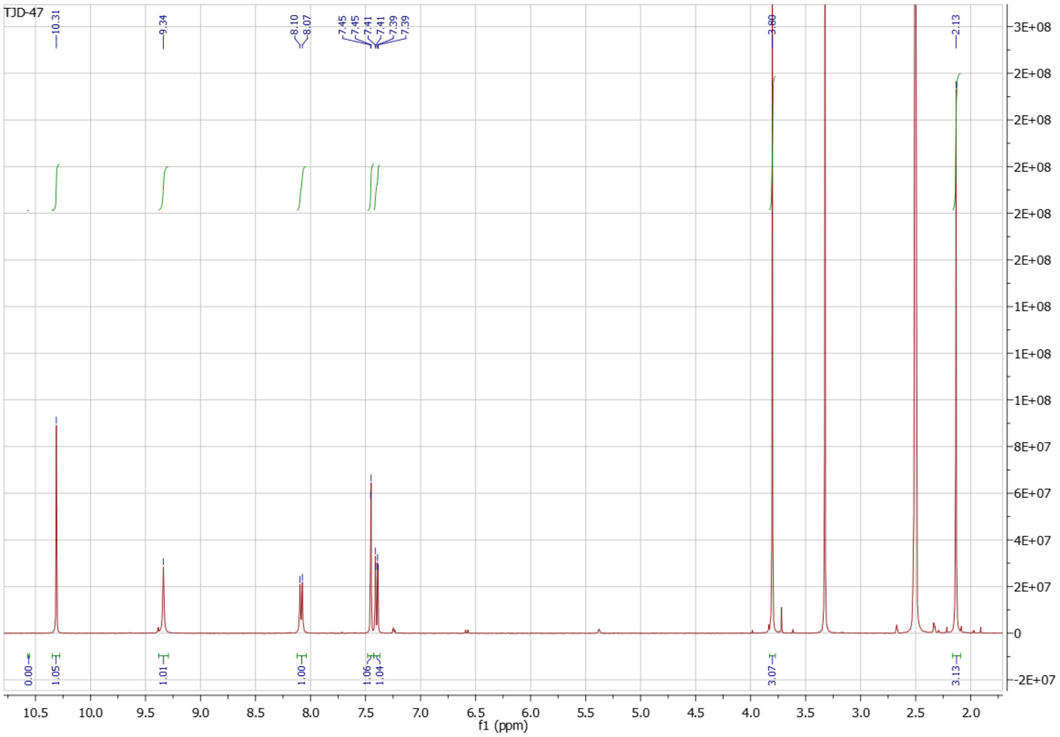

Methyl 3-(propargyloxy)-4-acetamidobenzoate (18)

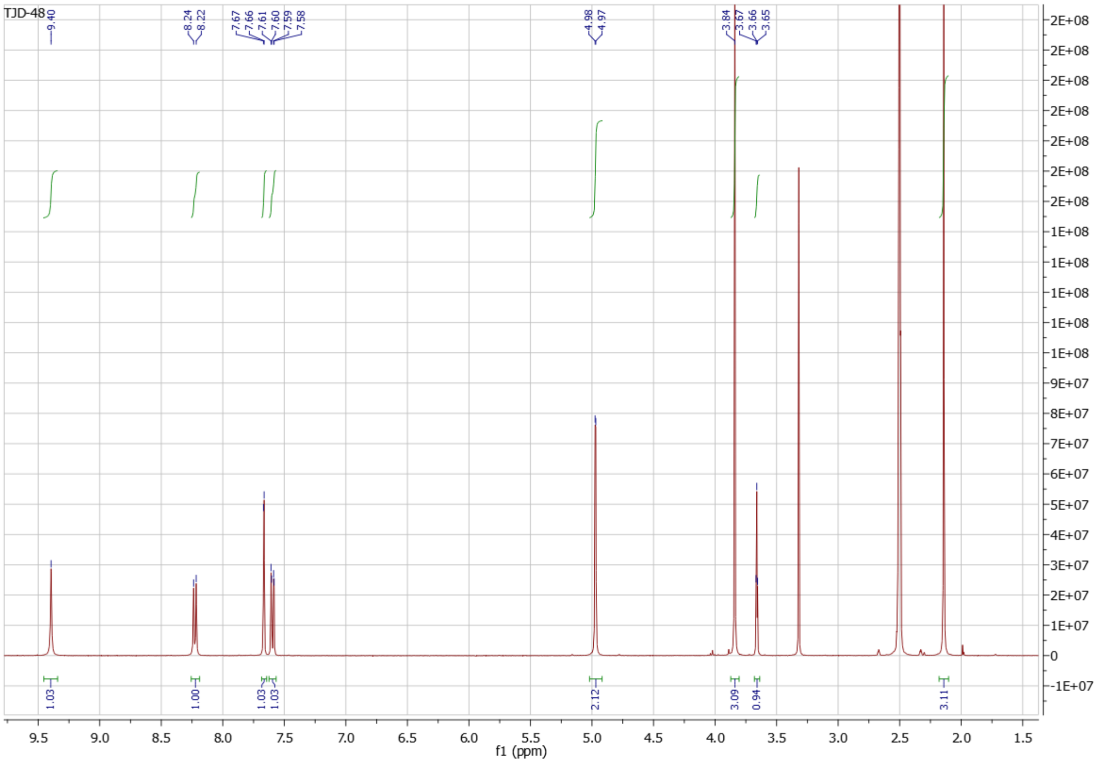

(Propargyloxy)benzene (19)

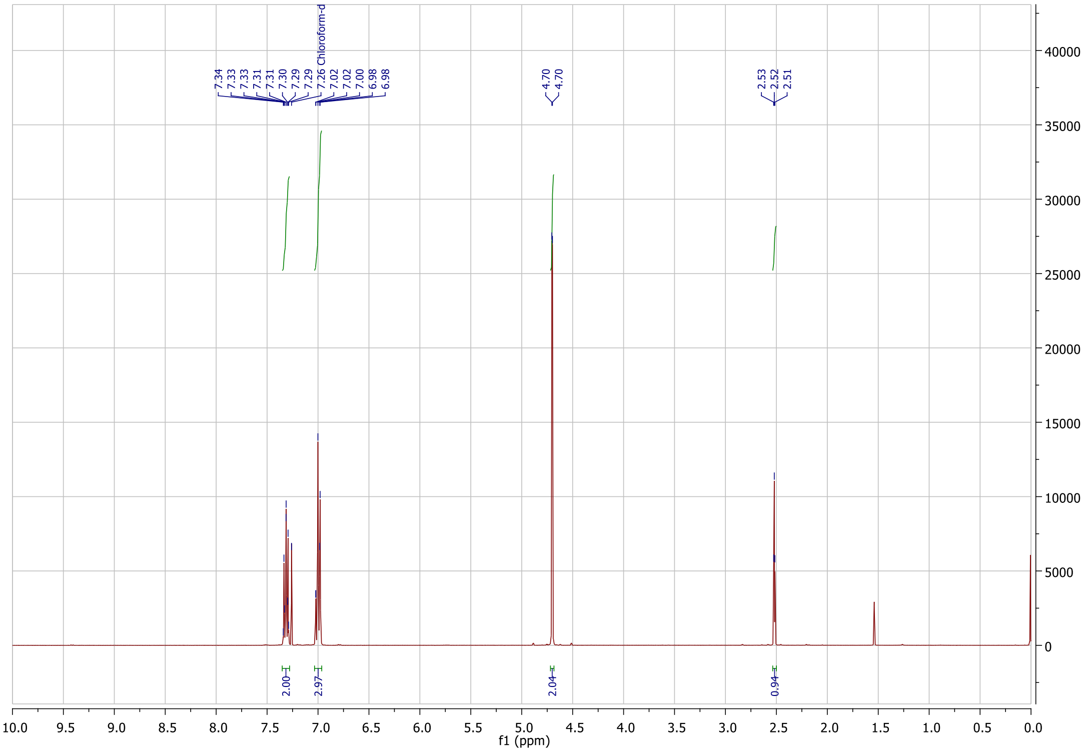

2-(Propargyloxy)phenol (20)

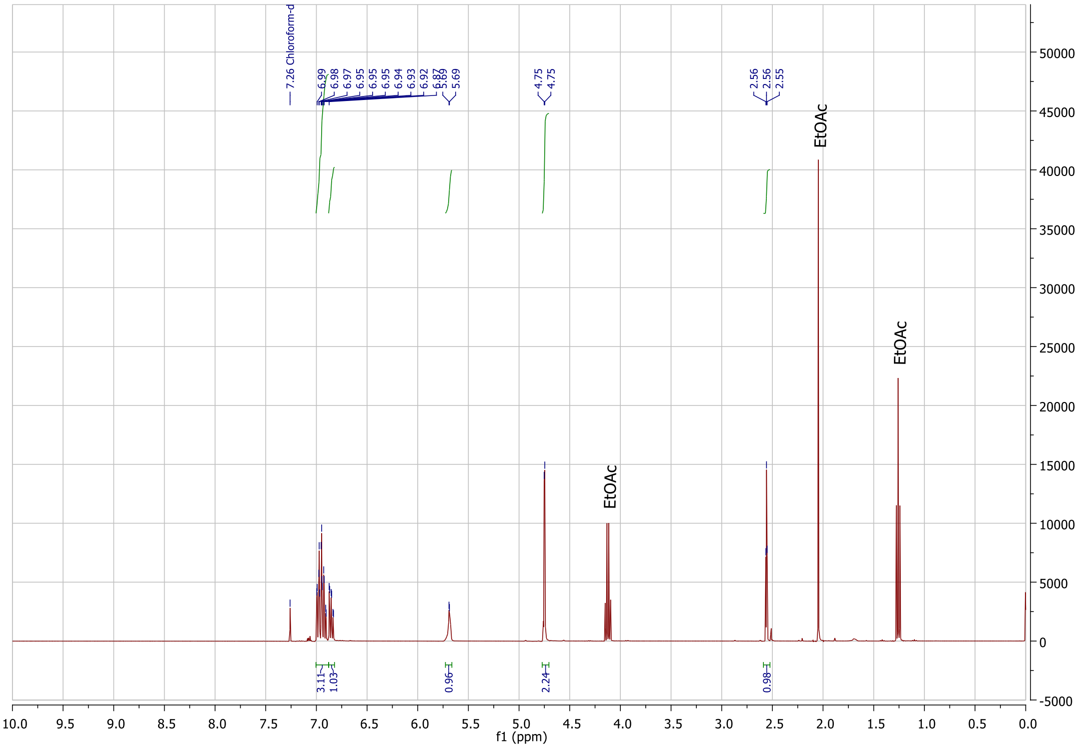

2-((Propargyloxy)phenyl)methanol (21)

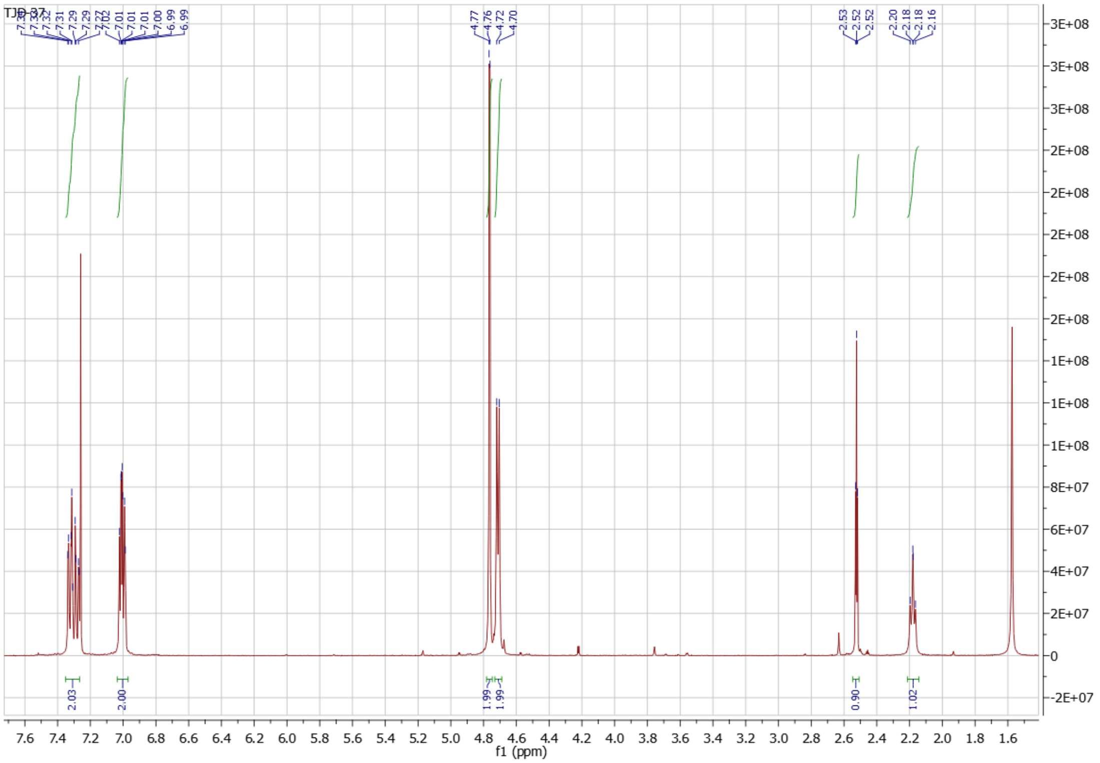

N-(3-(Propargyloxy)phenyl)acetamide (22)

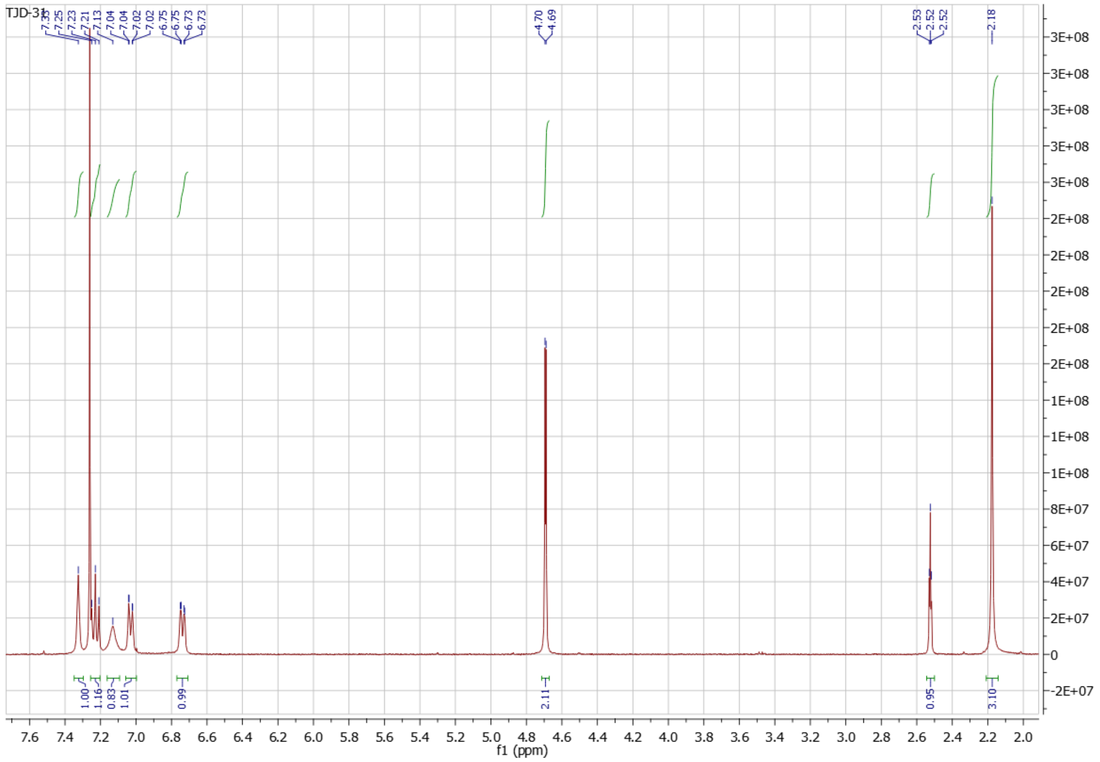

***N*-(4-(Propargyloxy)phenyl)acetamide (23)**

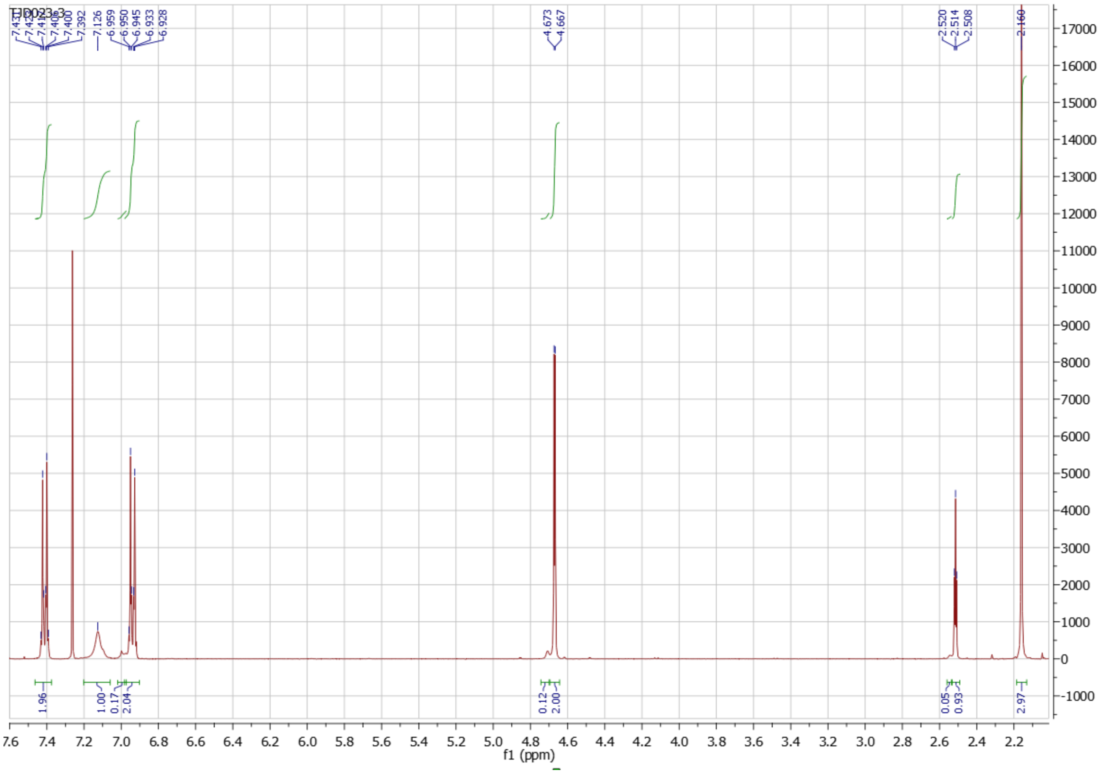

***N*-(2-Hydroxyphenyl)cyclopropanecarboxamide (24)**

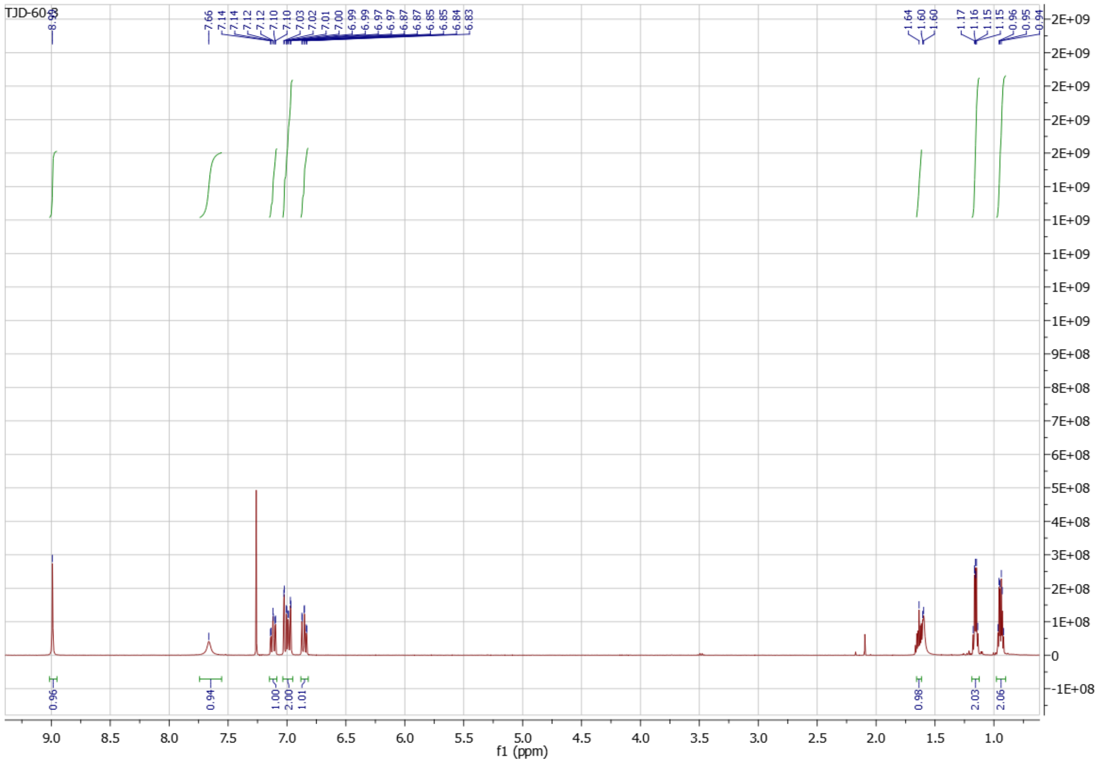

***N*-(2-(Propargyloxy)phenyl)cyclopropanecarboxamide (25)**

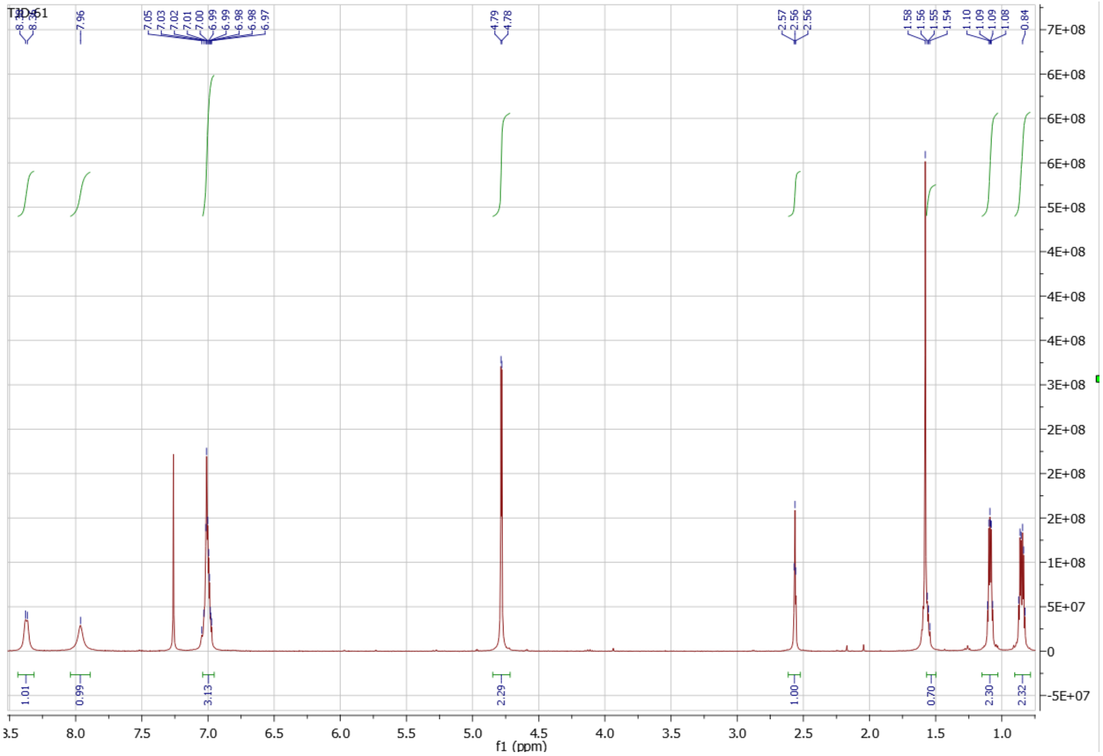

***N*-(2-Hydroxyphenyl)-2-phenylacetamide (26)**

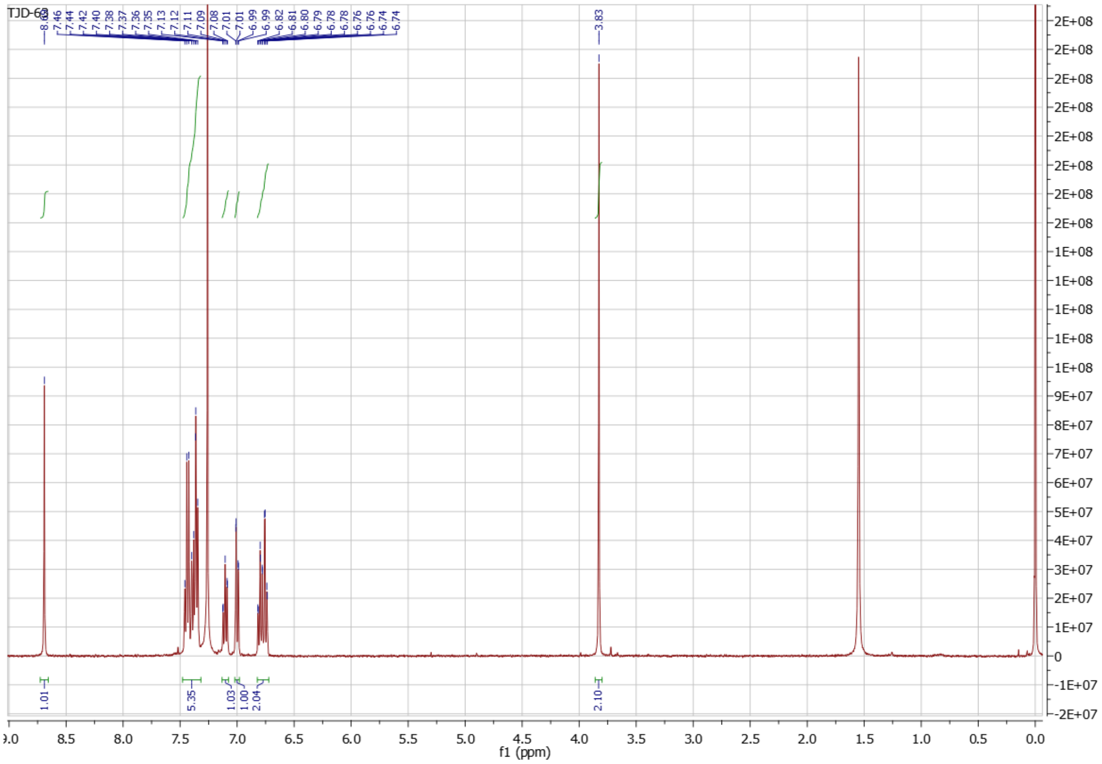

N-(2-(Propargyloxy)phenyl)-2-phenylacetamide (27)

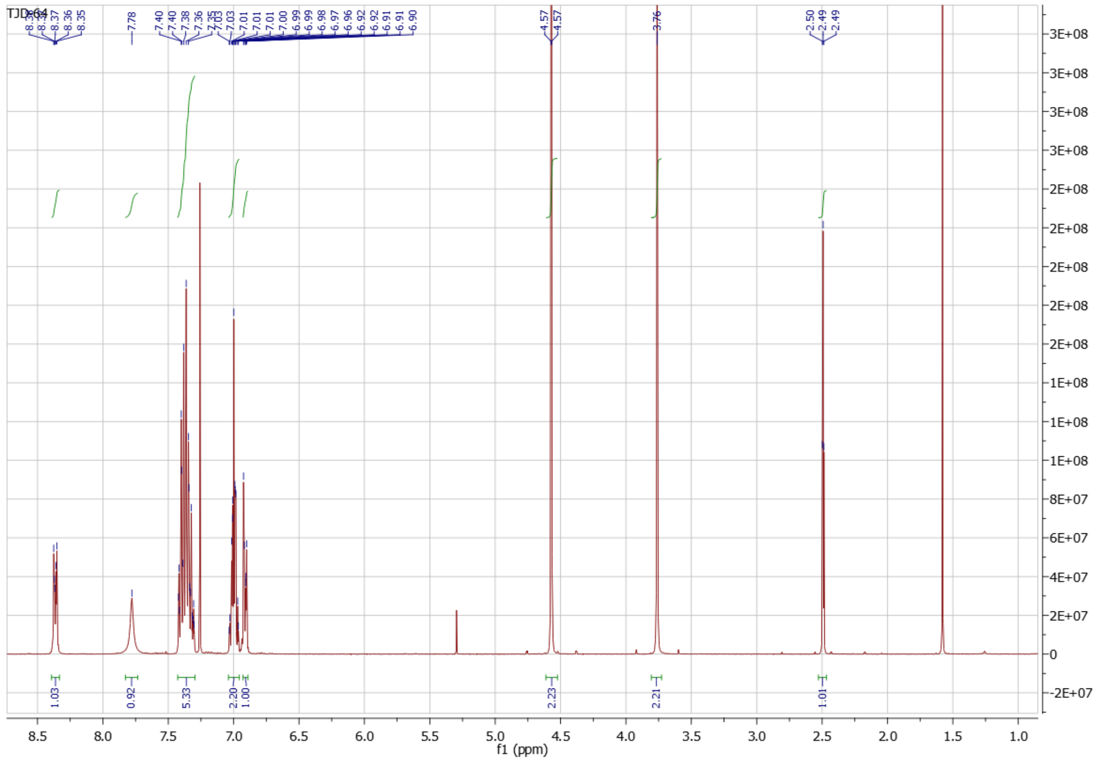

N-(4-Hydroxy-3-nitrophenyl)acetamide (28)

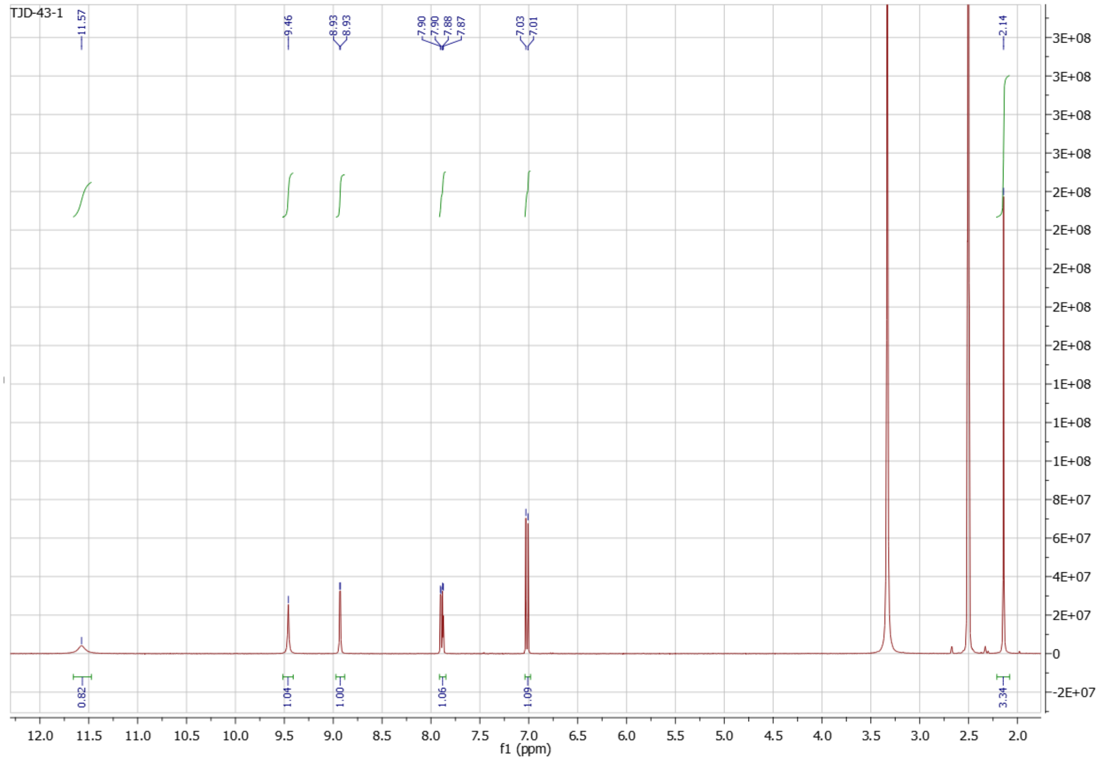

***N*-(4-Propargyloxy-3-ntrophenyl)acetamide (29)**

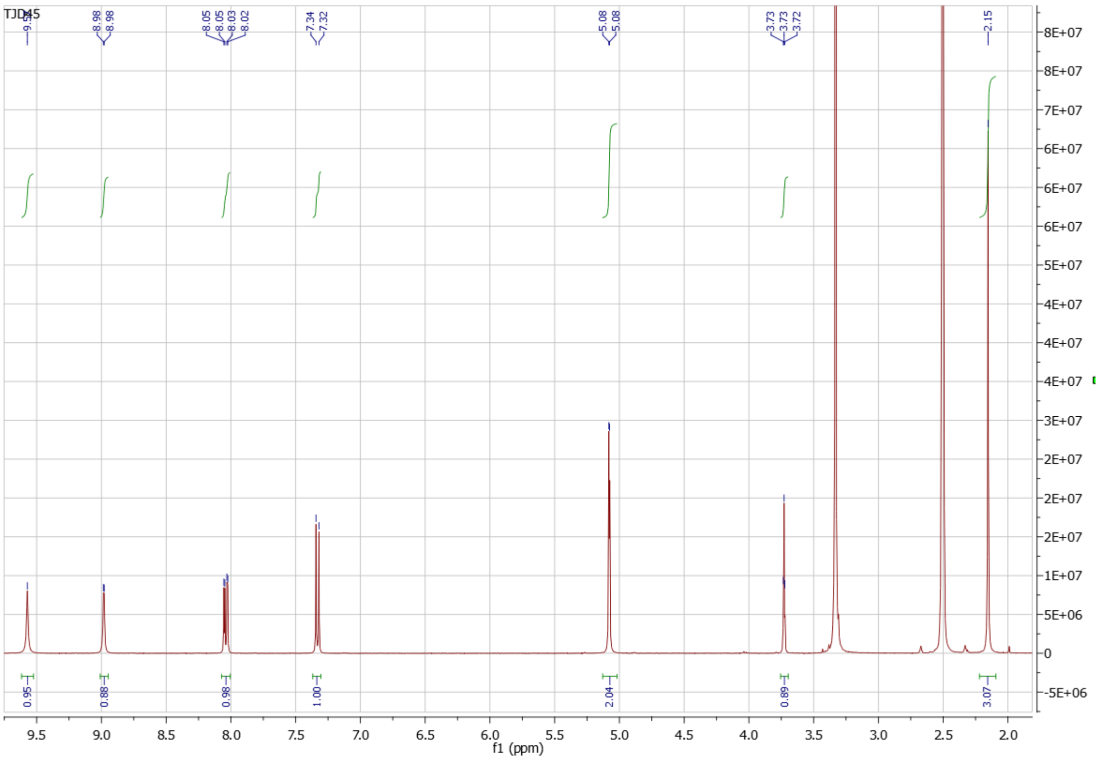

***N,N'*-(3-(Propargyloxy)phenylene)-1,4-diacetamide (30)**

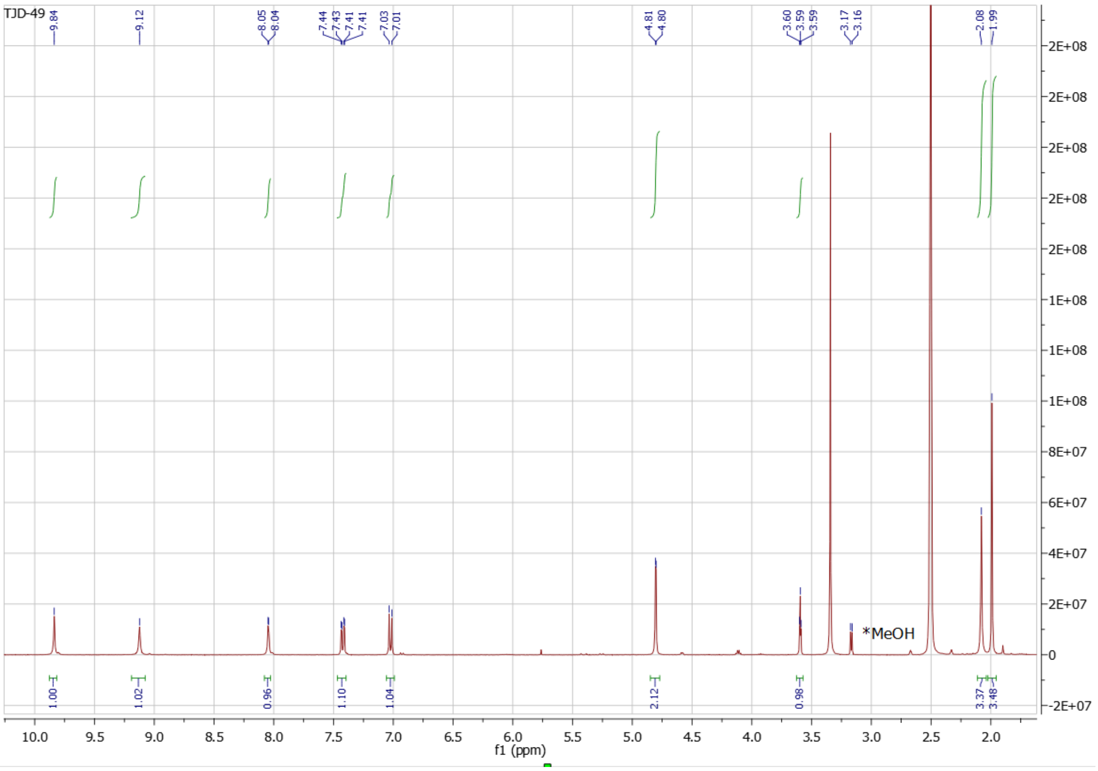

8-(Propargyloxy)quinoline (31)

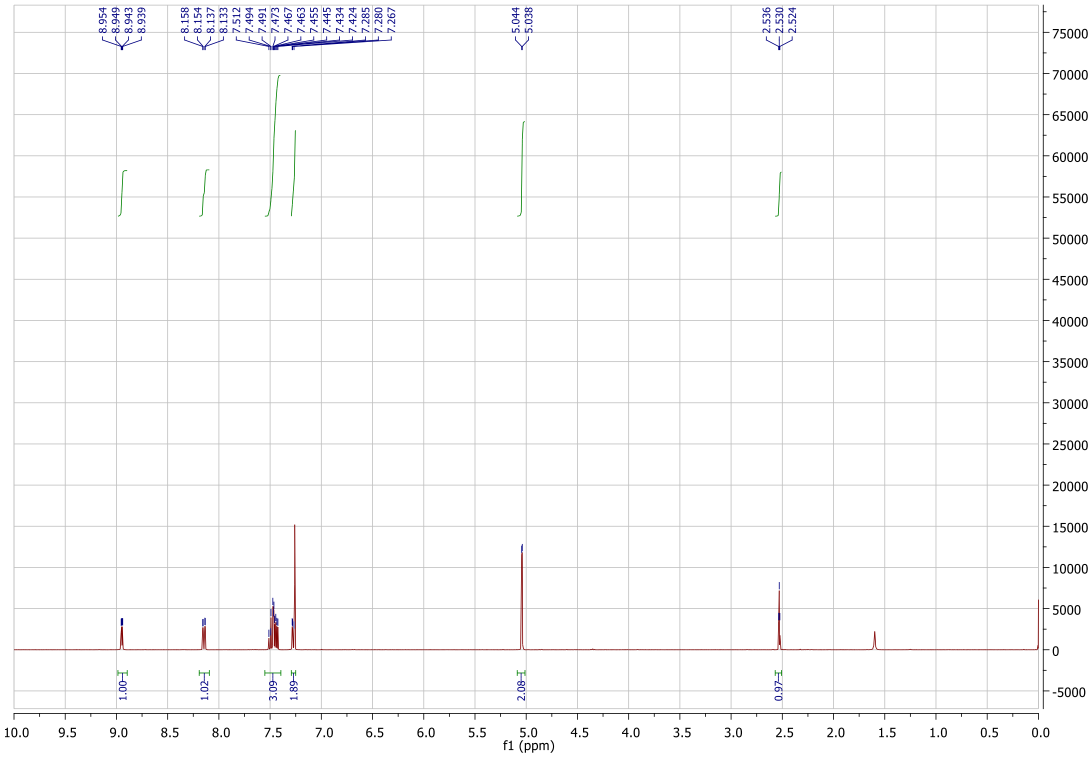

8-(Propargyloxy)quinoline-2-carboxylic acid (32)

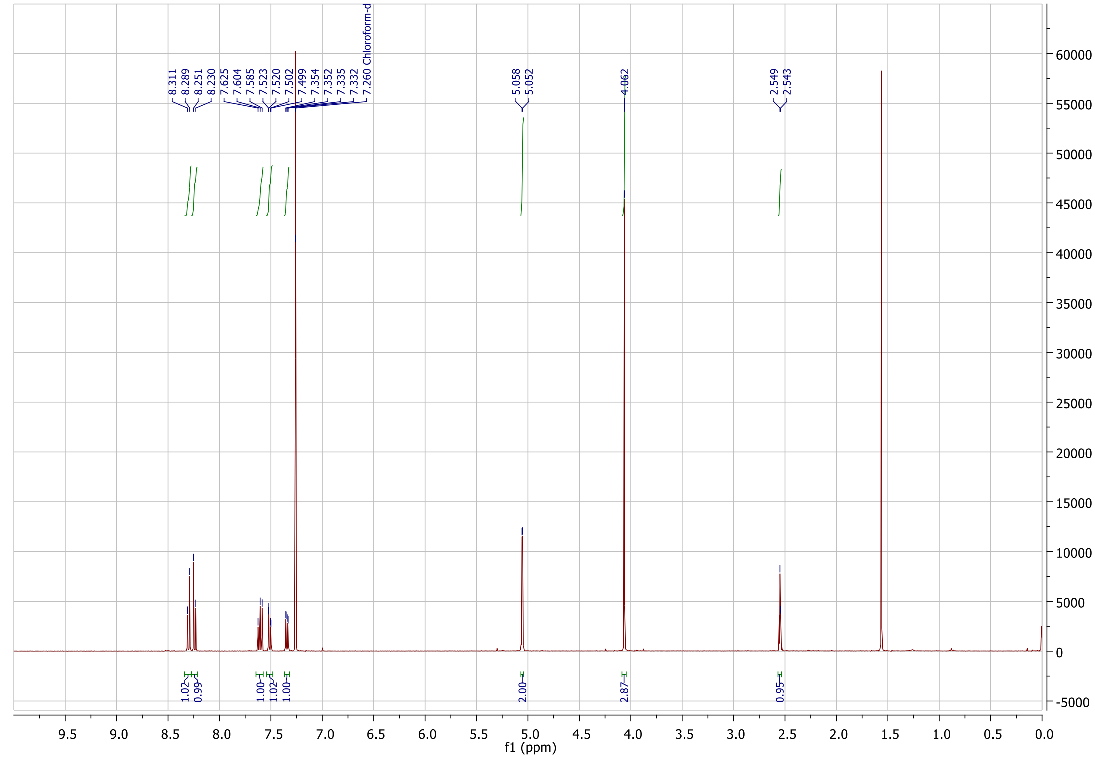

Tolyl 3-(4-((2-nitrophenoxy)methyl)-1,2,3-triazol-1-yl)-3-deoxy-1-thio-β-D-galactopyranoside (33)

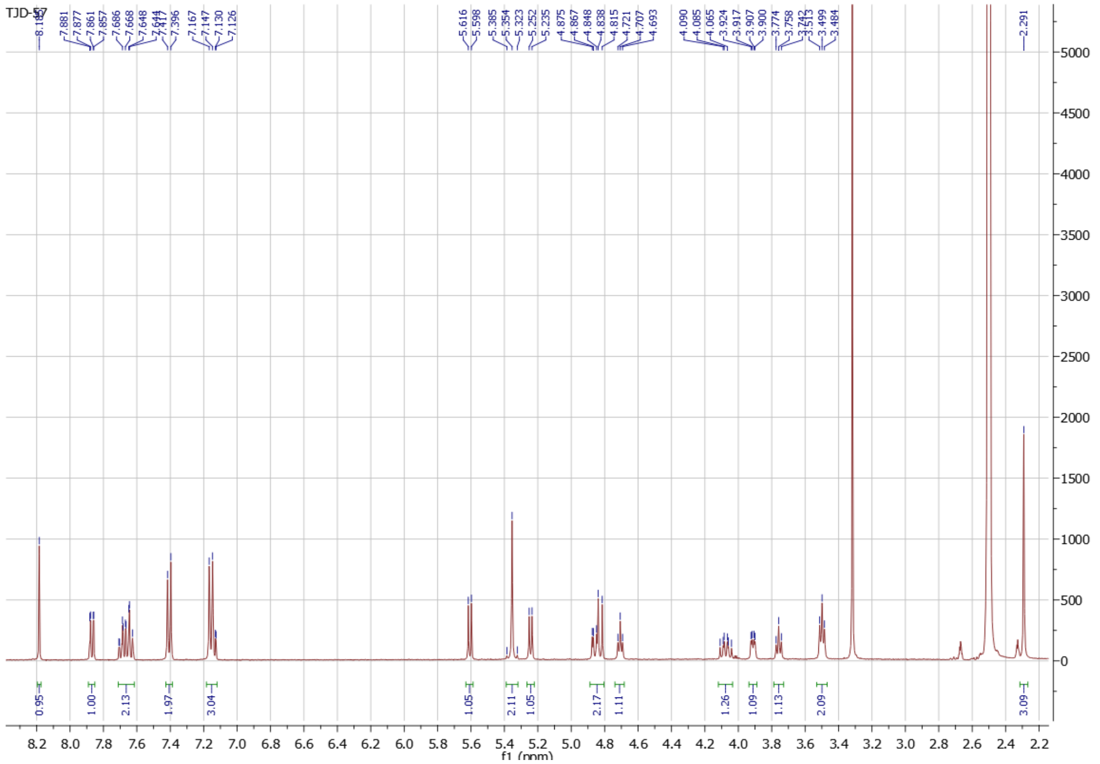

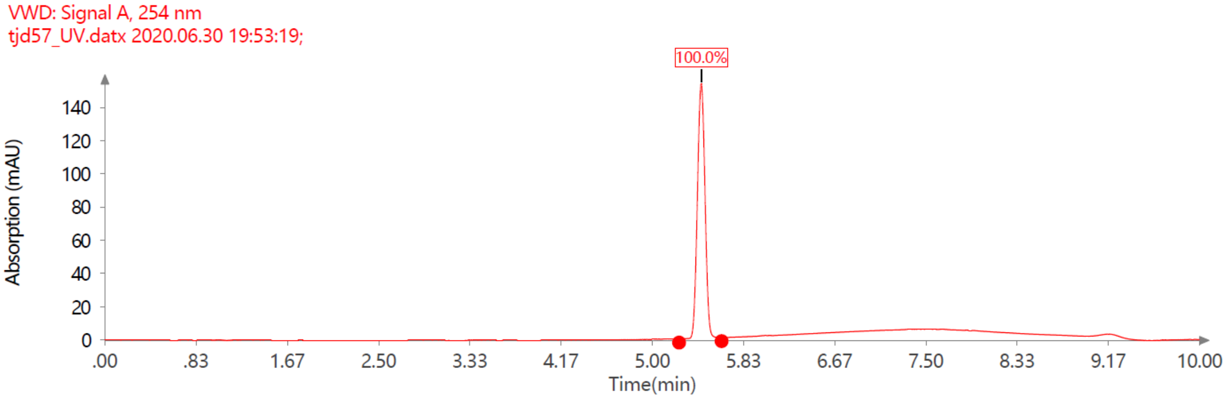

Time (Peak Maximum M:S/Minutes)	Maximum Intensity (c/s)	Time (Peak Centroid M:S/Minutes)	Peak Area	% Peak Area	Peak Resolution	Label
5.45	1.6E2	5.45	7.9E2	100.0	4.6	

Tolyl 3-(4-((2-aminophenoxy)methyl)-1,2,3-triazol-1-yl)-3-deoxy-1-thio-β-D-galactopyranoside (34)

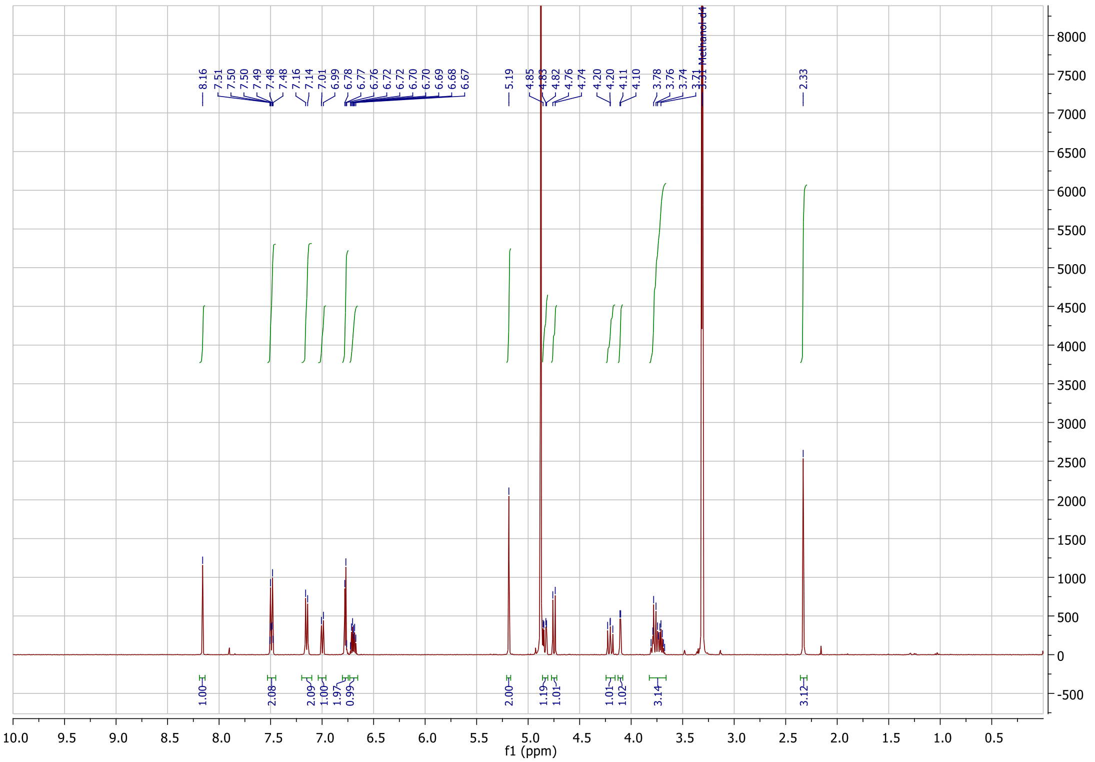

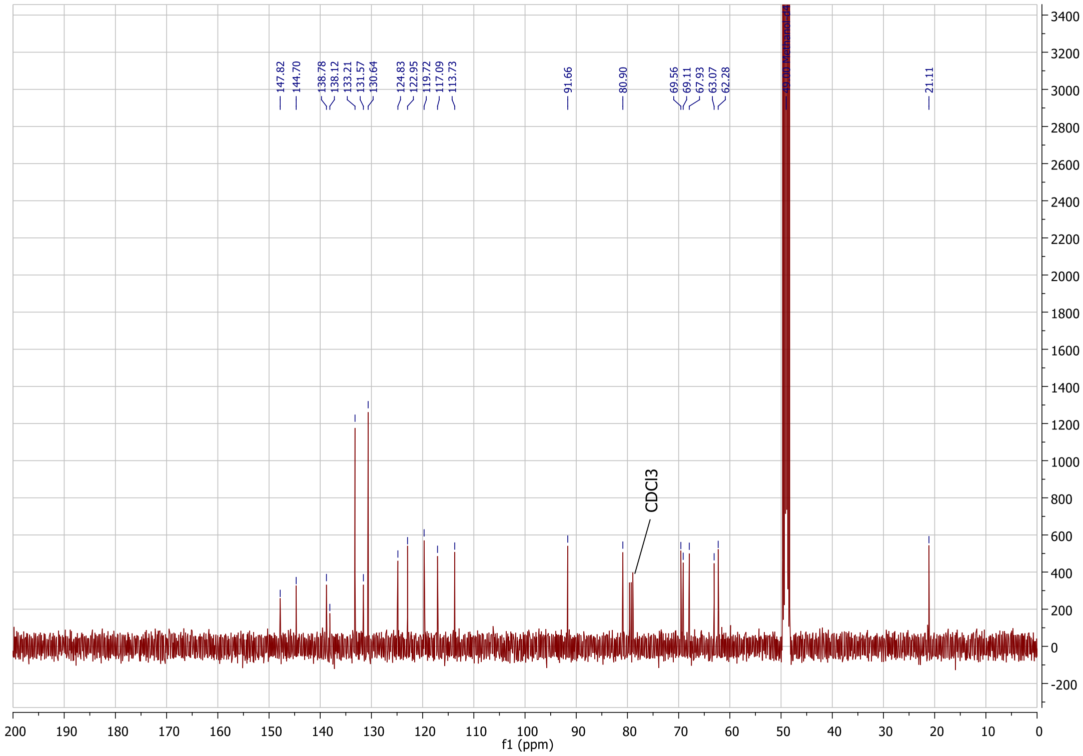

Chromatogram

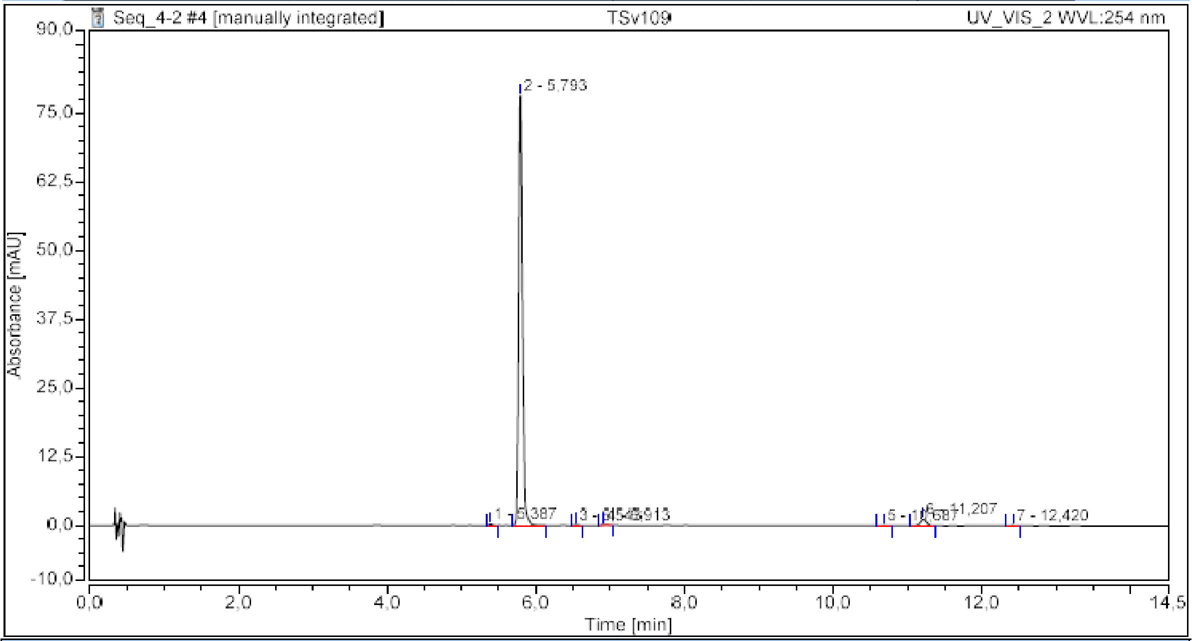

Integration Results

No.	Peak Name	Retention Time min	Area mAU*min	Height mAU	Relative Area %	Relative Height %	Amount n.a.
1		5,387	0,011	0,239	0,23	0,30	n.a.
2		5,793	4,489	78,278	96,49	97,74	n.a.
3		6,543	0,007	0,102	0,16	0,13	n.a.
4		6,913	0,013	0,112	0,27	0,14	n.a.
5		10,687	0,008	0,090	0,18	0,11	n.a.
6		11,207	0,116	1,166	2,49	1,46	n.a.
7		12,420	0,009	0,099	0,19	0,12	n.a.
**Total:**			**4,653**	**80,086**	**100,00**	**100,00**	

Tolyl 3-(4-(2-methylaminophenoxy)methyl)-1,2,3-triazol-1-yl)-3-deoxy-1-thio-β-D-galactopyranoside (35)

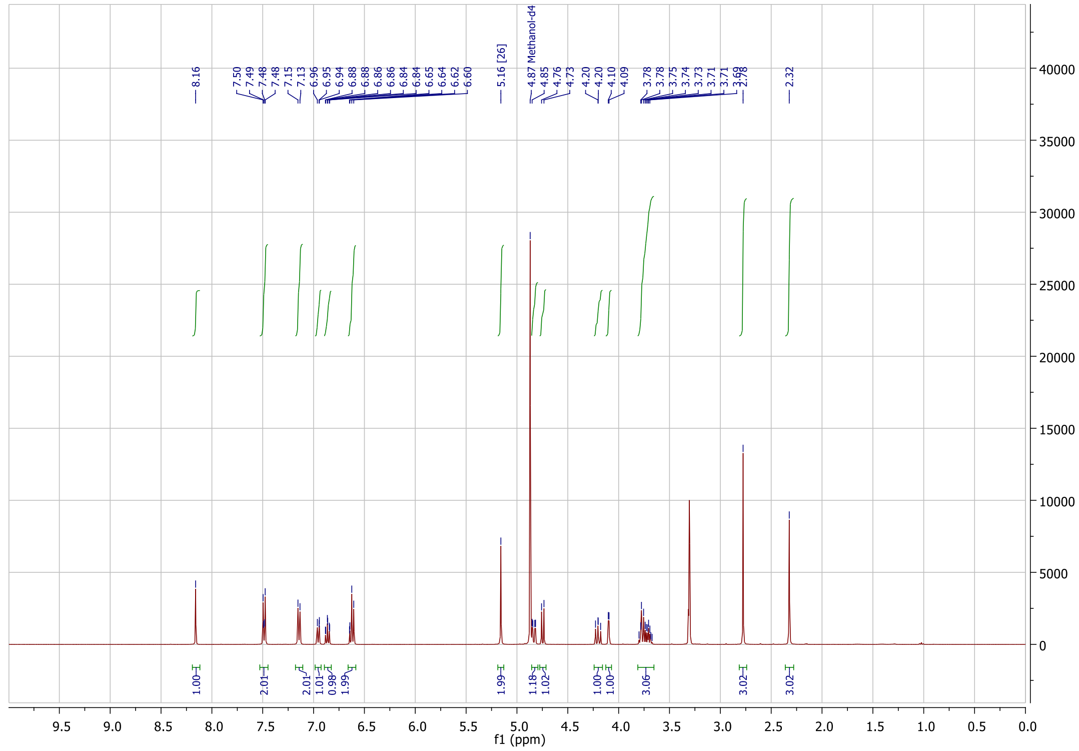

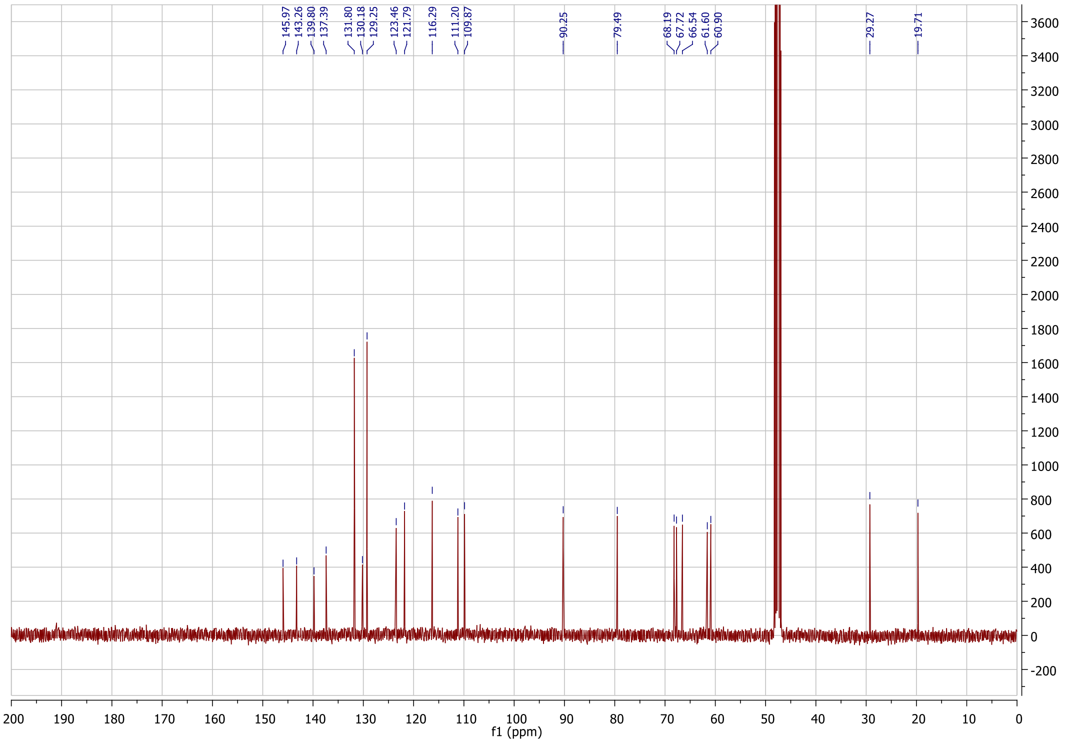

Chromatogram

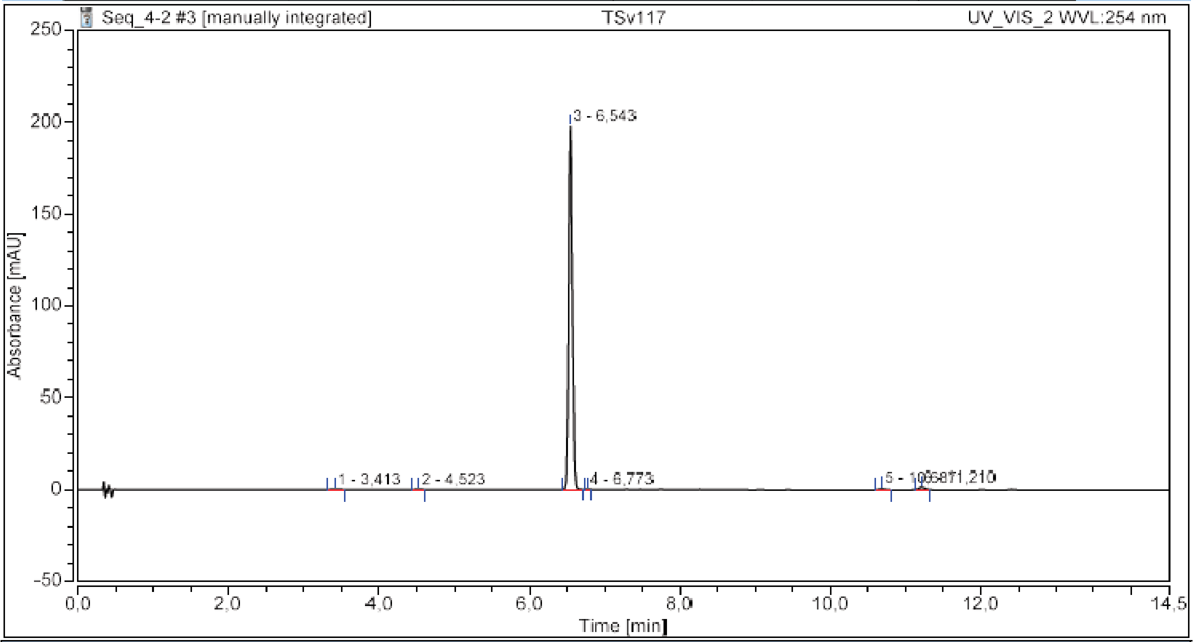

Integration Results

No.	Peak Name	Retention Time<br>min	Area<br>mAU*min	Height<br>mAU	Relative Area<br>%	Relative Height<br>%	Amount<br>n.a.
1		3,413	0,023	0,188	0,20	0,09	n.a.
2		4,523	0,016	0,189	0,14	0,09	n.a.
3		6,543	11,366	197,743	98,21	98,82	n.a.
4		6,773	0,013	0,282	0,11	0,14	n.a.
5		10,687	0,049	0,527	0,42	0,26	n.a.
6		11,210	0,106	1,181	0,92	0,59	n.a.
**Total:**			**11,573**	**200,111**	**100,00**	**100,00**	

Tolyl 3-(4-((2-acetamidophenoxy)methyl)-1,2,3-triazol-1-yl)-3-deoxy-1-thio-β-D-galactopyranoside (36)

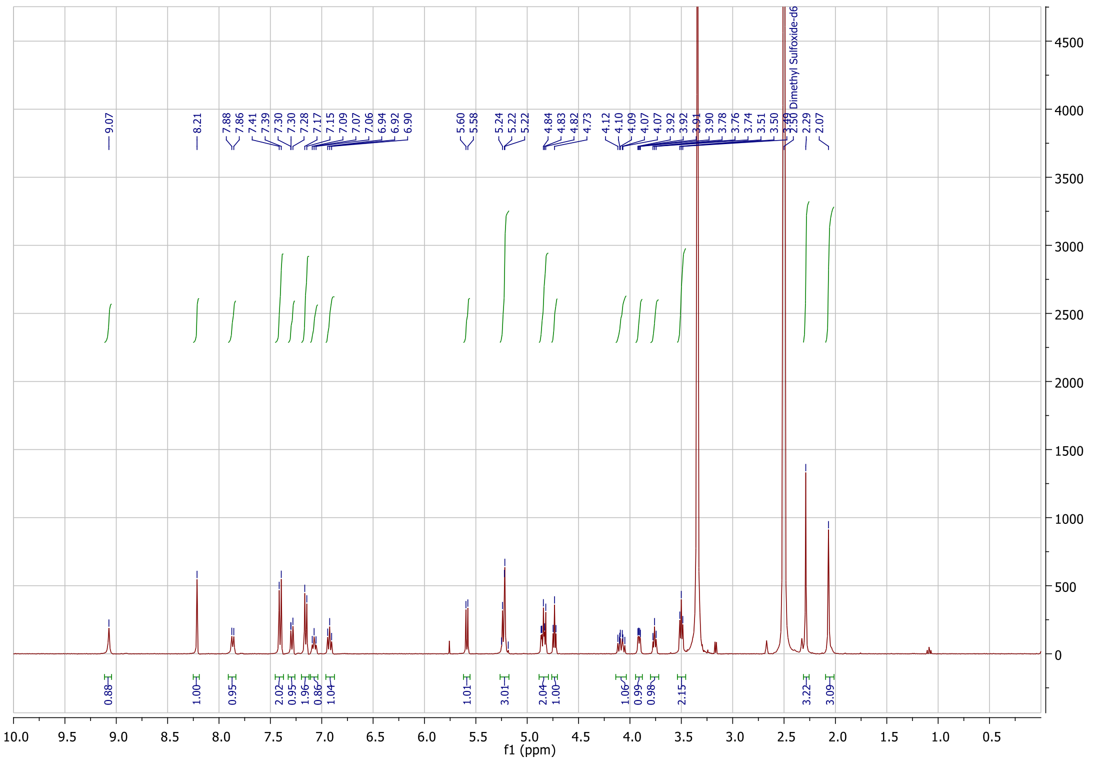

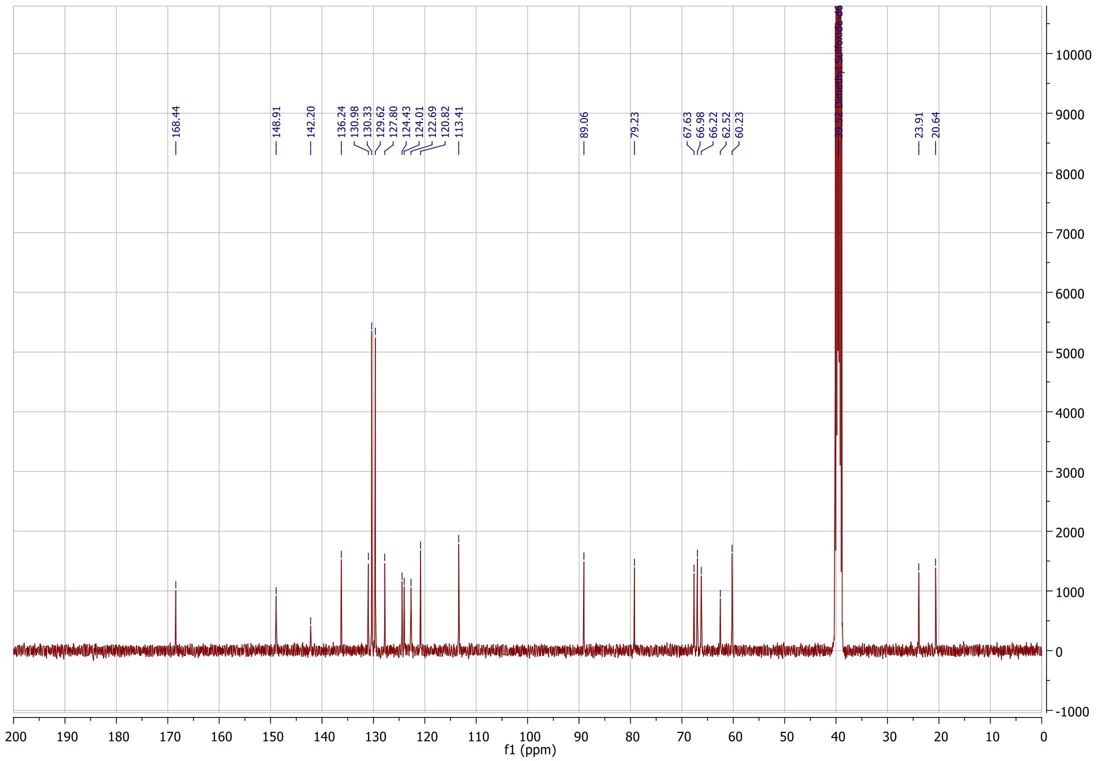

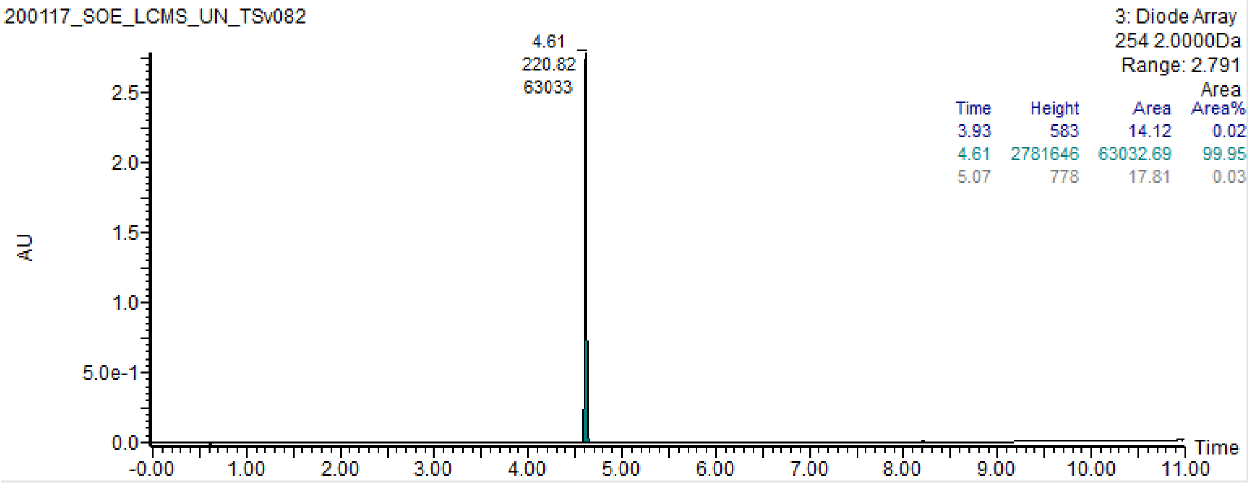
200117_SOE_LCMS_UN_TSv082
3: Diode Array
254 2.0000Da
Range: 2.791
Area
Time Height Area Area%
3.93 583 14.12 0.02
4.61 2781646 63032.69 99.95
5.07 778 17.81 0.03
4.61
220.82
63033
AU
2.5
2.0
1.5
1.0
5.0e-1
0.0
-0.00
1.00
2.00
3.00
4.00
5.00
6.00
7.00
8.00
9.00
10.00
11.00
Time

Tolyl 3-(4-((methylsulfonamidophenyloxy)methyl)-1,2,3-triazol-1-yl)-3-deoxy-1-thio-β-D-galactopyranoside (37)

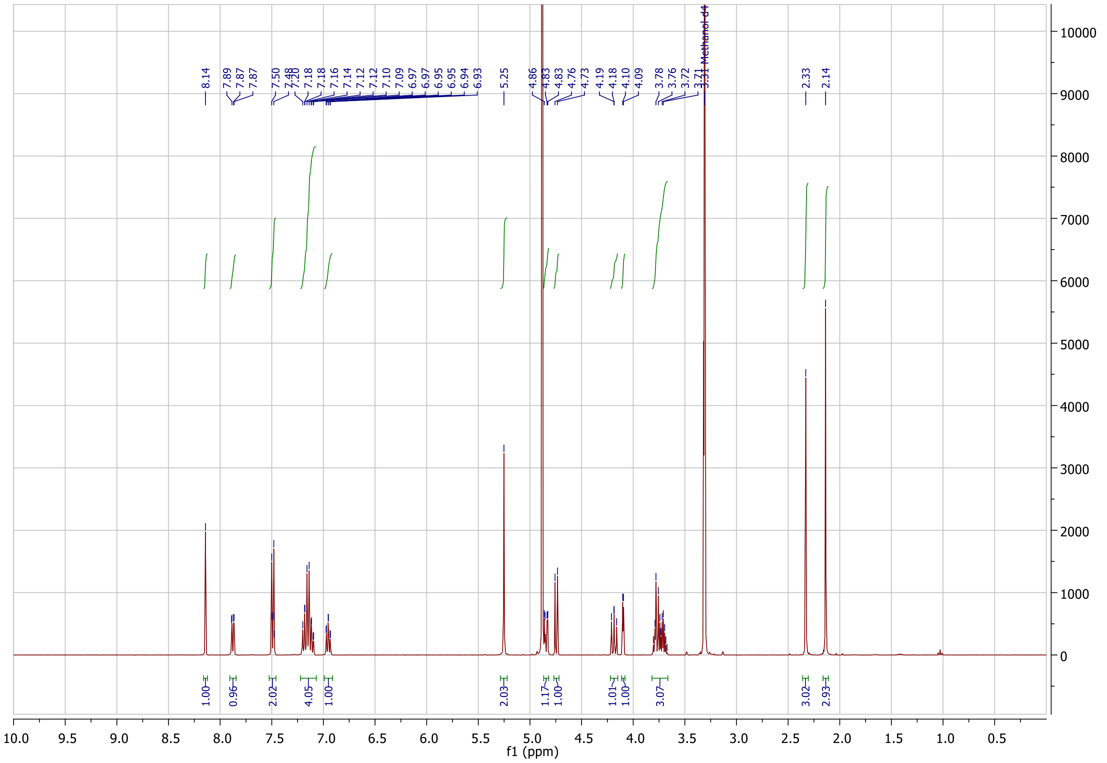

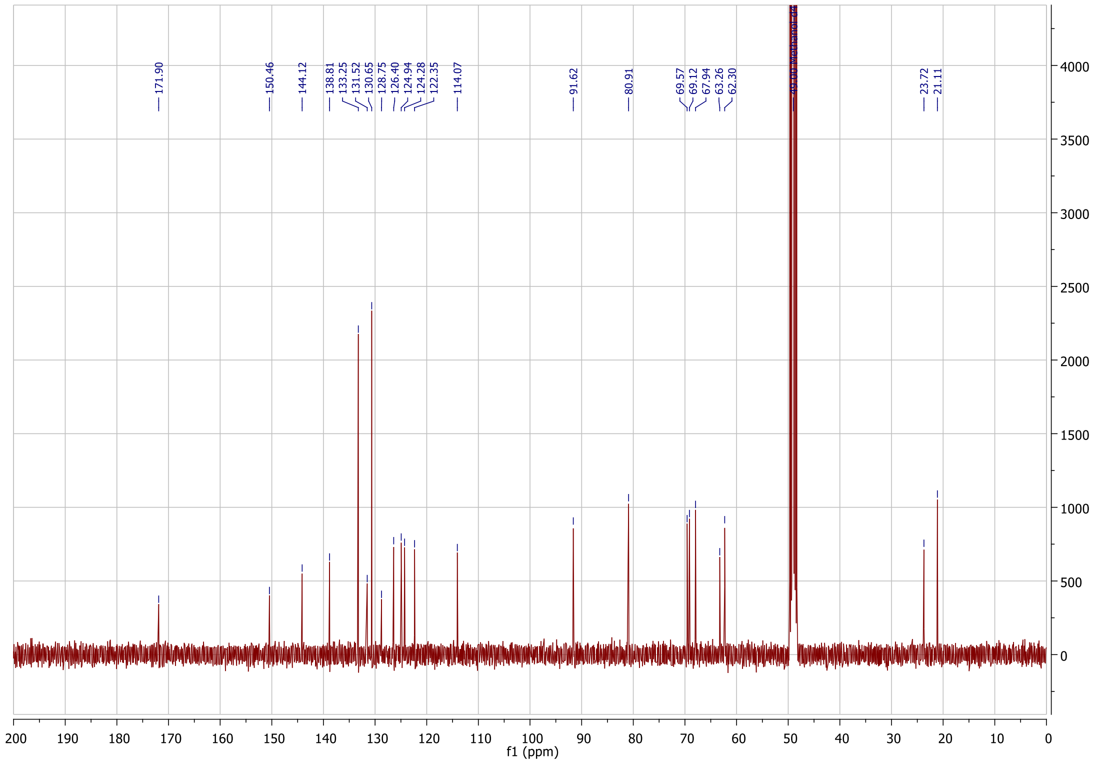

Chromatogram

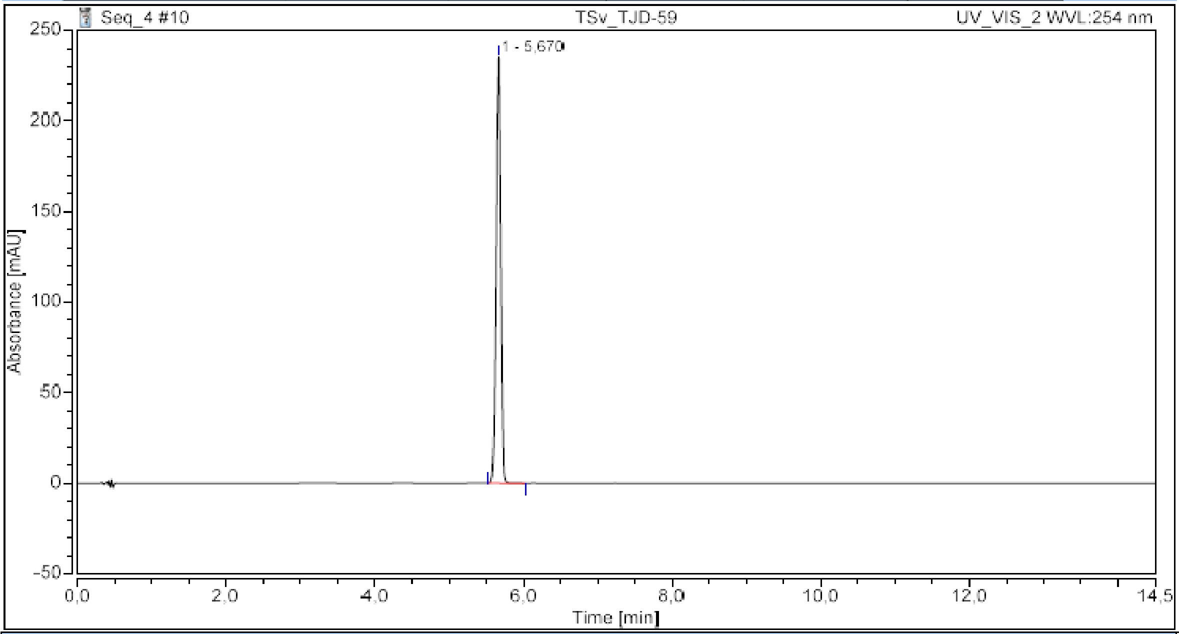

Integration Results

No.	Peak Name	Retention Time min	Area mAU*min	Height mAU	Relative Area %	Relative Height %	Amount n.a.
1		5,670	17,159	235,461	100,00	100,00	n.a.
**Total:**			**17,159**	**235,461**	**100,00**	**100,00**	

Tolyl 3-(4-((3-methoxycarbonylphenyloxy)methyl)-1,2,3-triazol-1-yl)-3-deoxy-1-thio-β-D-galactopyranoside (38)

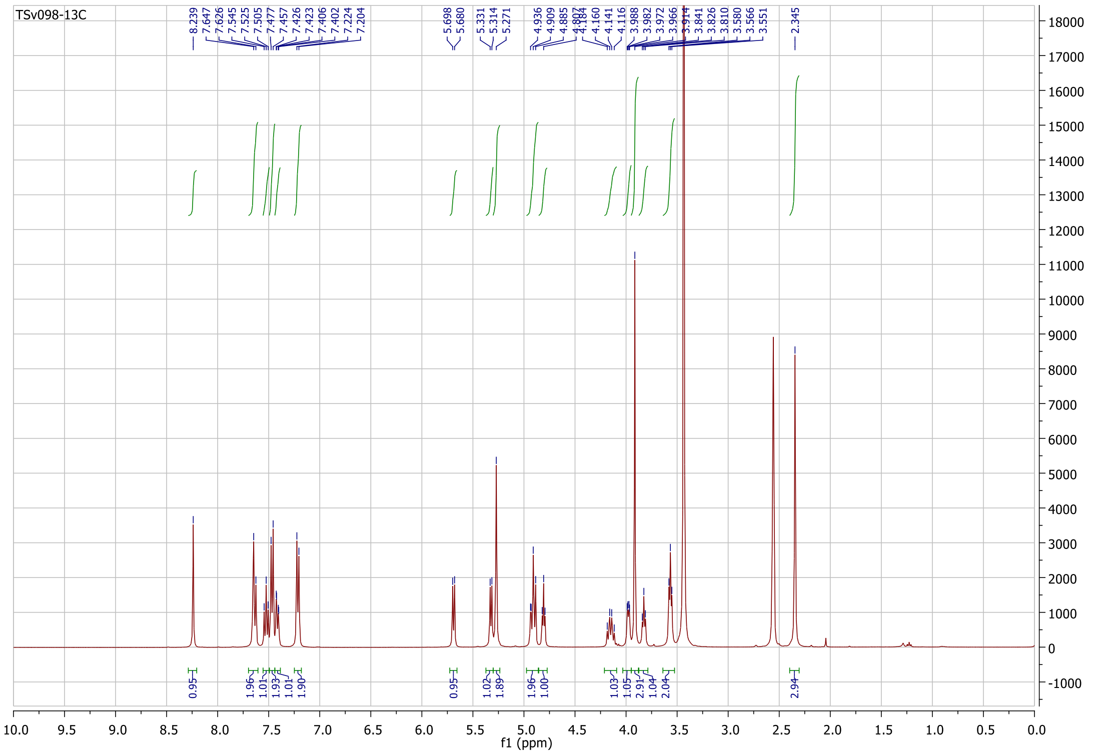

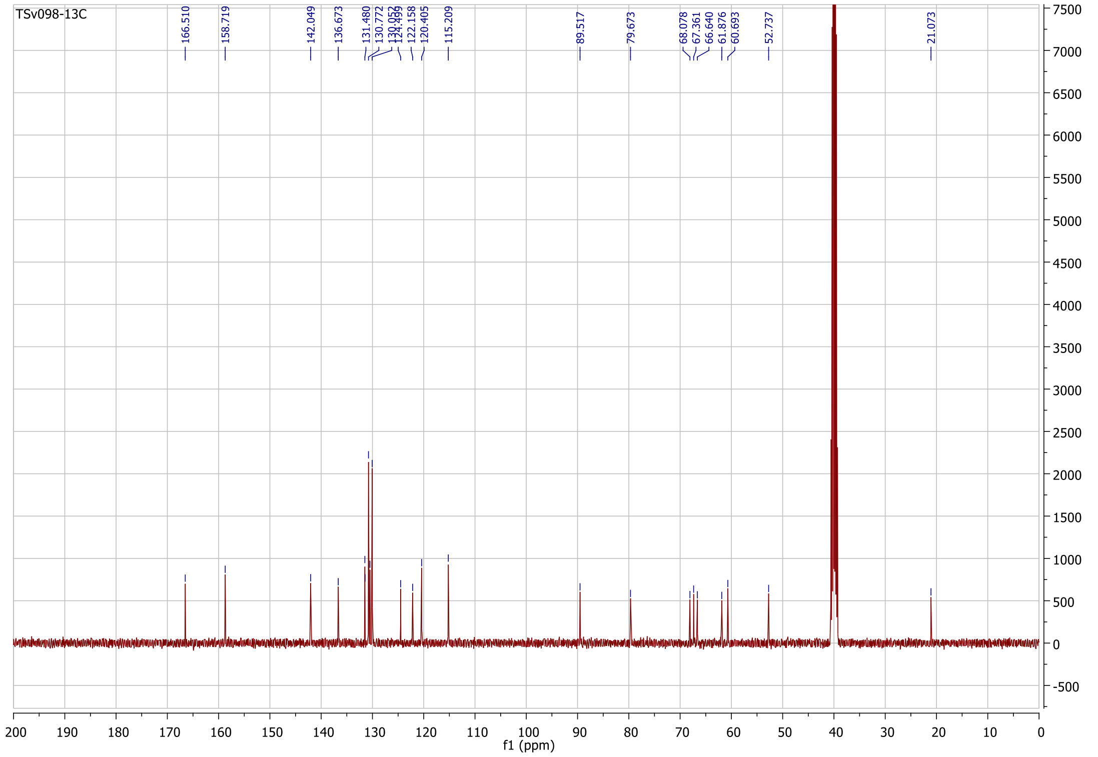

Chromatogram

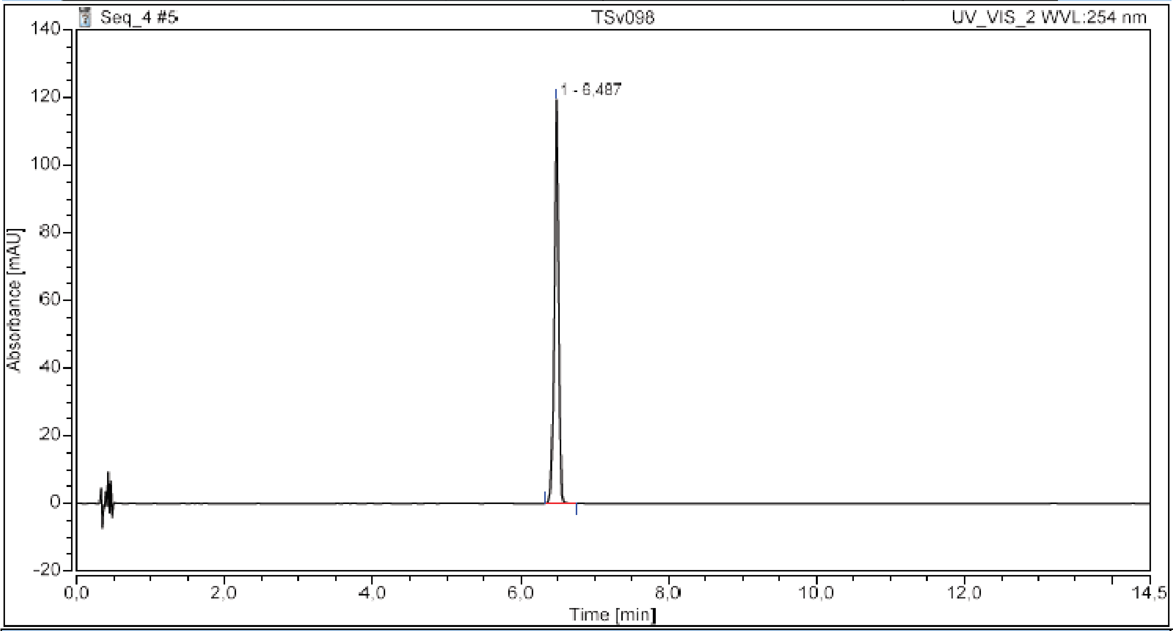

Integration Results

No.	Peak Name	Retention Time min	Area mAU*min	Height mAU	Relative Area %	Relative Height %	Amount n.a.
1		6,487	8,042	119,387	100,00	100,00	n.a.
**Total:**			**8,042**	**119,387**	**100,00**	**100,00**	

Tolyl 3-(4-((3-carboxyphenyloxy)methyl)-1,2,3-triazol-1-yl)-3-deoxy-1-thio-β-D-galactopyranoside (39)

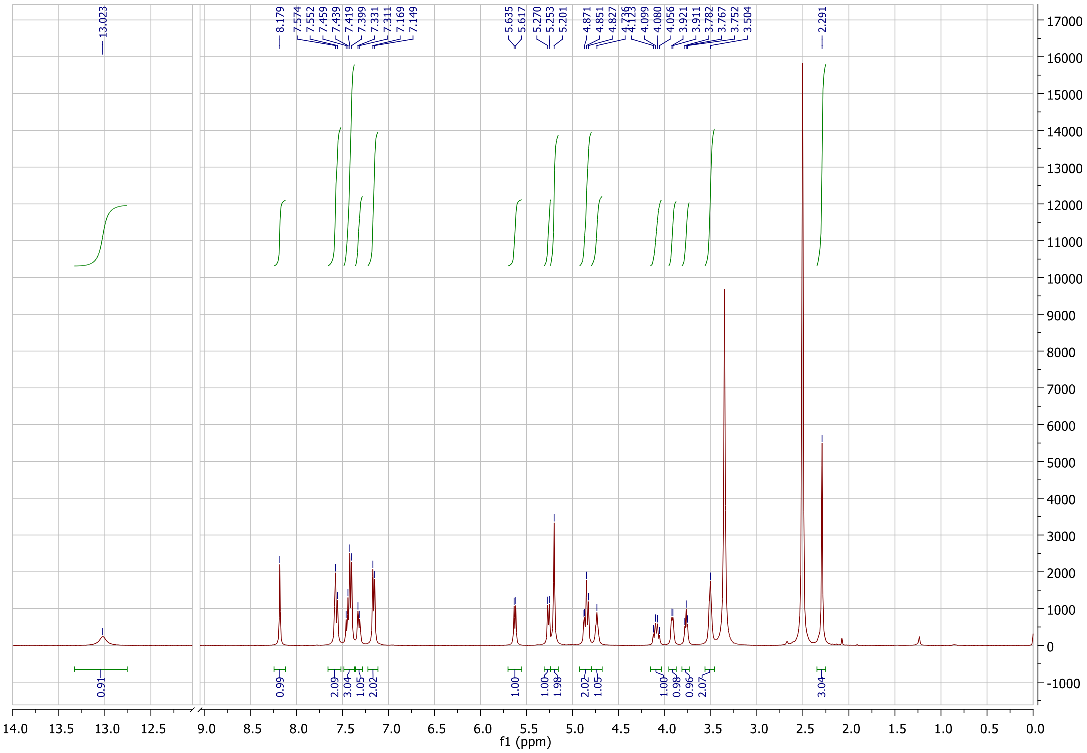

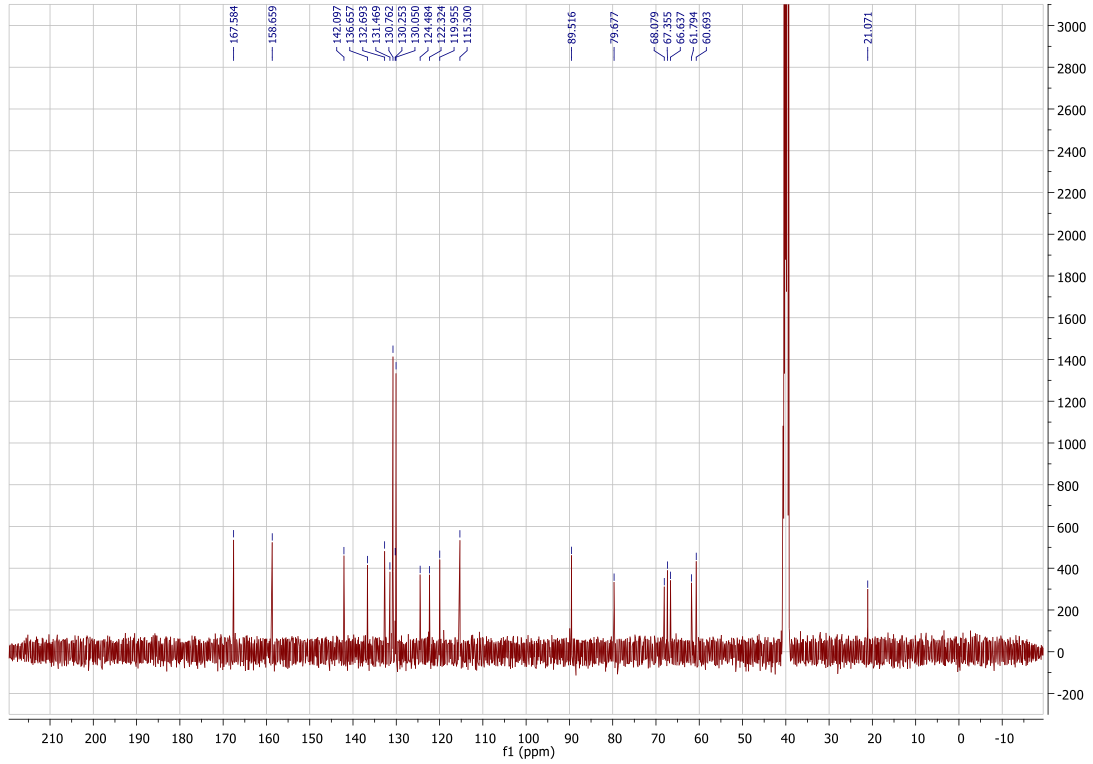

Chromatogram

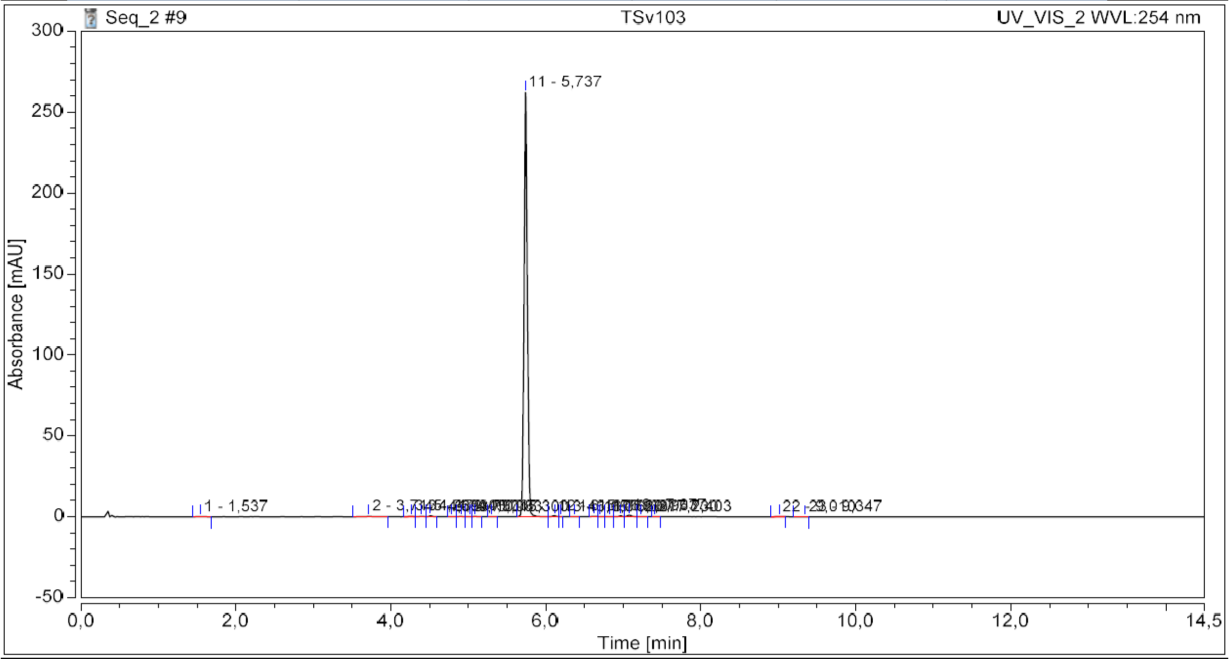

Integration Results

No.	Peak Name	Retention Time min	Area mAU*min	Height mAU	Relative Area %	Relative Height %	Amount n.a.
1		1,537	0,004	0,043	0,03	0,02	n.a.
2		3,713	0,025	0,257	0,19	0,10	n.a.
3		4,260	0,018	0,310	0,14	0,12	n.a.
4		4,393	0,019	0,228	0,14	0,09	n.a.
5		4,510	0,026	0,517	0,19	0,19	n.a.
6		4,777	0,002	0,028	0,01	0,01	n.a.
7		4,903	0,013	0,289	0,10	0,11	n.a.
8		5,017	0,006	0,080	0,04	0,03	n.a.
9		5,083	0,010	0,209	0,08	0,08	n.a.
10		5,300	0,005	0,092	0,03	0,03	n.a.
11		5,737	13,286	262,133	97,88	98,17	n.a.
12		6,110	0,031	0,502	0,23	0,19	n.a.
13		6,187	0,002	0,059	0,01	0,02	n.a.
14		6,370	0,002	0,035	0,02	0,01	n.a.
15		6,620	0,017	0,310	0,12	0,12	n.a.
16		6,697	0,005	0,081	0,04	0,03	n.a.
17		6,817	0,007	0,131	0,05	0,05	n.a.
18		6,963	0,025	0,457	0,18	0,17	n.a.
19		7,077	0,052	0,944	0,39	0,35	n.a.
20		7,230	0,004	0,066	0,03	0,02	n.a.
21		7,403	0,002	0,037	0,02	0,01	n.a.
22		9,010	0,010	0,167	0,08	0,06	n.a.
23		9,347	0,003	0,032	0,02	0,01	n.a.
**Total:**			**13,573**	**267,007**	**100,00**	**100,00**	

Tolyl 3-(4-((2-amino-5-methoxycarbonyl-phenyloxy)methyl)-1,2,3-triazol-1-yl)-3-deoxy-1-thio-β-D-galactopyranoside (40)

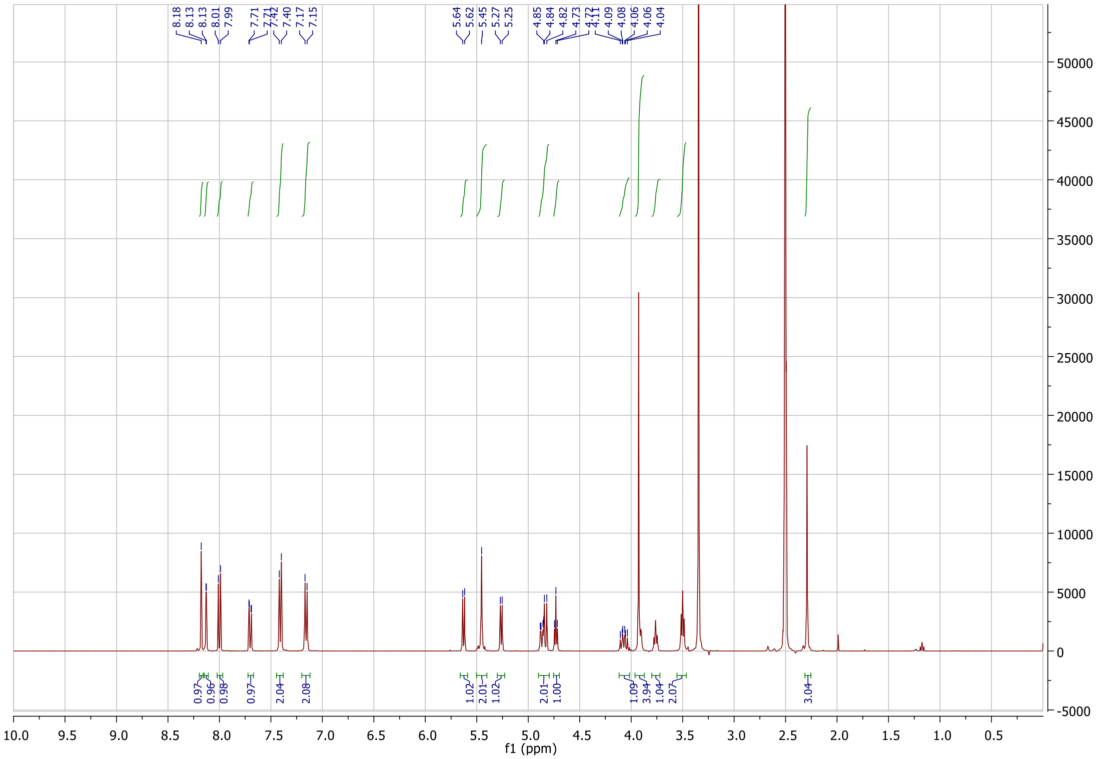

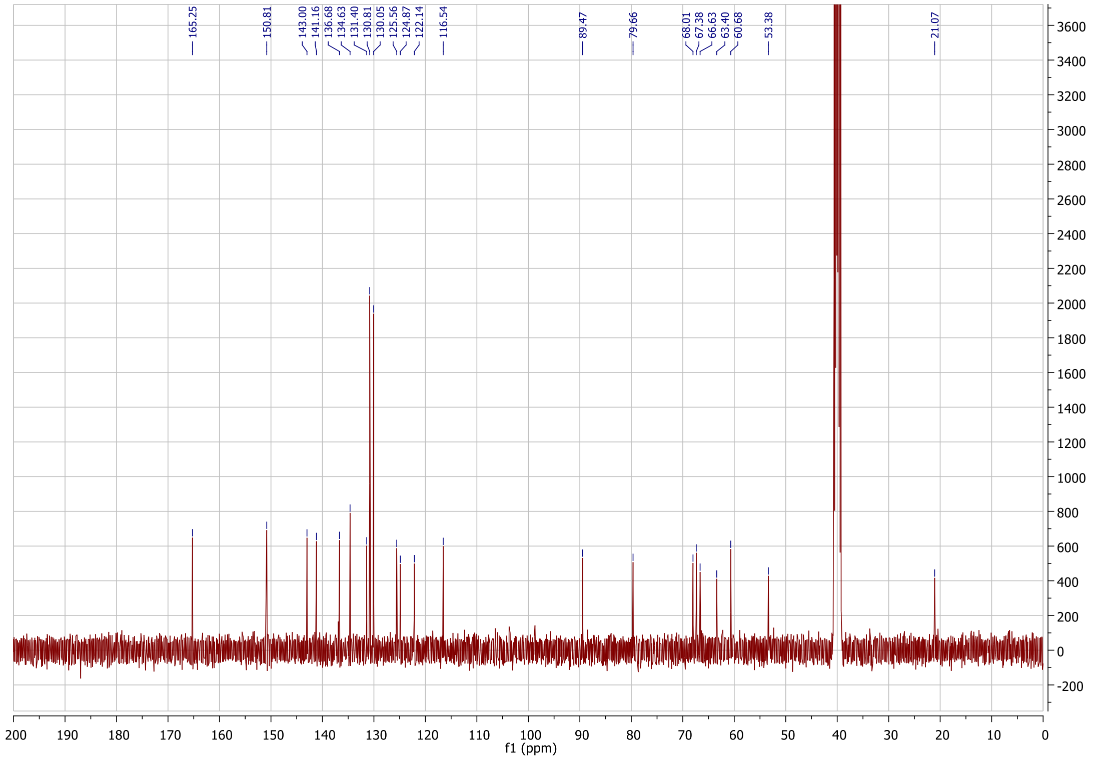

Chromatogram

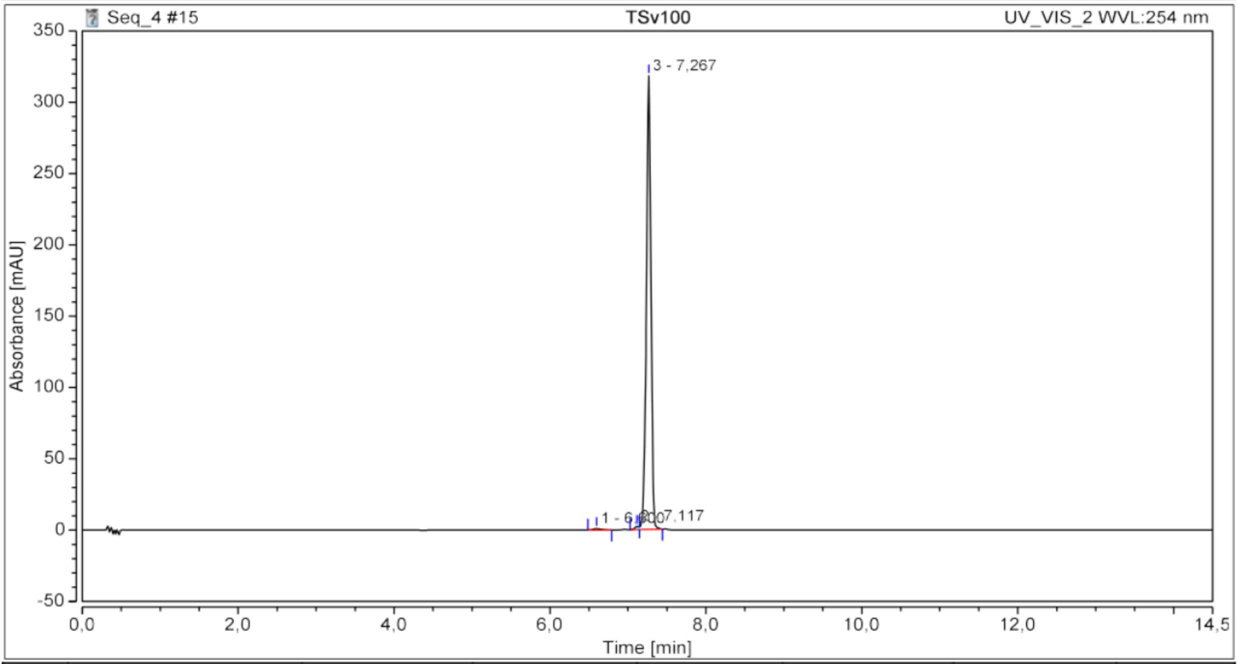

Integration Results

No.	Peak Name	Retention Time<br>min	Area<br>mAU*min	Height<br>mAU	Relative Area<br>%	Relative Height<br>%	Amount<br>n.a.
1		6,600	0,108	1,039	0,49	0,32	n.a.
2		7,117	0,144	2,386	0,66	0,74	n.a.
3		7,267	21,603	317,974	98,85	98,93	n.a.
**Total:**			**21,855**	**321,399**	**100,00**	**100,00**	

Tolyl 3-(4-((2-amino-5-carboxy-phenyloxy)methyl)-1,2,3-triazol-1-yl)-3-deoxy-1-thio-β-D-galactopyranoside (41)

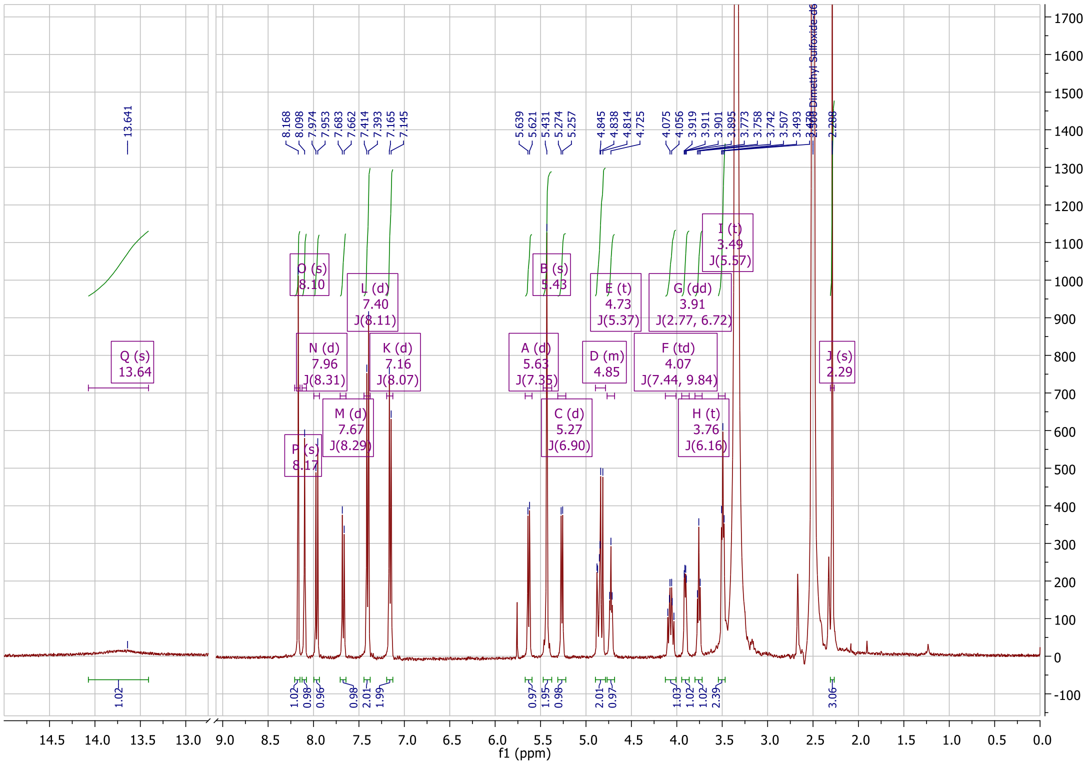

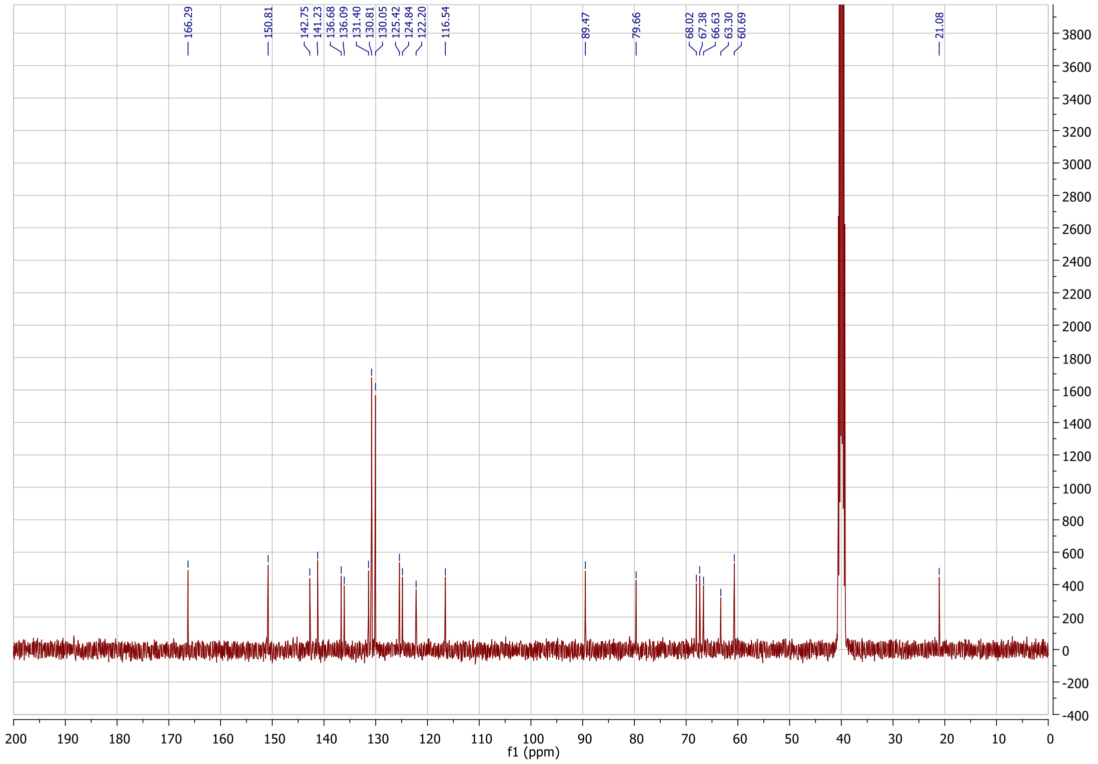

Chromatogram

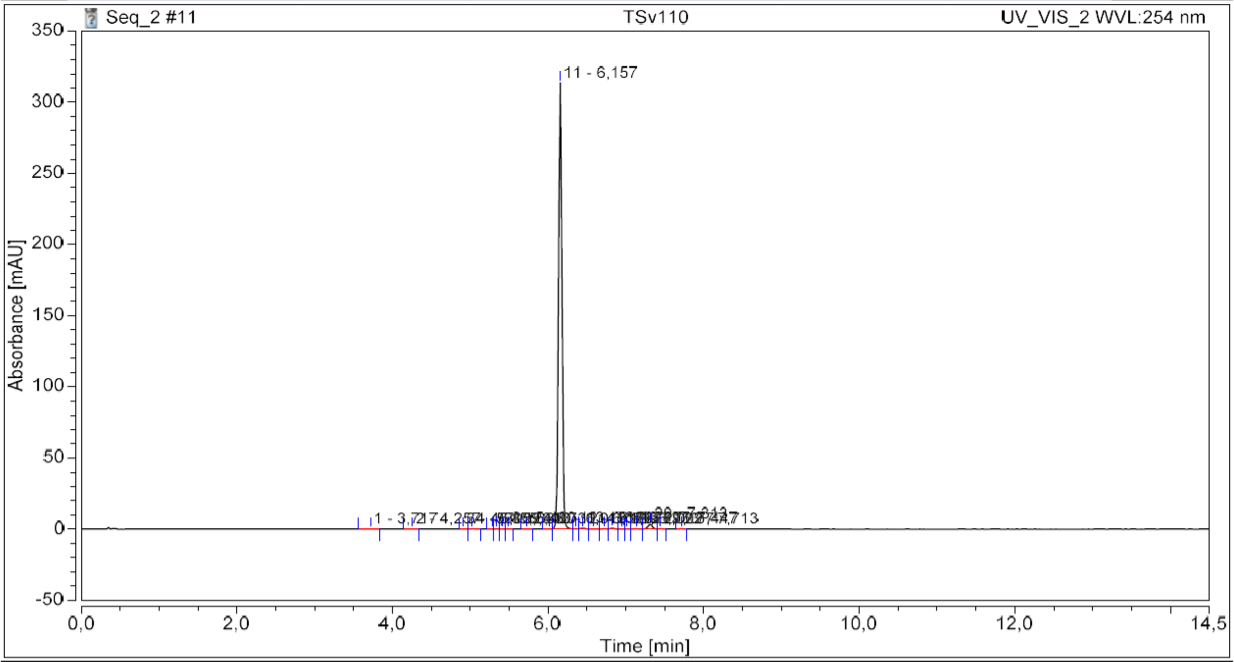

Integration Results

No.	Peak Name	Retention Time<br>min	Area<br>mAU*min	Height<br>mAU	Relative Area<br>%	Relative Height<br>%	Amount<br>n.a.
1		3,717	0,008	0,095	0,05	0,03	n.a.
2		4,257	0,010	0,155	0,06	0,05	n.a.
3		4,910	0,006	0,138	0,03	0,04	n.a.
4		5,027	0,014	0,230	0,08	0,07	n.a.
5		5,280	0,005	0,107	0,03	0,03	n.a.
6		5,333	0,011	0,220	0,06	0,07	n.a.
7		5,413	0,007	0,150	0,04	0,05	n.a.
8		5,480	0,013	0,260	0,08	0,08	n.a.
9		5,730	0,006	0,086	0,04	0,03	n.a.
10		6,013	0,011	0,195	0,06	0,06	n.a.
11		6,157	16,457	313,636	96,97	97,40	n.a.
12		6,353	0,020	0,343	0,11	0,11	n.a.
13		6,447	0,045	0,718	0,27	0,22	n.a.
14		6,587	0,011	0,124	0,07	0,04	n.a.
15		6,727	0,015	0,215	0,09	0,07	n.a.
16		6,827	0,043	0,792	0,26	0,25	n.a.
17		6,953	0,007	0,111	0,04	0,03	n.a.
18		7,007	0,007	0,106	0,04	0,03	n.a.
19		7,137	0,014	0,167	0,08	0,05	n.a.
20		7,313	0,232	3,646	1,37	1,13	n.a.
21		7,447	0,018	0,325	0,10	0,10	n.a.
22		7,713	0,011	0,206	0,06	0,06	n.a.
**Total:**			**16,971**	**322,024**	**100,00**	**100,00**	

Tolyl 3-(4-((5-methoxycarbonyl-2-nitrophenyloxy)methyl)-1,2,3-triazol-1-yl)-3-deoxy-1-thio-β-D-galactopyranoside (42)

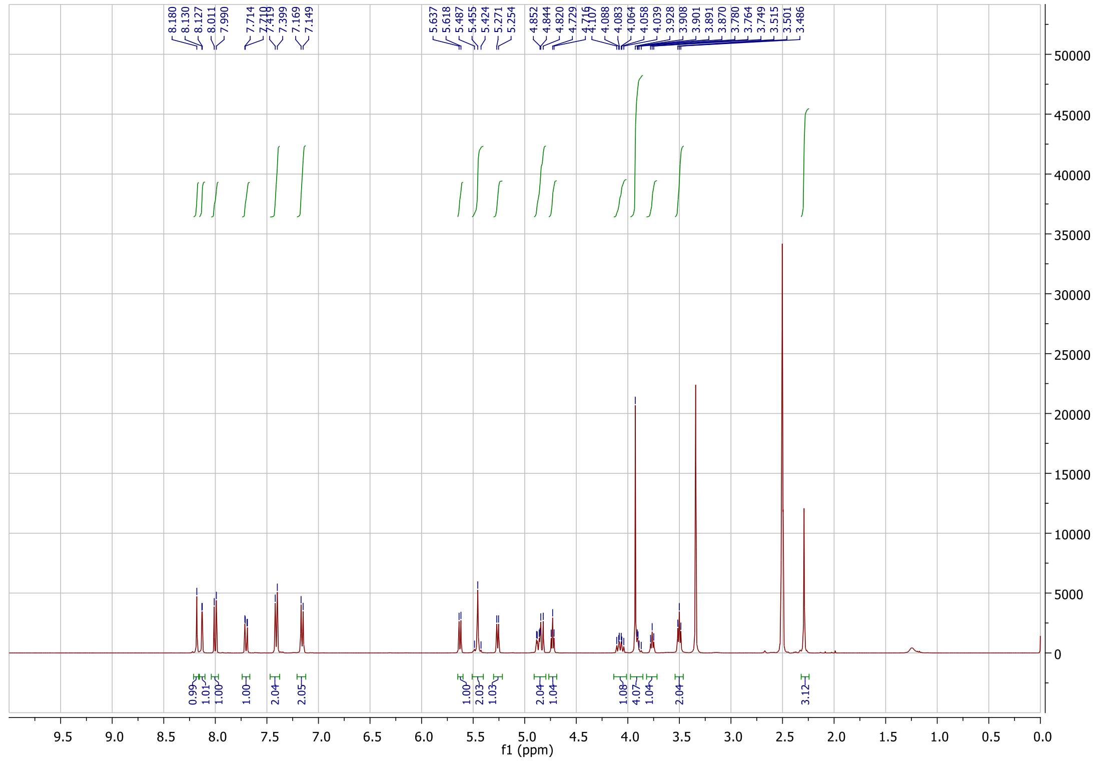

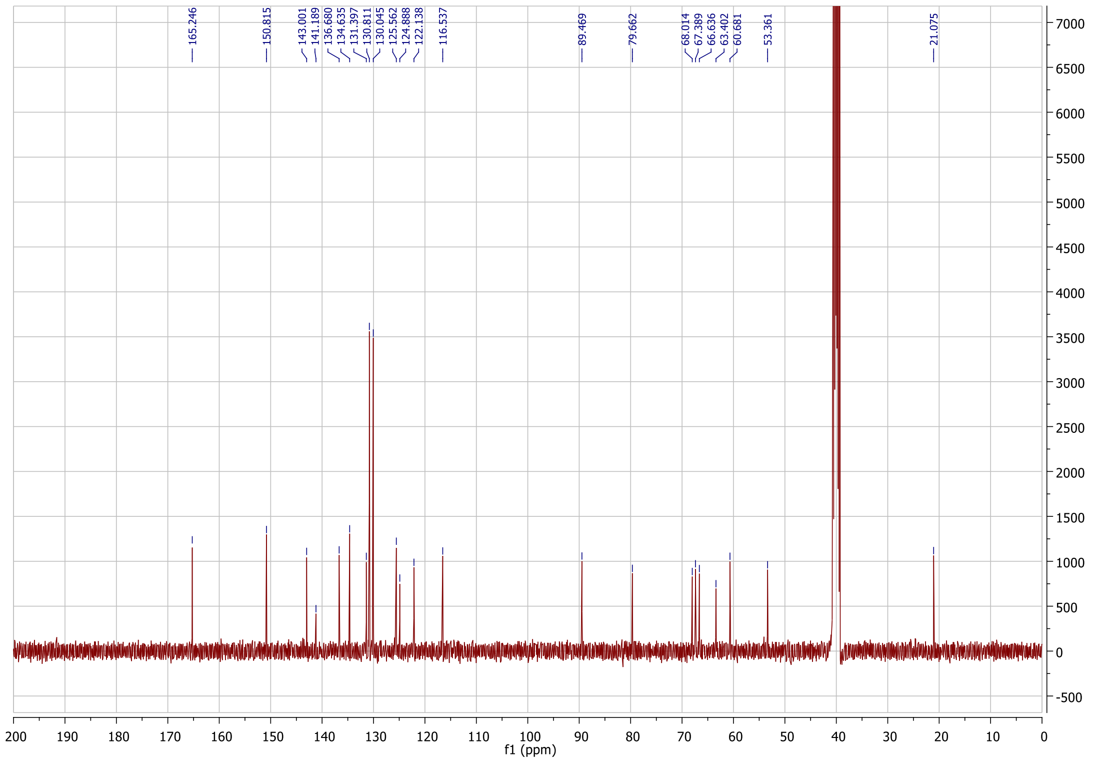

Chromatogram

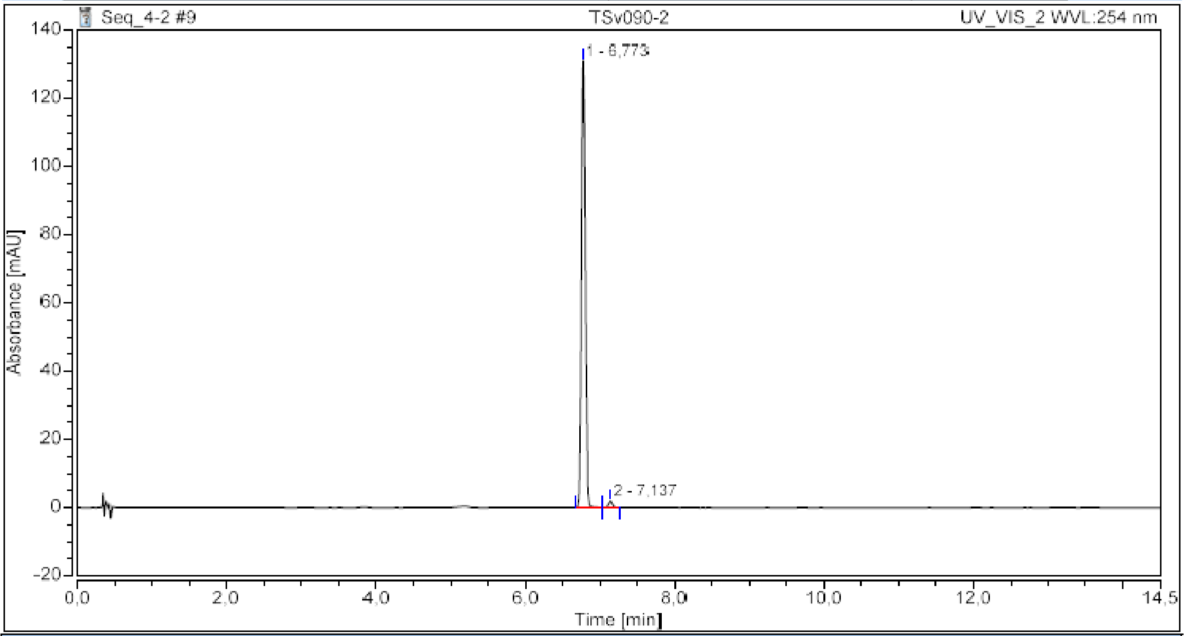

Integration Results

No.	Peak Name	Retention Time min	Area mAU*min	Height mAU	Relative Area %	Relative Height %	Amount n.a.
1		6,773	7,561	131,023	98,59	98,53	n.a.
2		7,137	0,108	1,950	1,41	1,47	n.a.
**Total:**			**7,669**	**132,973**	**100,00**	**100,00**	

Tolyl 3-(4-((5-carboxy-2-nitrophenyloxy)methyl)-1,2,3-triazol-1-yl)-3-deoxy-1-thio-β-D-galactopyranoside (43)

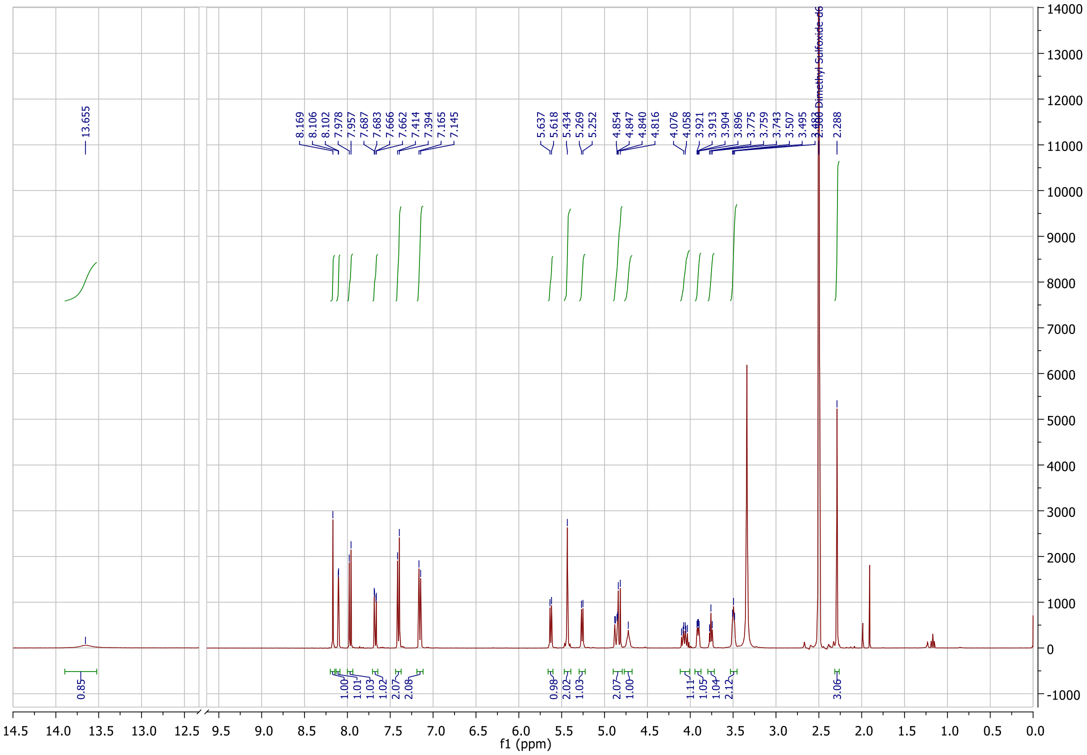

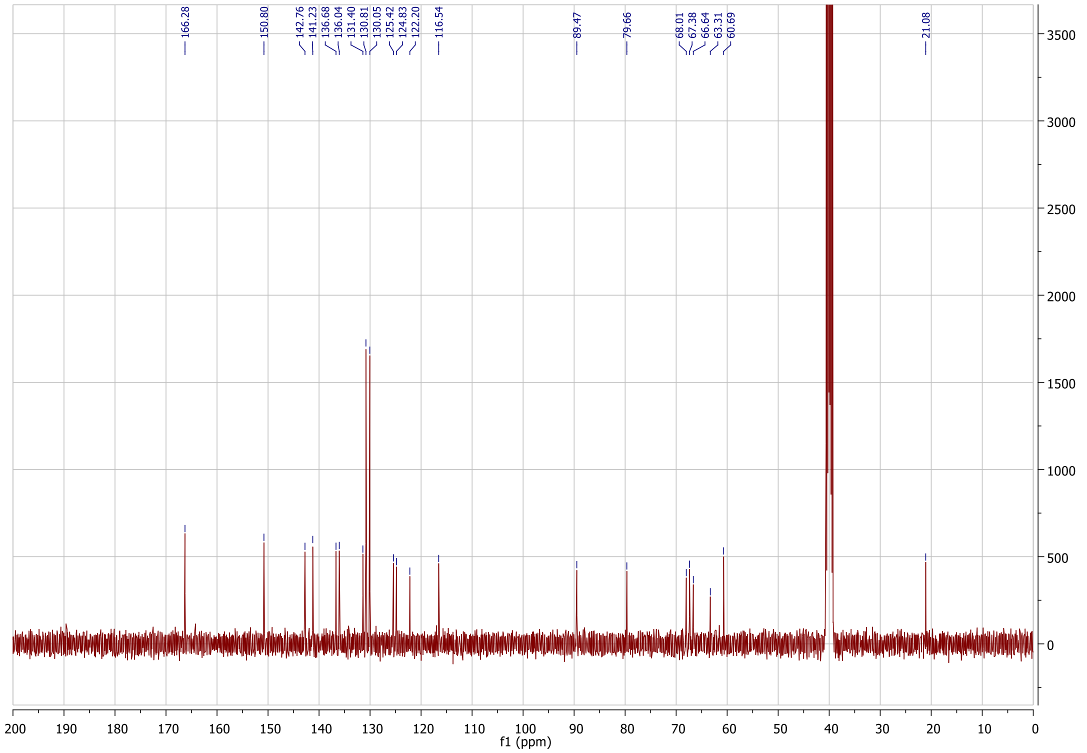

Chromatogram

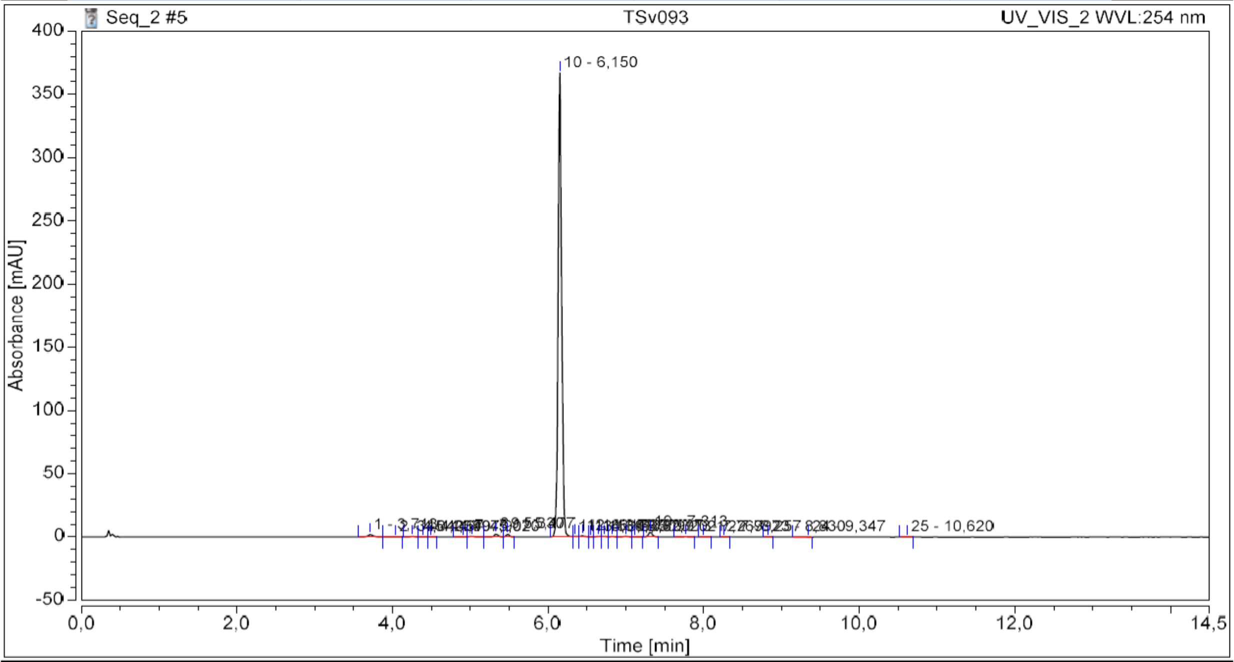

Integration Results

No.	Peak Name	Retention Time min	Area mAU*min	Height mAU	Relative Area %	Relative Height %	Amount n.a.
1		3,713	0,146	1,828	0,73	0,48	n.a.
2		4,040	0,006	0,066	0,03	0,02	n.a.
3		4,257	0,015	0,235	0,07	0,06	n.a.
4		4,390	0,009	0,122	0,05	0,03	n.a.
5		4,497	0,004	0,069	0,02	0,02	n.a.
6		4,907	0,007	0,113	0,04	0,03	n.a.
7		5,020	0,024	0,350	0,12	0,09	n.a.
8		5,330	0,119	2,415	0,59	0,63	n.a.
9		5,477	0,103	2,193	0,52	0,58	n.a.
10		6,150	19,237	366,913	95,85	96,25	n.a.
11		6,347	0,022	0,366	0,11	0,10	n.a.
12		6,443	0,048	0,843	0,24	0,22	n.a.
13		6,543	0,006	0,113	0,03	0,03	n.a.
14		6,650	0,017	0,239	0,08	0,06	n.a.
15		6,720	0,013	0,201	0,07	0,05	n.a.
16		6,823	0,015	0,290	0,07	0,08	n.a.
17		6,997	0,026	0,389	0,13	0,10	n.a.
18		7,113	0,004	0,064	0,02	0,02	n.a.
19		7,313	0,208	3,916	1,04	1,03	n.a.
20		7,767	0,018	0,196	0,09	0,05	n.a.
21		7,997	0,004	0,070	0,02	0,02	n.a.
22		8,257	0,003	0,044	0,01	0,01	n.a.
23		8,830	0,004	0,064	0,02	0,02	n.a.
24		9,347	0,007	0,071	0,04	0,02	n.a.
25		10,620	0,004	0,051	0,02	0,01	n.a.
**Total:**			**20,070**	**381,220**	**100,00**	**100,00**	

Tolyl 3-(4-((2-acetamido-5-methoxycarbonyl-phenyloxy)methyl)-1,2,3-triazol-1-yl)-3-deoxy-1-thio-β-D-galactopyranoside (44)

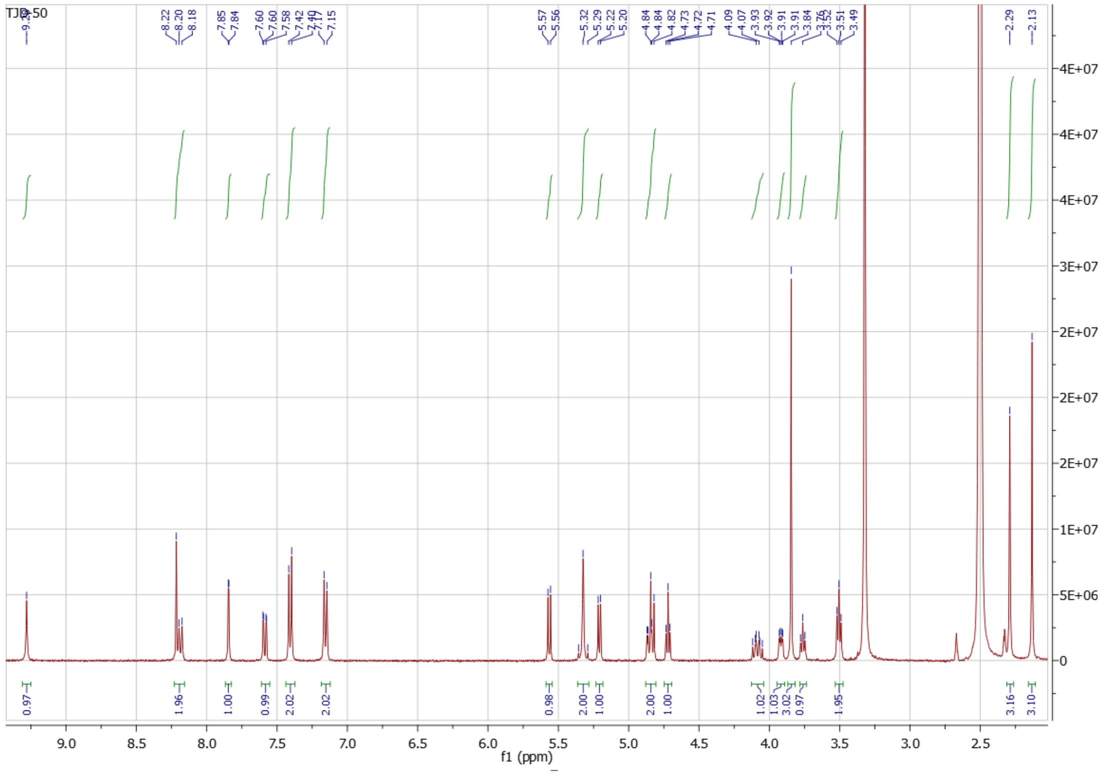

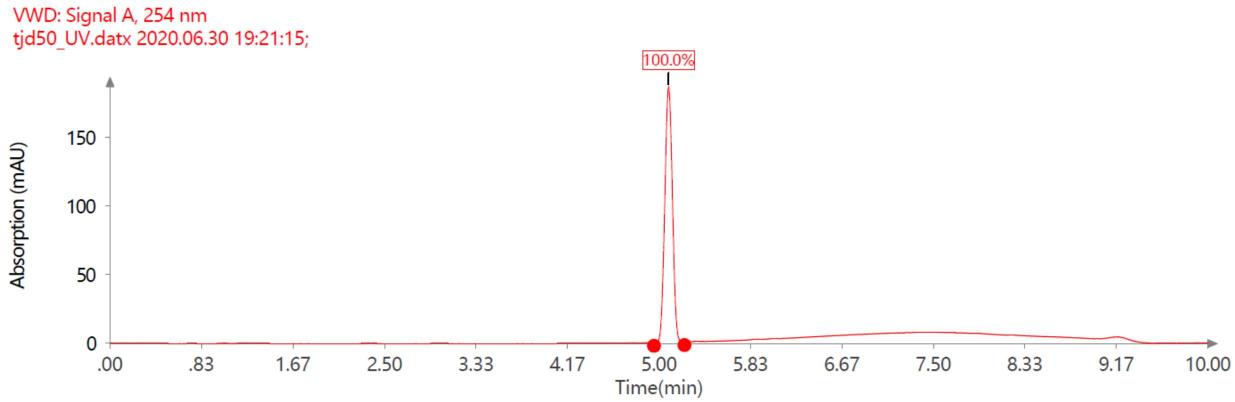

Time (Peak Maximum M:S/Minutes)	Maximum Intensity (c/s)	Time (Peak Centroid M:S/Minutes)	Peak Area	% Peak Area	Peak Resolution	Label
5.09	1.9E2	5.09	9.3E2	100.0	4.6	

Tolyl 3-(4-(phenoxymethyl)-1,2,3-triazol-1-yl)-3-deoxy-1-thio-β-D-galactopyranoside (45)

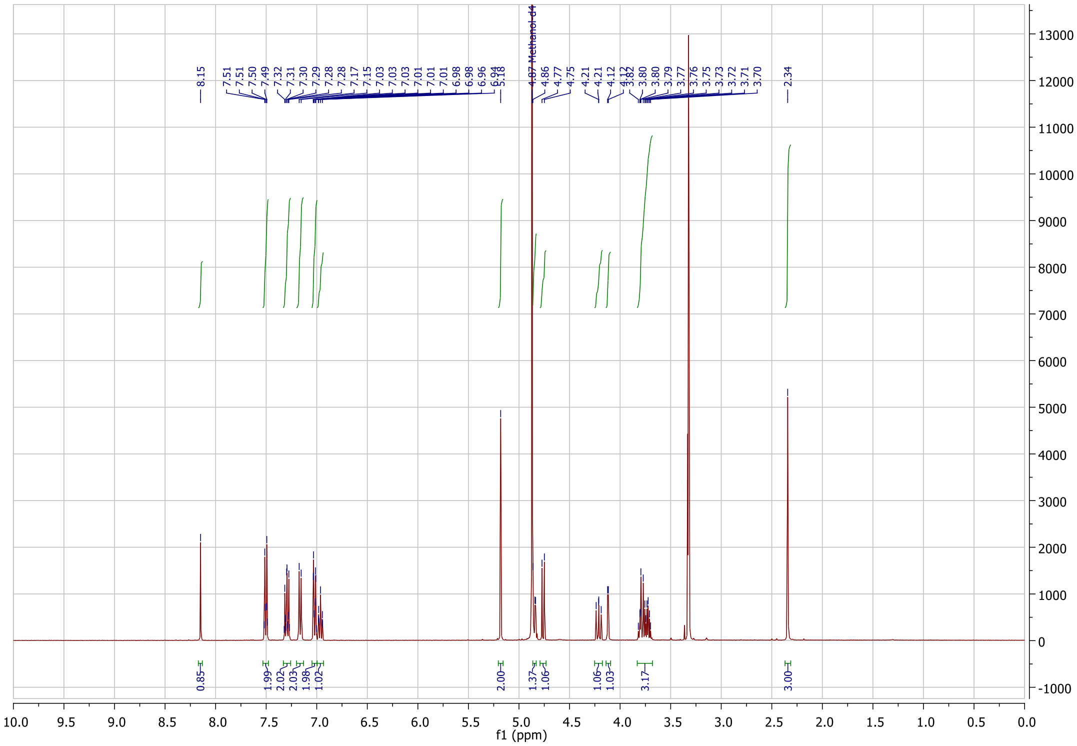

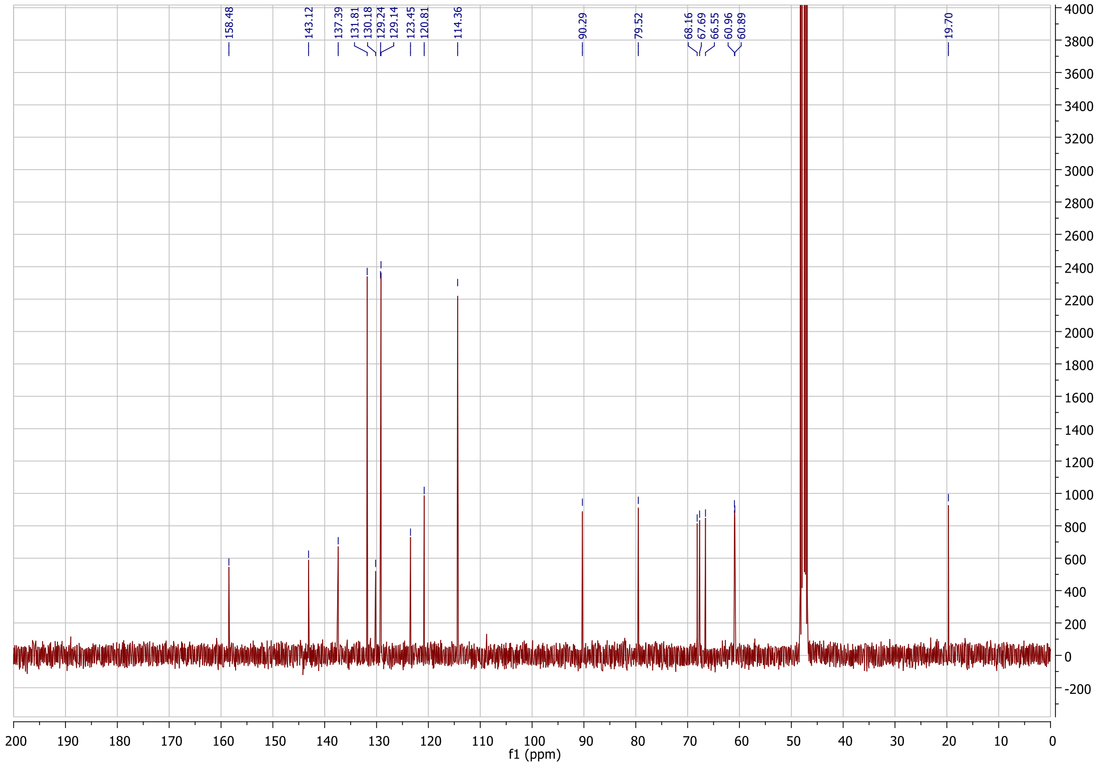

Chromatogram

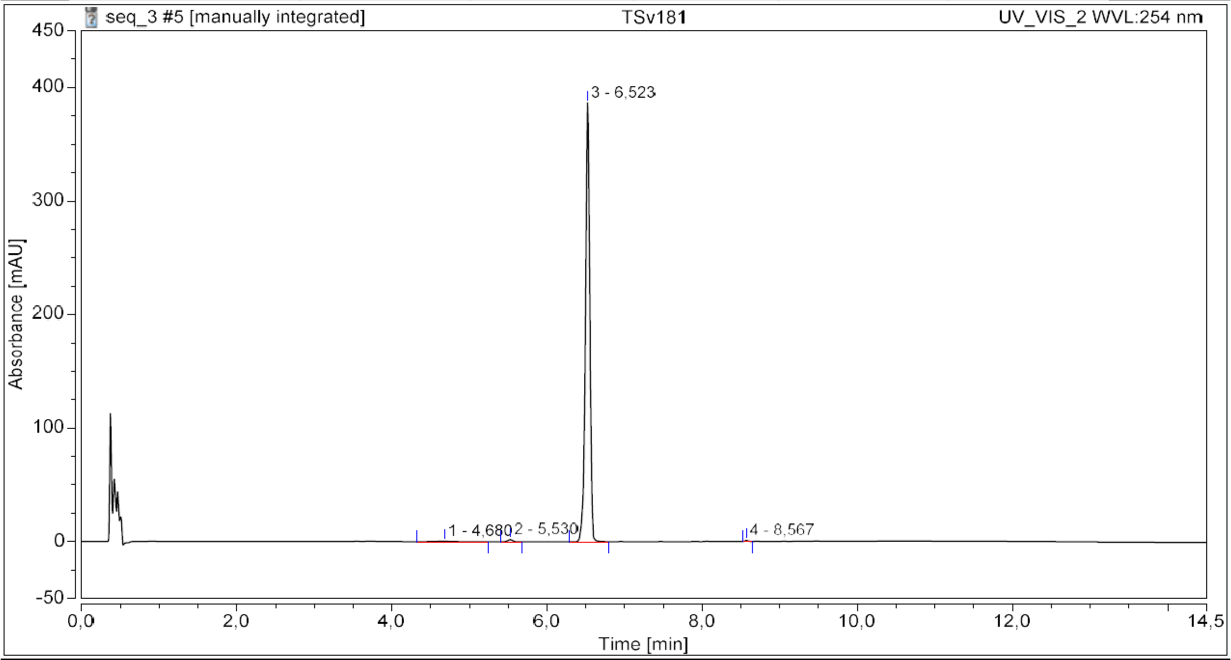

Integration Results

No.	Peak Name	Retention Time min	Area mAU*min	Height mAU	Relative Area %	Relative Height %	Amount n.a.
1		4,680	0,104	0,413	0,41	0,11	n.a.
2		5,530	0,148	2,108	0,58	0,54	n.a.
3		6,523	25,083	386,843	98,84	99,15	n.a.
4		8,567	0,042	0,815	0,16	0,21	n.a.
**Total:**			**25,377**	**390,178**	**100,00**	**100,00**	

Tolyl 3-(4-((2-hydroxyphenyl)methyl)-1,2,3-triazol-1-yl)-3-deoxy-1-thio-β-D-galactopyranoside (46)

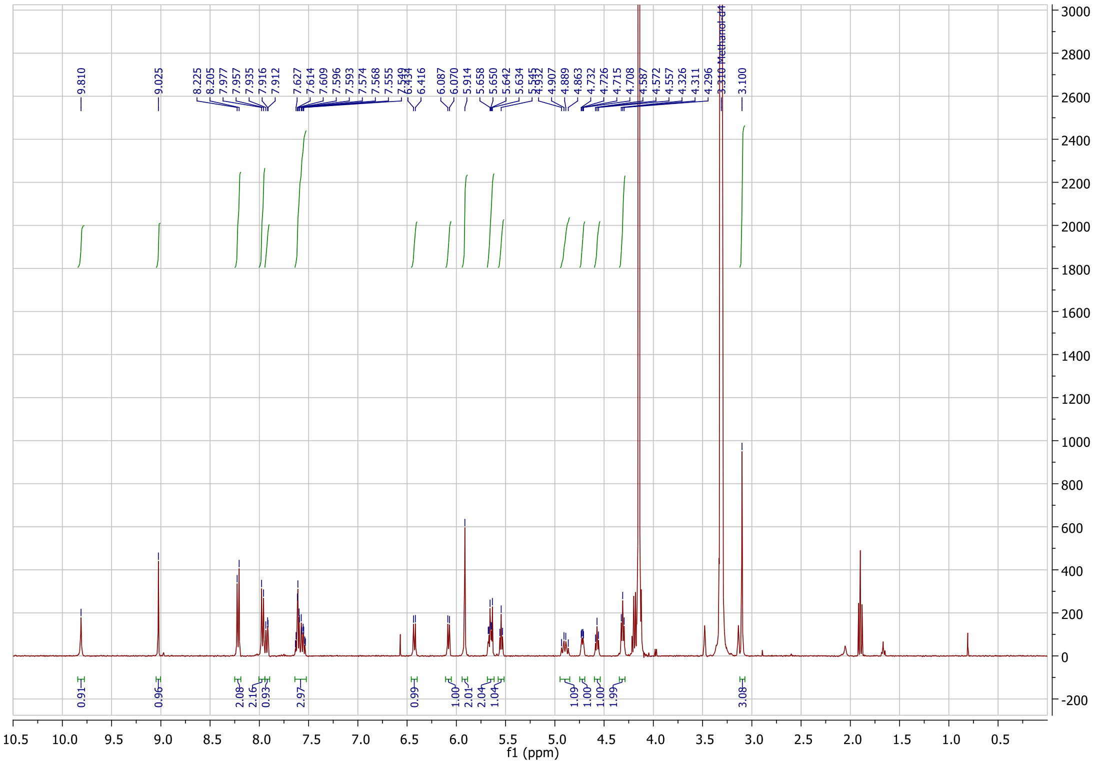

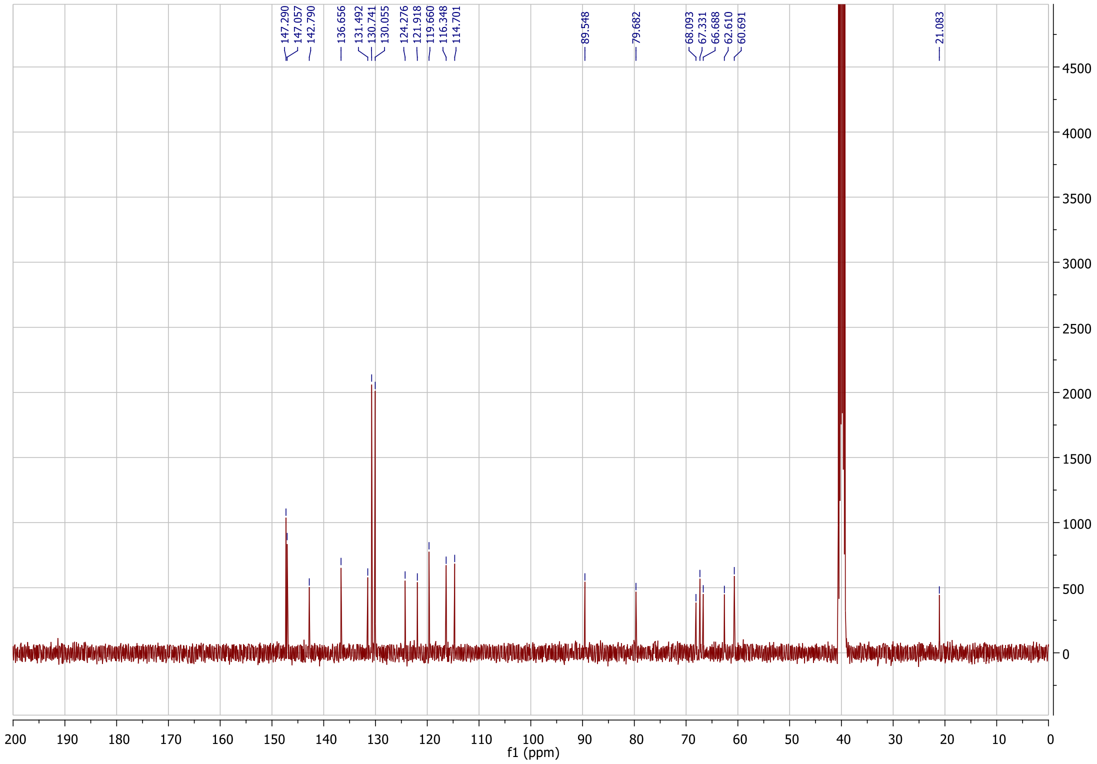

Chromatogram

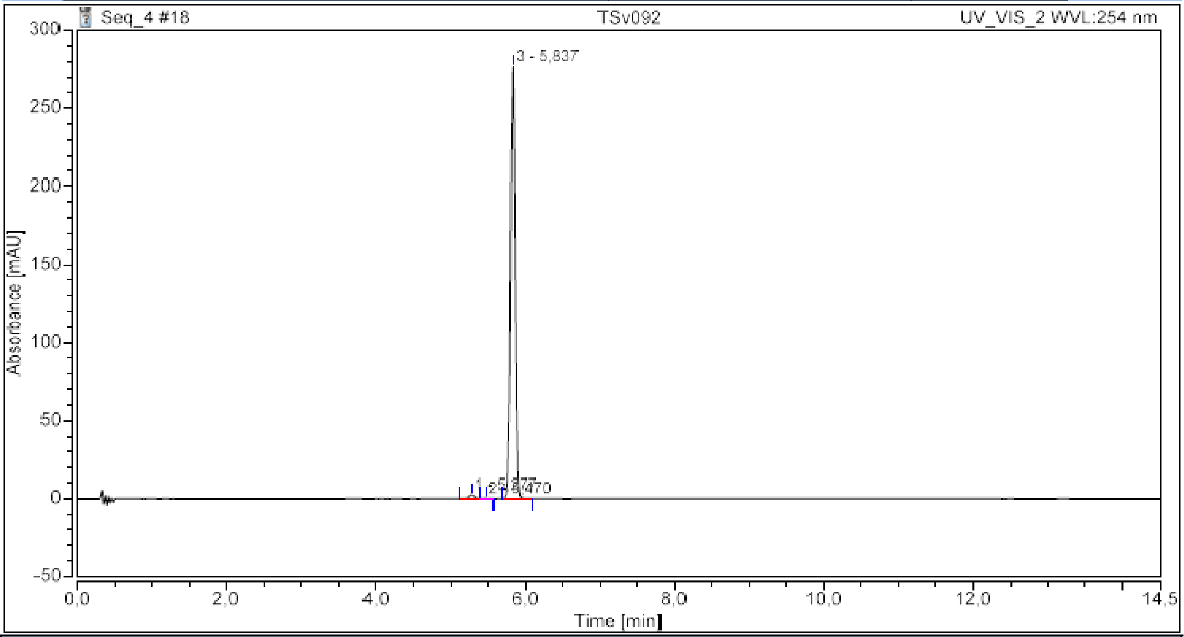

Integration Results

No.	Peak Name	Retention Time min	Area mAU*min	Height mAU	Relative Area %	Relative Height %	Amount n.a.
1		5,277	0,197	2,503	0,97	0,90	n.a.
2		5,470	0,020	0,302	0,10	0,11	n.a.
3		5,837	20,105	276,760	98,93	99,00	n.a.
**Total:**			**20,323**	**279,565**	**100,00**	**100,00**	

Tolyl 3-(4-((2-(hydroxymethyl)phenyl)methyl)-1,2,3-triazol-1-yl)-3-deoxy-1-thio-β-D-galactopyranoside (47)

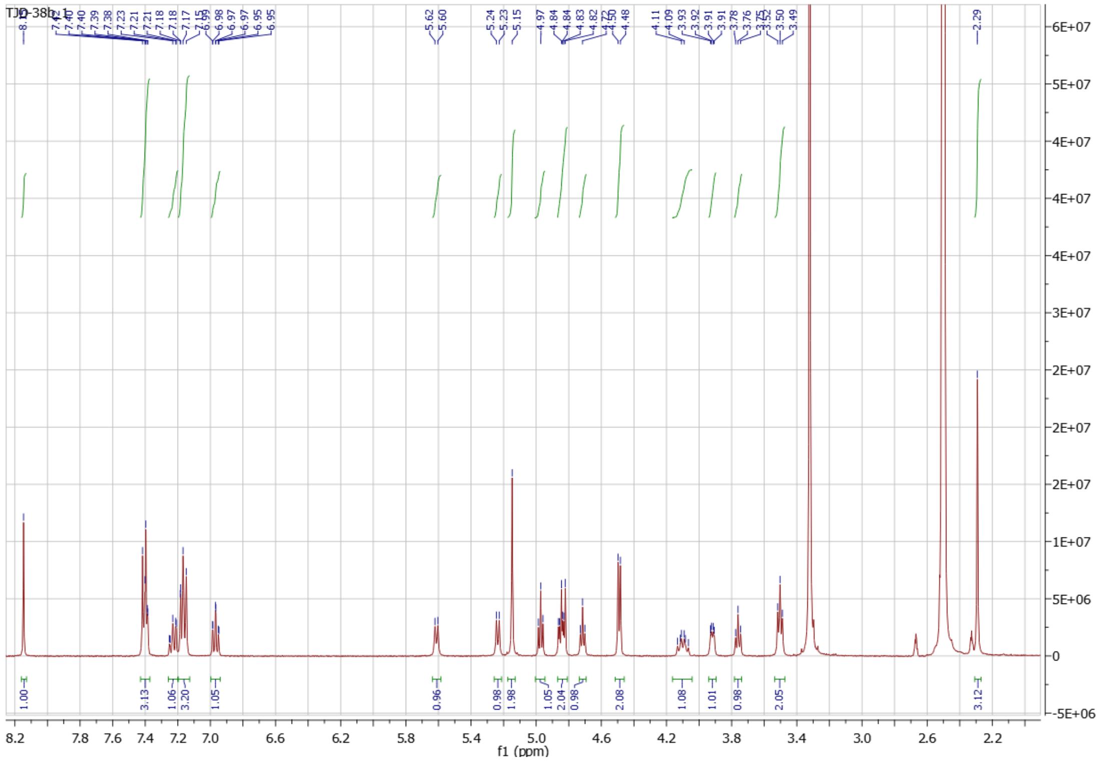

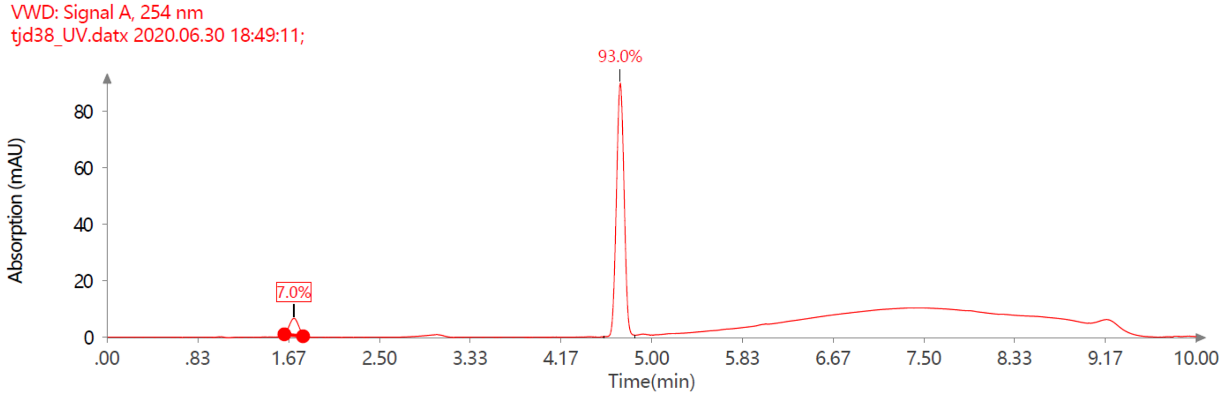

Time (Peak Maximum M:S/Minutes)	Maximum Intensity (c/s)	Time (Peak Centroid M:S/Minutes)	Peak Area	% Peak Area	Peak Resolution	Label
1.71	6E0	1.71	3.4E1	7.0	5.6	
4.71	9.1E1	4.71	4.6E2	93.0	4.7	

Tolyl 3-(4-((3-acetamidophenoxy)methyl)-1,2,3-triazol-1-yl)-3-deoxy-1-thio-β-D-galactopyranoside (48)

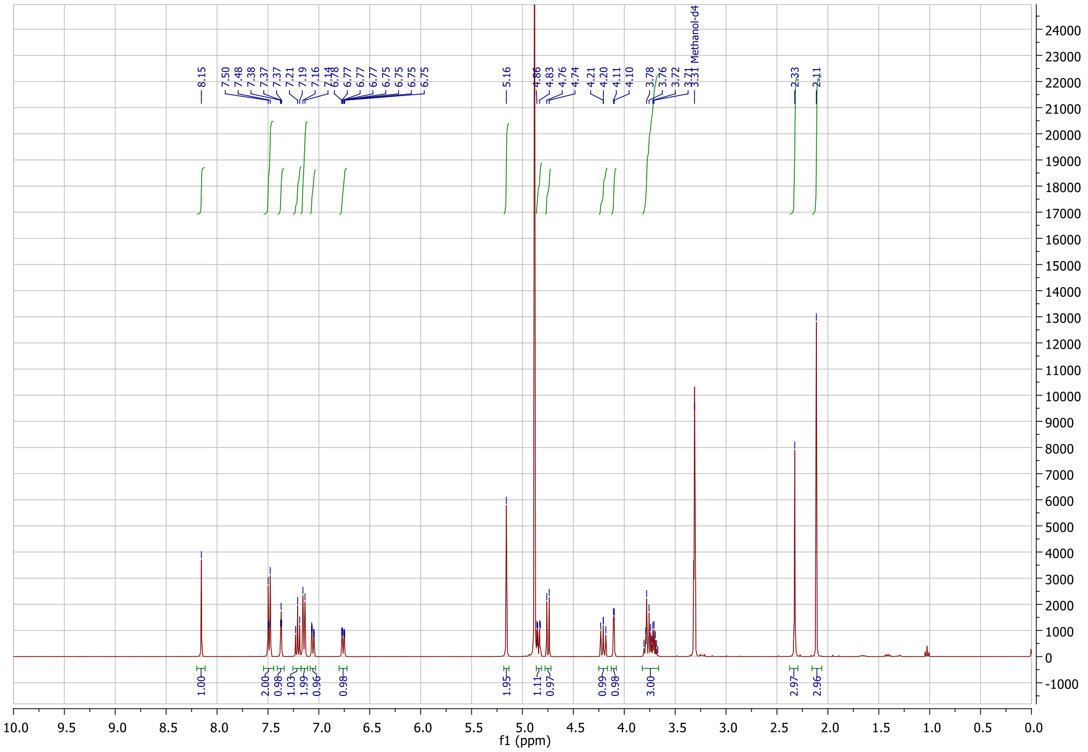

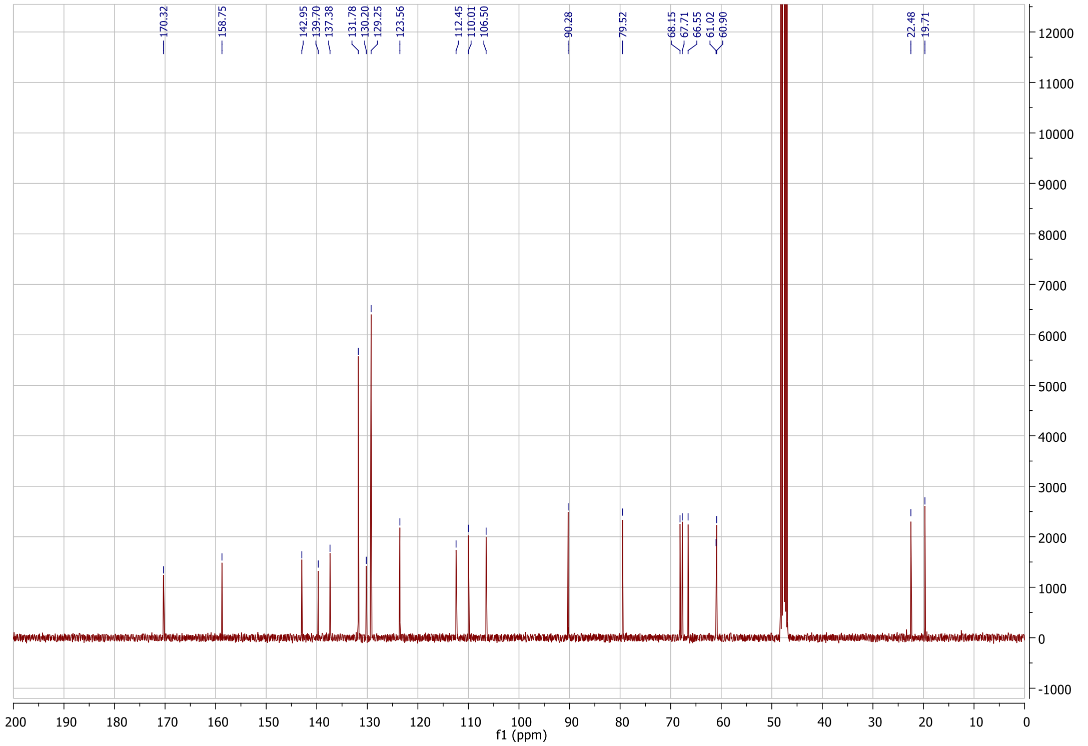

Chromatogram

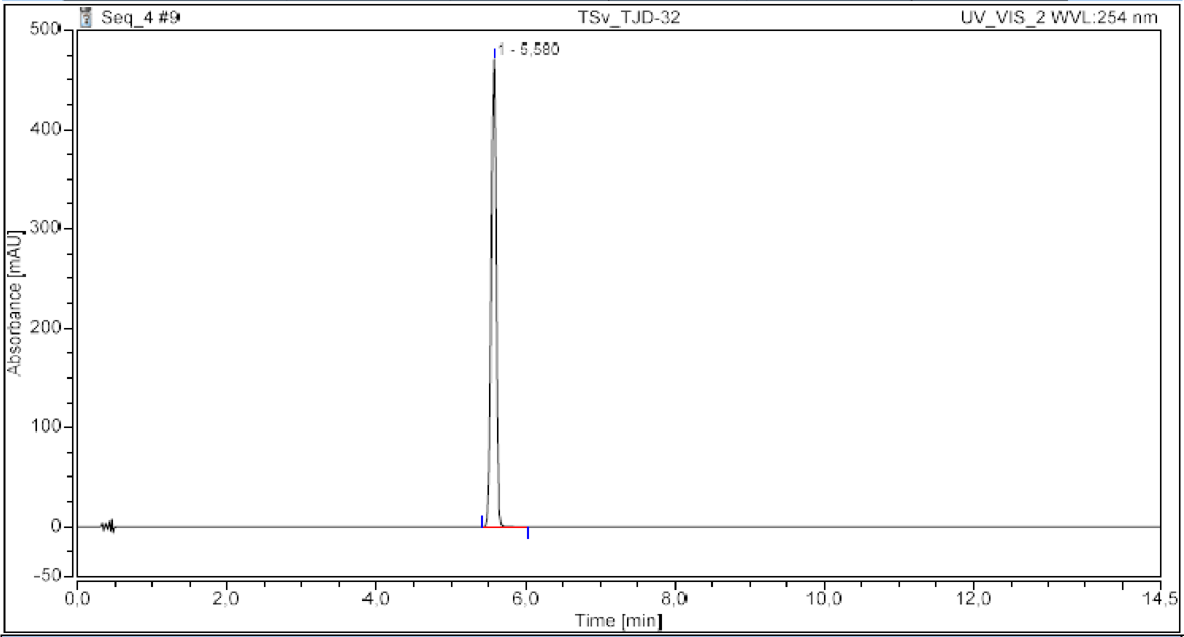

Integration Results

No.	Peak Name	Retention Time min	Area mAU*min	Height mAU	Relative Area %	Relative Height %	Amount n.a.
1		5,580	36,768	470,824	100,00	100,00	n.a.
**Total:**			**36,768**	**470,824**	**100,00**	**100,00**	

Tolyl 3-(4-((4-acetamidophenoxy)methyl)-1,2,3-triazol-1-yl)-3-deoxy-1-thio-β-D-galactopyranoside (49)

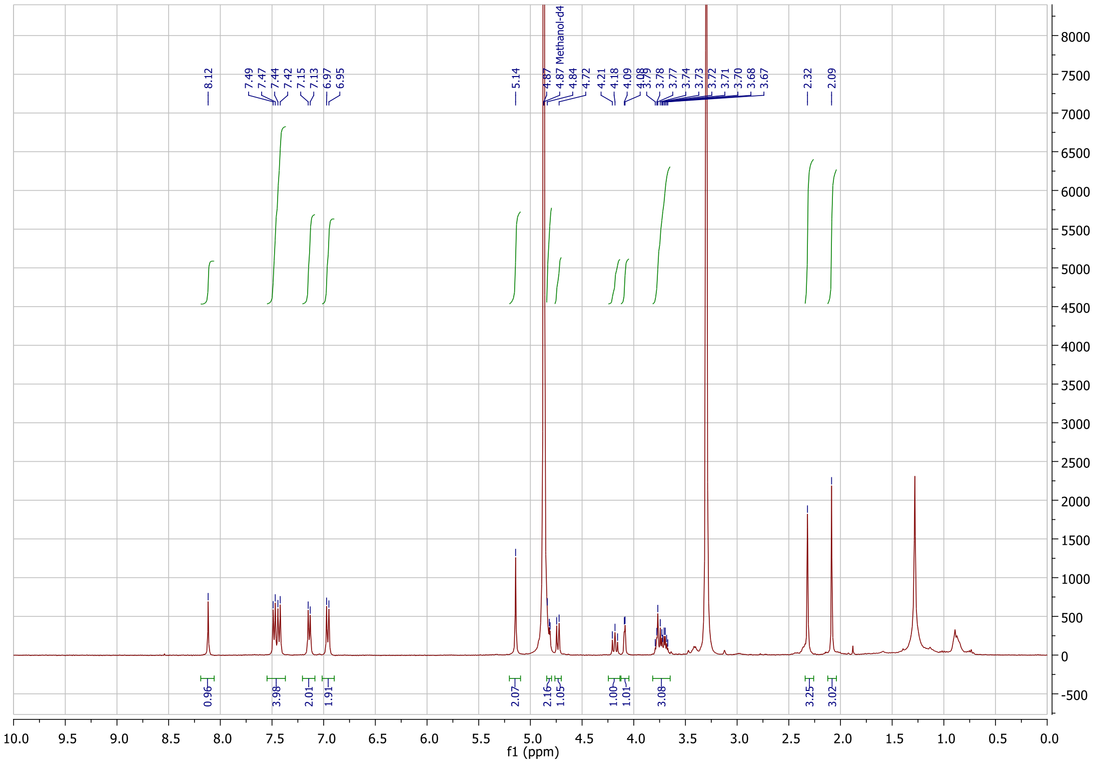

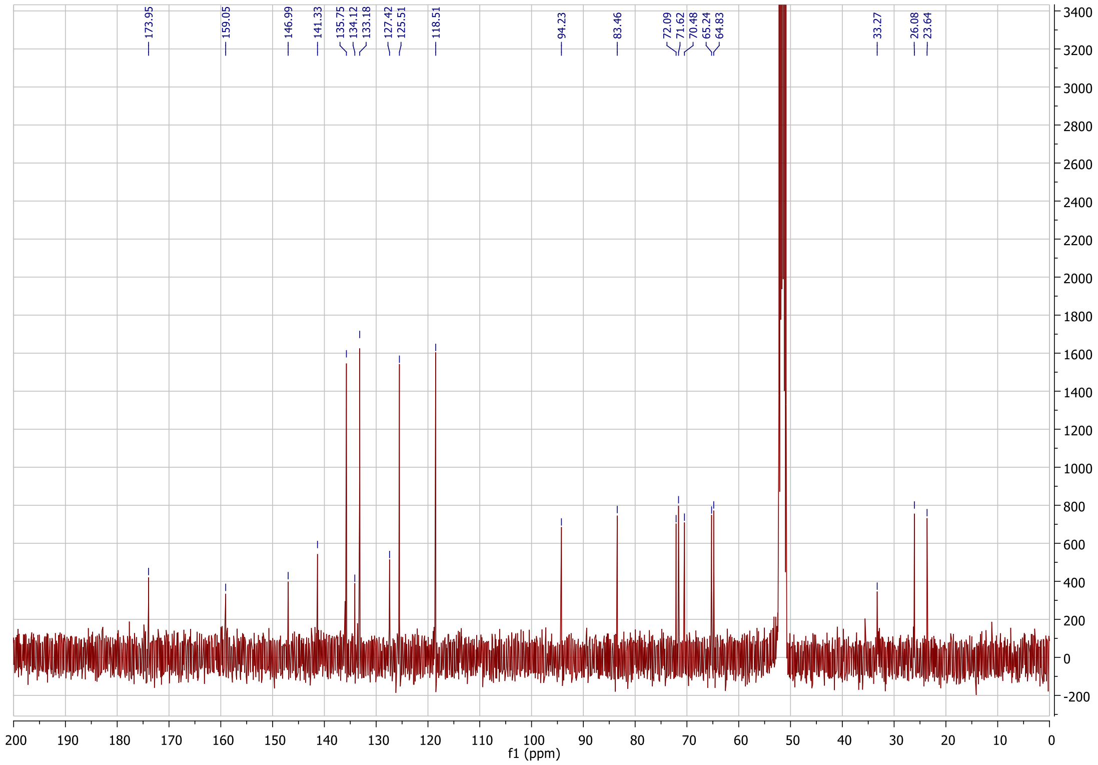

Chromatogram

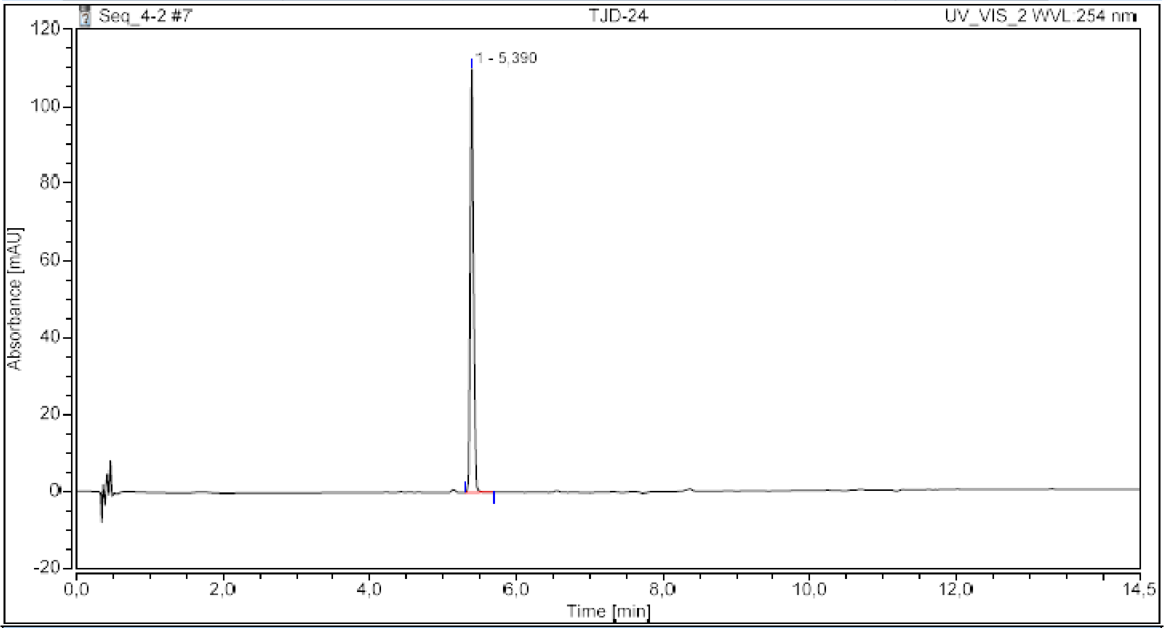

Integration Results

No.	Peak Name	Retention Time min	Area mAU*min	Height mAU	Relative Area %	Relative Height %	Amount n.a.
1		5,390	5,804	109,981	100,00	100,00	n.a.
**Total:**			**5,804**	**109,981**	**100,00**	**100,00**	

Tolyl 3-(4-((2-cyclopropanecarboxamidophenyloxy)-methyl)-1,2,3-triazol-1-yl)-3-deoxy-1-thio-β-D-galactopyranoside (50)

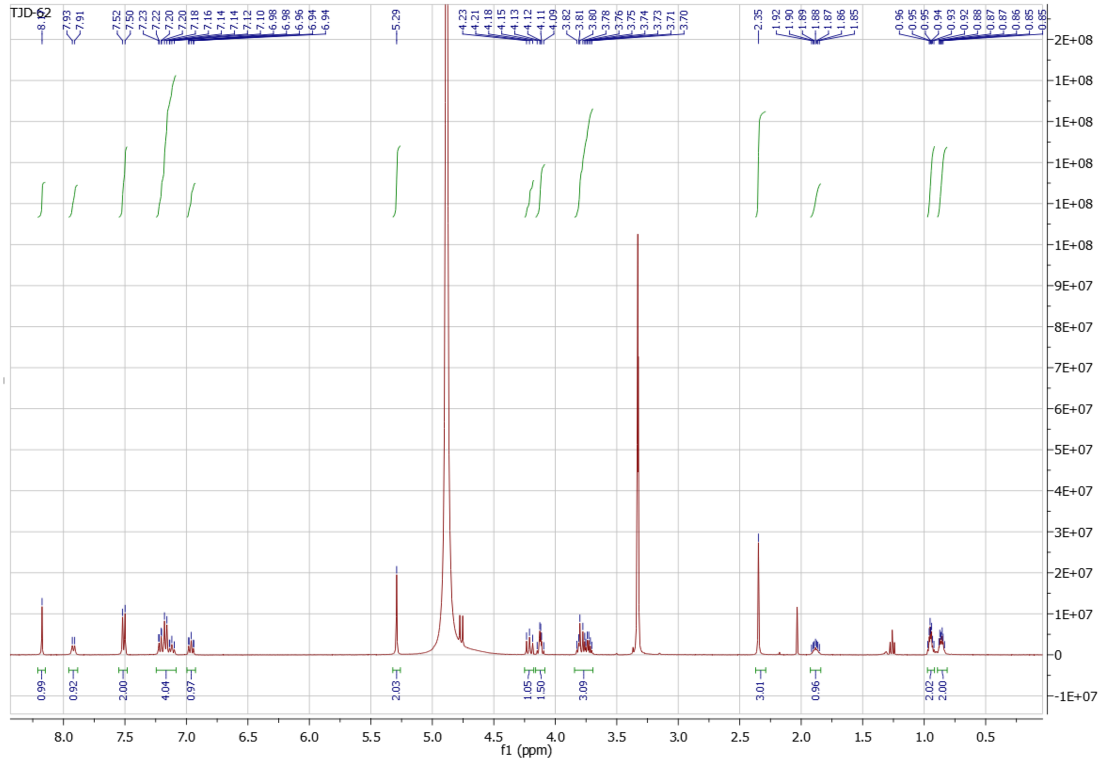

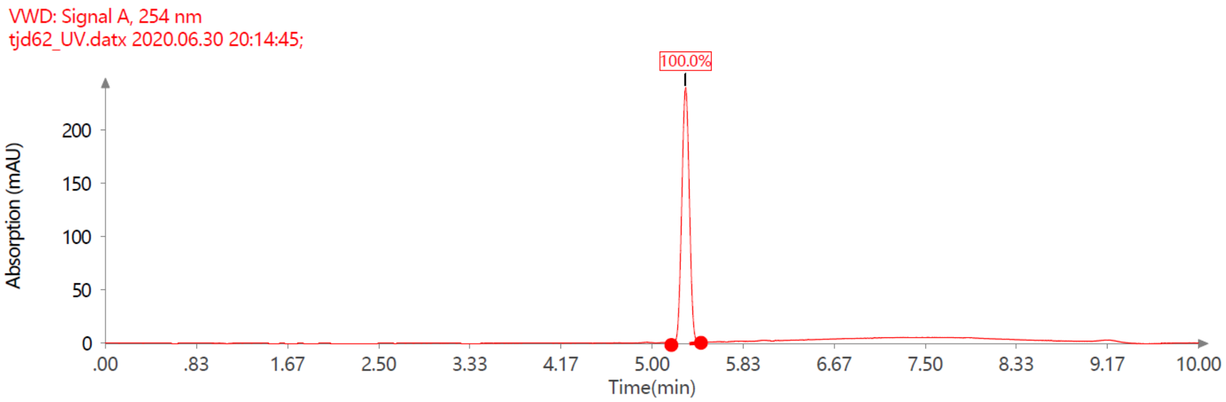

Time (Peak Maximum M:S/Minutes)	Maximum Intensity (c/s)	Time (Peak Centroid M:S/Minutes)	Peak Area	% Peak Area	Peak Resolution	Label
5.31	2.4E2	5.31	1.2E3	100.0	4.6	

Tolyl 3-(4-((2-(2-phenyl)acetamidophenyloxy)methyl)-1,2,3-triazol-1-yl)-3-deoxy-1-thio-β-D-galactopyranoside (51)

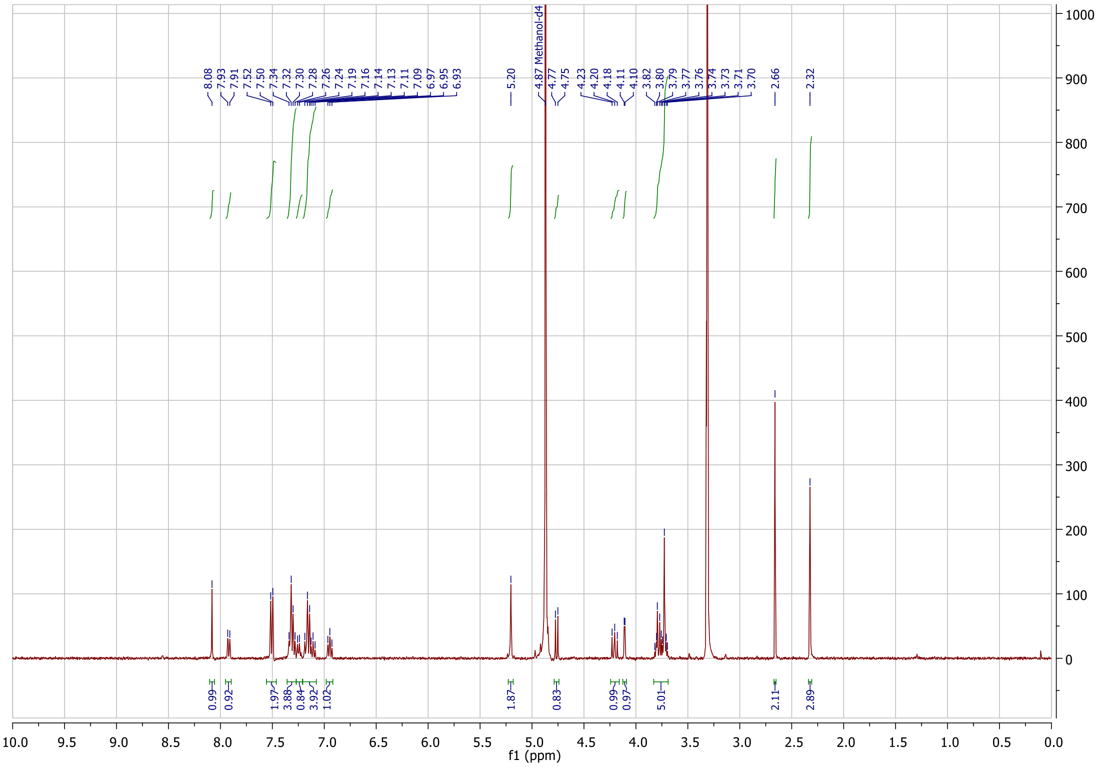

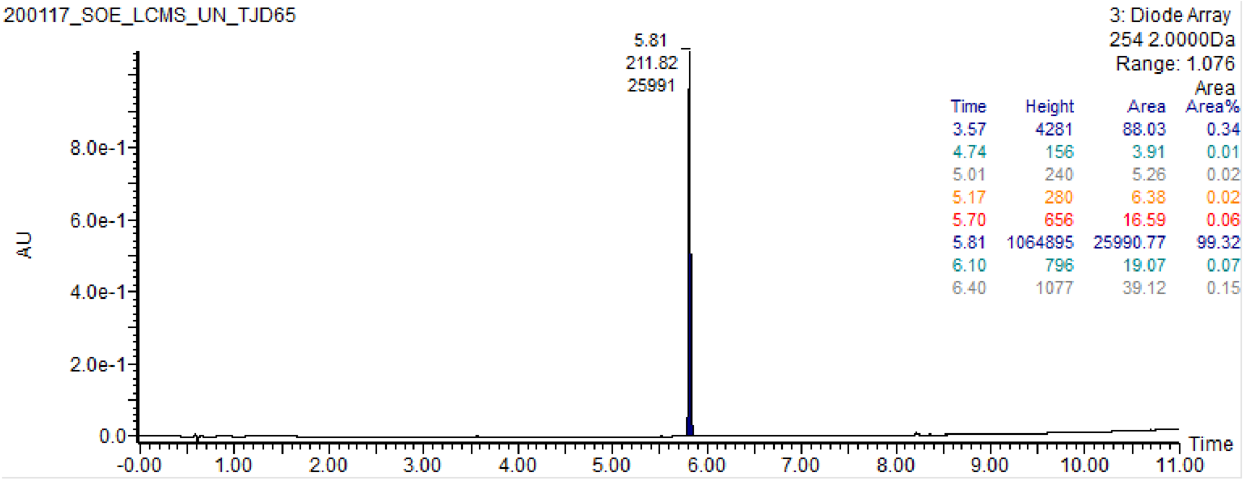

Tolyl 3-(4-((4-acetamido-2-nitrophenyloxy)methyl)-1,2,3-triazol-1-yl)-3-deoxy-1-thio-β-D-galactopyranoside (52)

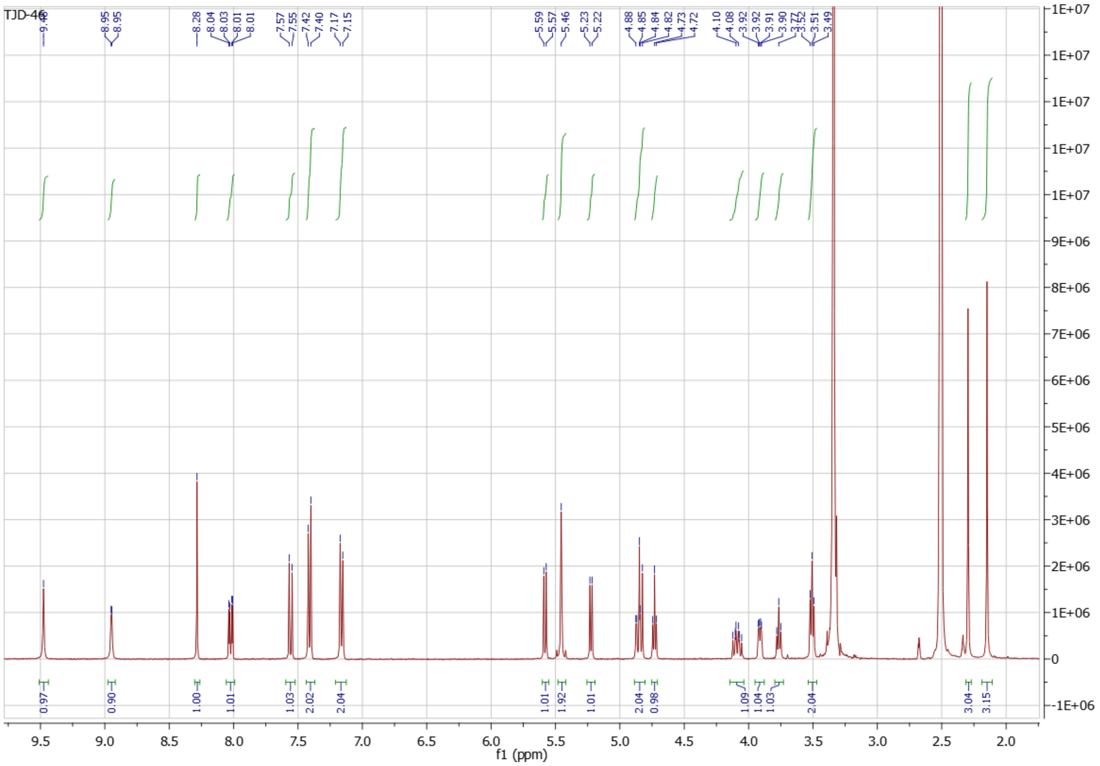

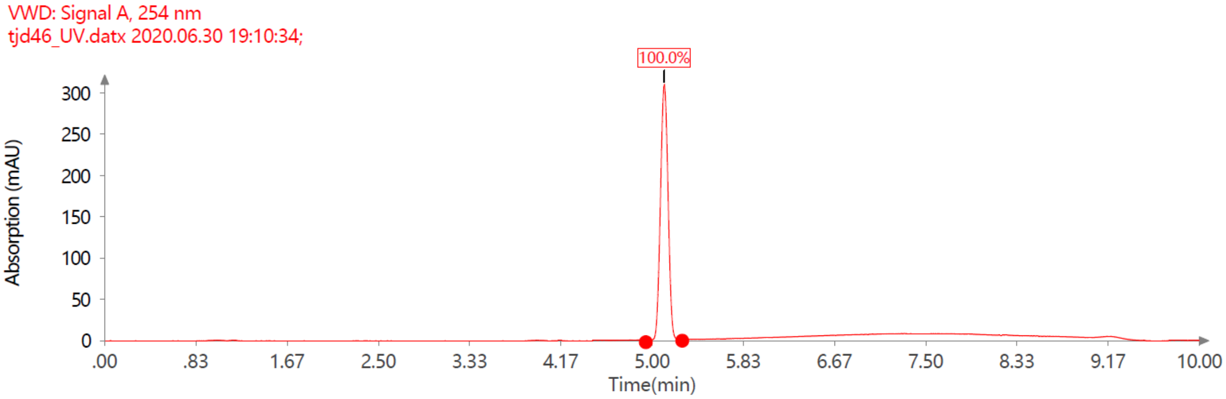

Time (Peak Maximum M:S/Minutes)	Maximum Intensity (c/s)	Time (Peak Centroid M:S/Minutes)	Peak Area	% Peak Area	Peak Resolution	Label
5.11	3.1E2	5.11	1.5E3	100.0	4.6	

Tolyl 3-(4-((2,5-diacetamidophenyloxy)methyl)-1,2,3-triazol-1-yl)-3-deoxy-1-thio-β-D-galactopyranoside (53)

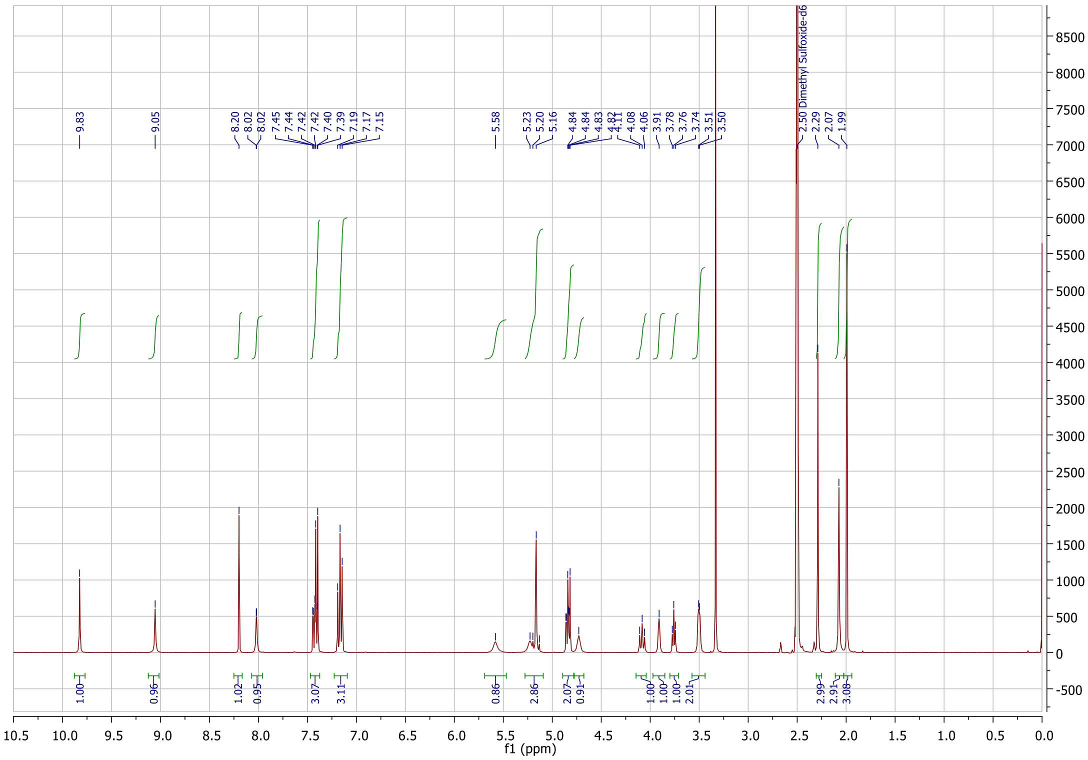

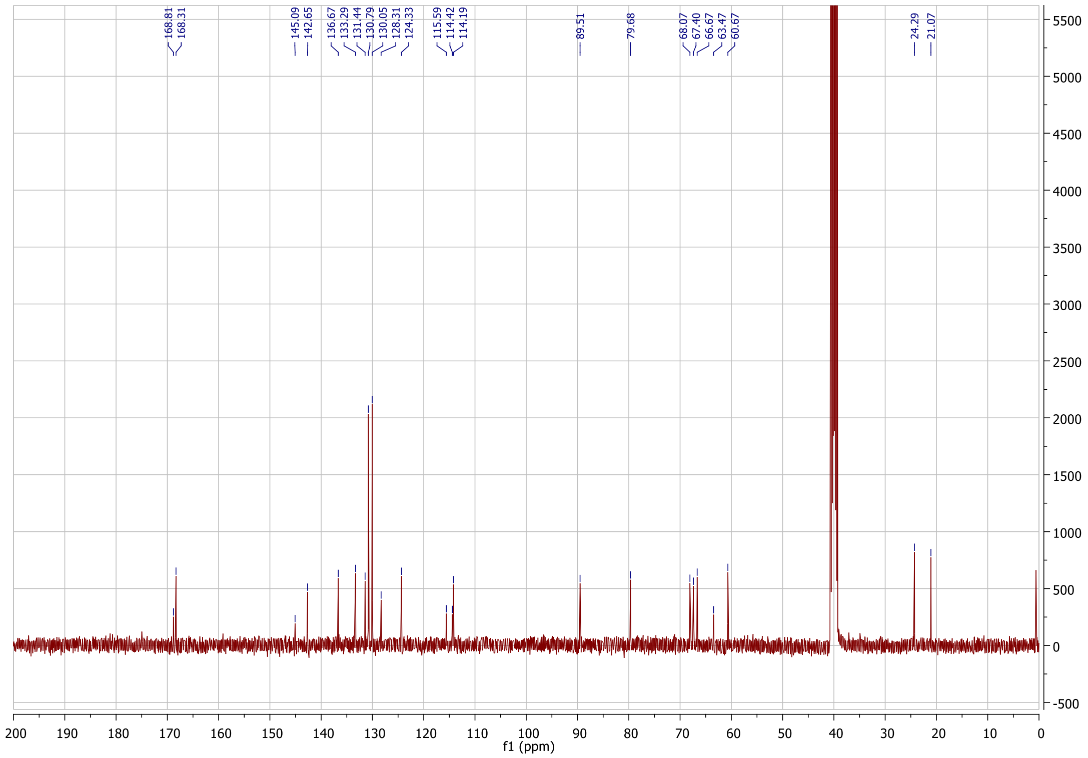

Chromatogram

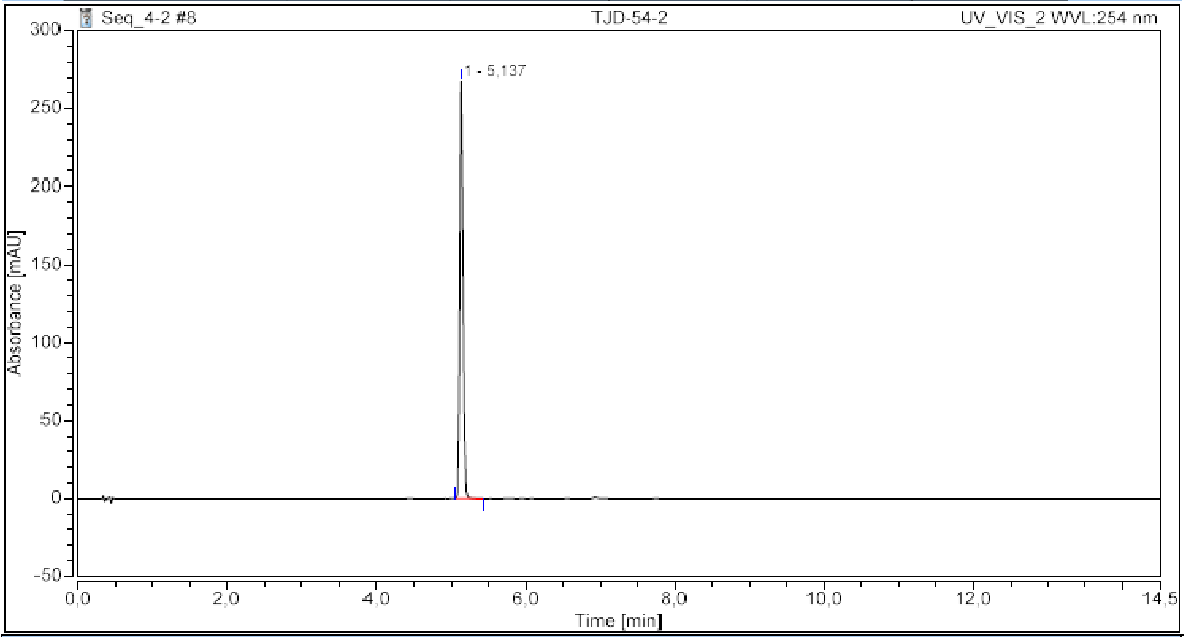

Integration Results

No.	Peak Name	Retention Time<br>min	Area<br>mAU*min	Height<br>mAU	Relative Area<br>%	Relative Height<br>%	Amount<br>n.a.
1		5,137	13,543	267,561	100,00	100,00	n.a.
**Total:**			**13,543**	**267,561**	**100,00**	**100,00**	

Tolyl 3-(4-((quinolin-8-oxy)methyl)-1,2,3-triazol-1-yl)-3-deoxy-1-thio-β-D-galactopyranoside (54)

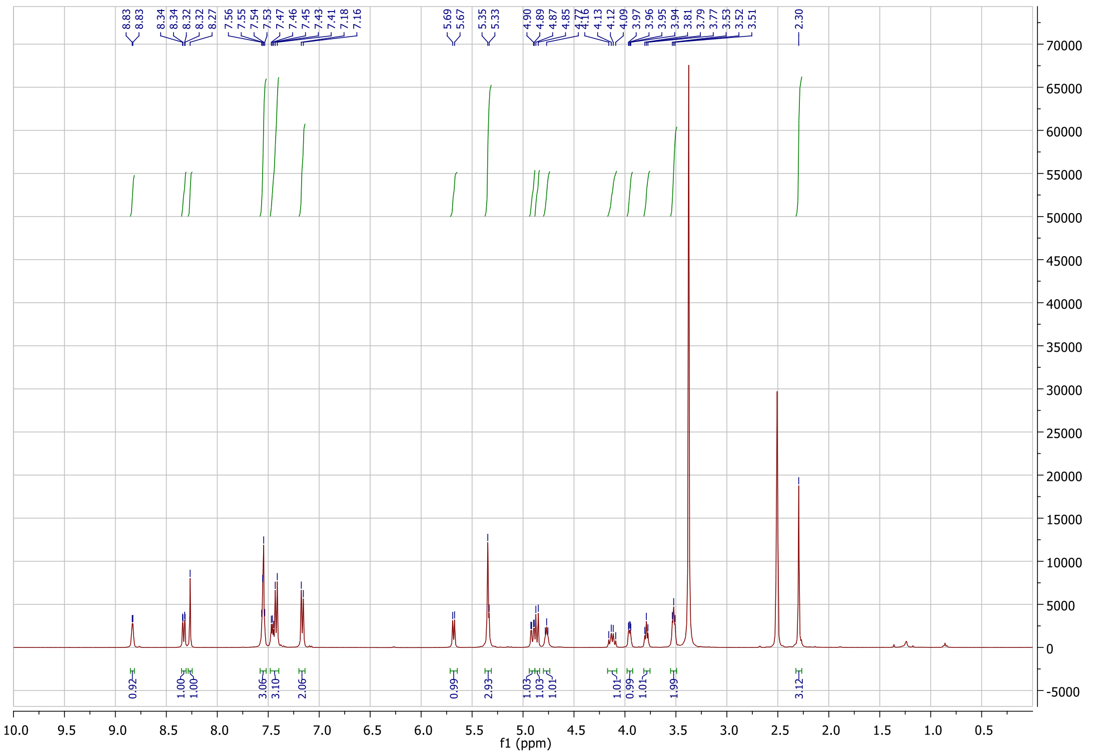

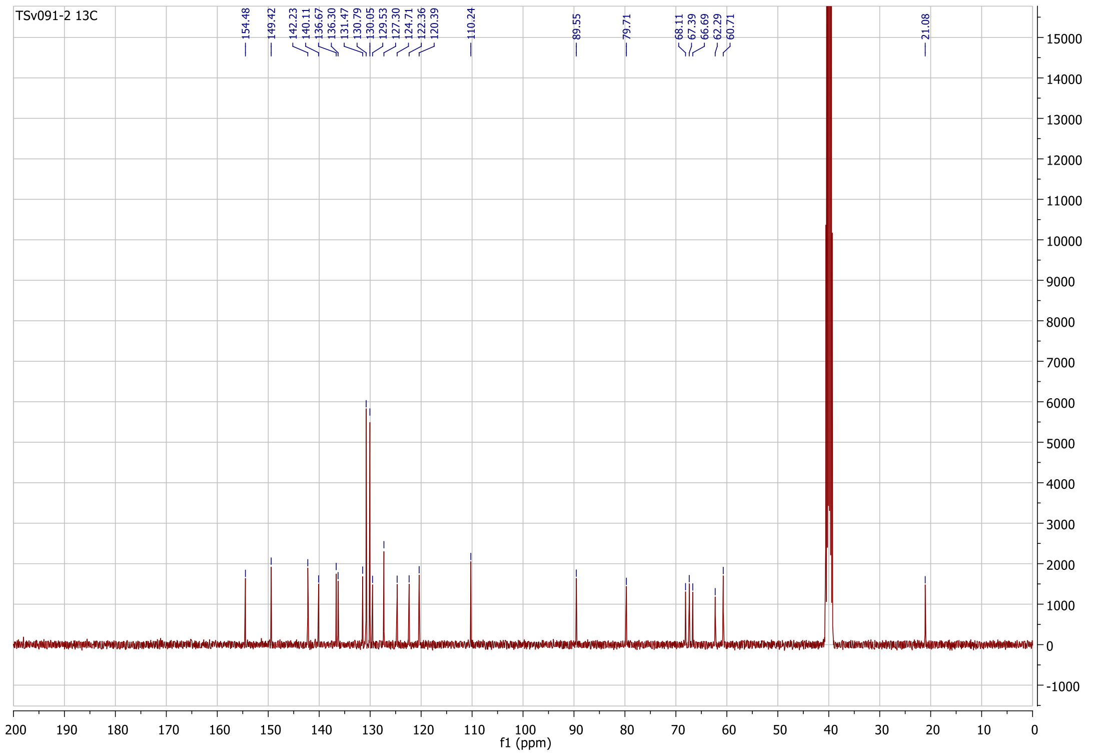

Chromatogram

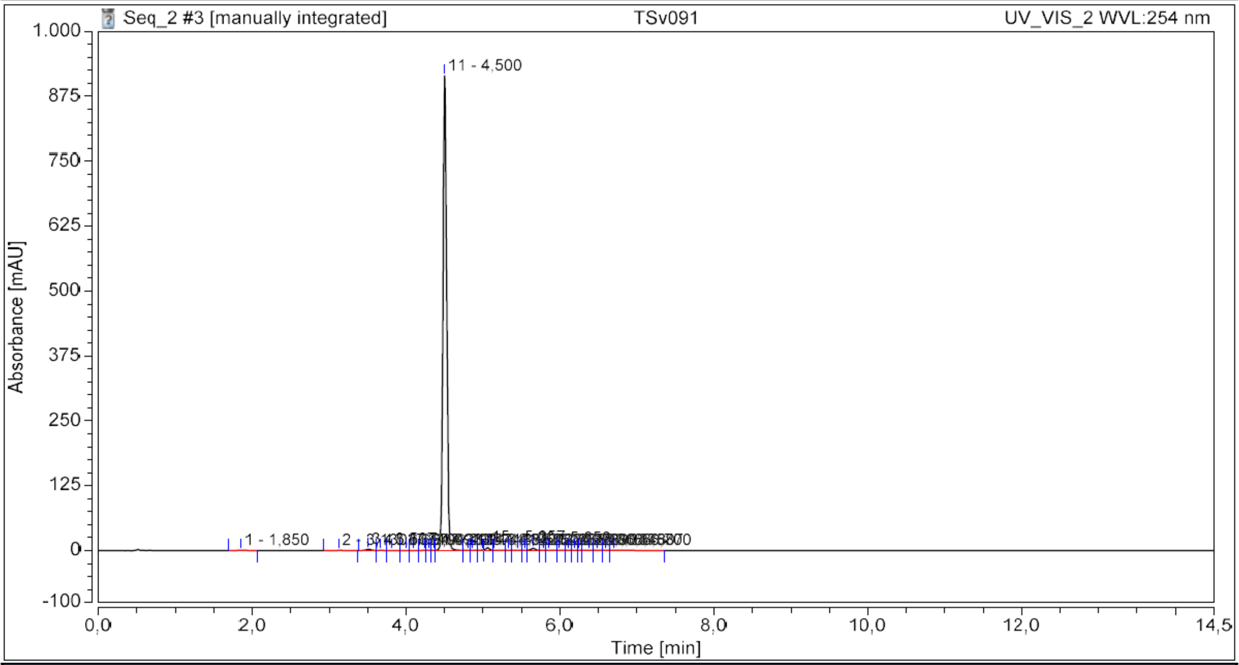

Integration Results

No.	Peak Name	Retention Time min	Area mAU*min	Height mAU	Relative Area %	Relative Height %	Amount n.a.
1		1,850	0,066	0,698	0,12	0,07	n.a.
2		3,130	0,038	0,292	0,07	0,03	n.a.
3		3,507	0,168	2,330	0,32	0,25	n.a.
4		3,667	0,026	0,344	0,05	0,04	n.a.
5		3,817	0,019	0,206	0,04	0,02	n.a.
6		3,993	0,013	0,169	0,02	0,02	n.a.
7		4,093	0,015	0,193	0,03	0,02	n.a.
8		4,233	0,014	0,272	0,03	0,03	n.a.
9		4,290	0,036	0,609	0,07	0,06	n.a.
10		4,357	0,029	0,585	0,06	0,06	n.a.
11		4,500	50,980	914,709	96,80	97,47	n.a.
12		4,810	0,055	0,634	0,10	0,07	n.a.
13		4,857	0,047	0,642	0,09	0,07	n.a.
14		4,987	0,039	0,689	0,07	0,07	n.a.
15		5,057	0,286	5,763	0,54	0,61	n.a.
16		5,143	0,074	0,534	0,14	0,06	n.a.
17		5,323	0,027	0,383	0,05	0,04	n.a.
18		5,453	0,051	0,457	0,10	0,05	n.a.
19		5,537	0,030	0,506	0,06	0,05	n.a.
20		5,650	0,284	4,582	0,54	0,49	n.a.
21		5,780	0,042	0,534	0,08	0,06	n.a.
22		5,853	0,054	0,490	0,10	0,05	n.a.
23		5,993	0,038	0,387	0,07	0,04	n.a.
24		6,113	0,028	0,354	0,05	0,04	n.a.
25		6,187	0,026	0,403	0,05	0,04	n.a.
26		6,247	0,017	0,275	0,03	0,03	n.a.
27		6,373	0,047	0,469	0,09	0,05	n.a.
28		6,480	0,046	0,548	0,09	0,06	n.a.
29		6,587	0,017	0,204	0,03	0,02	n.a.
30		6,700	0,053	0,173	0,10	0,02	n.a.
**Total:**			**52,662**	**938,435**	**100,00**	**100,00**	

Tolyl 3-(4-((2-methoxycarbonylquinolin-8-oxy)methyl)-1,2,3-triazol-1-yl)-3-deoxy-1-thio-β-D-galactopyranoside (55)

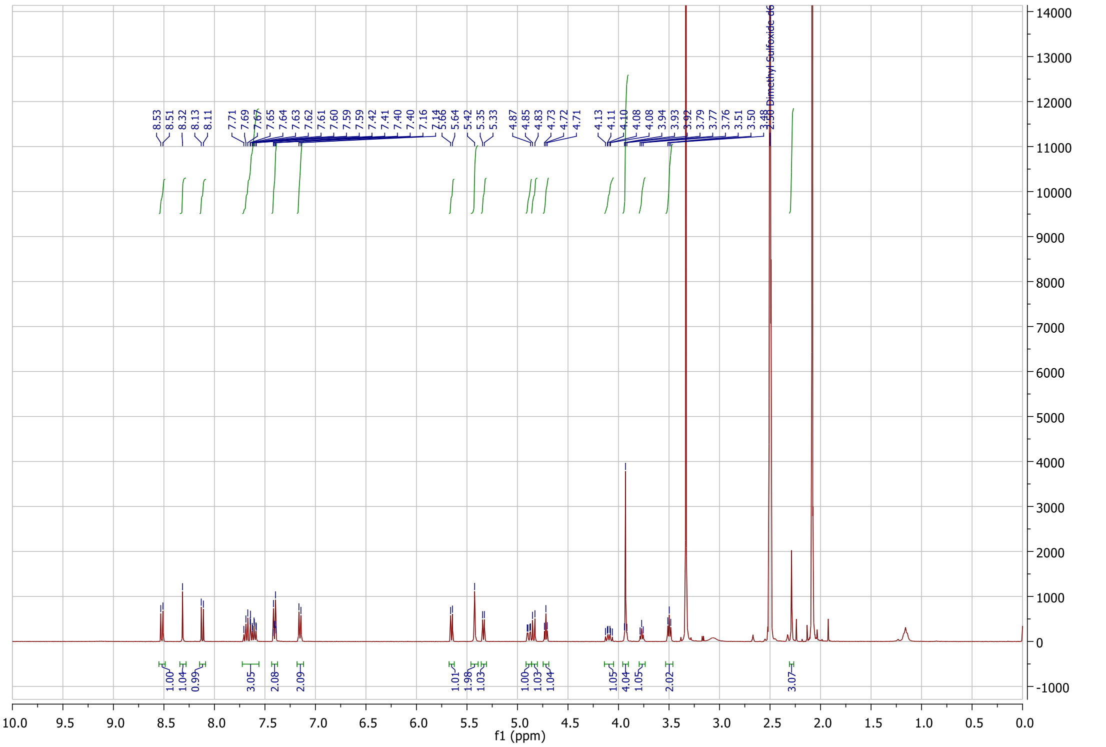

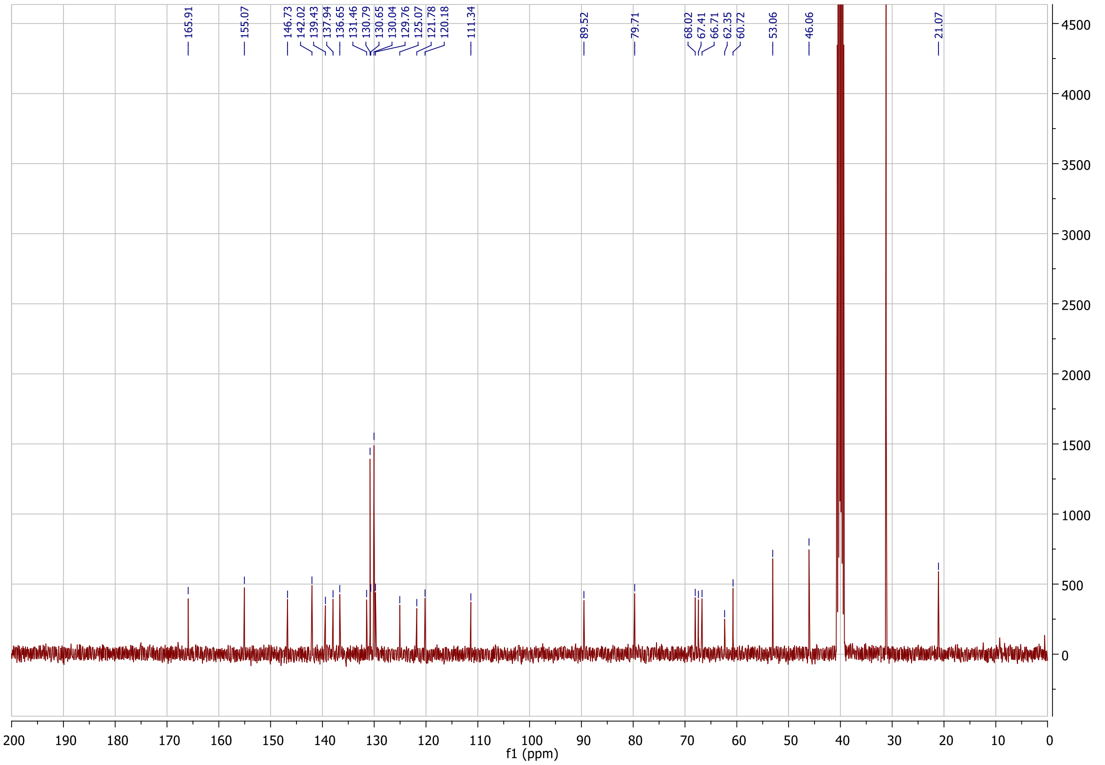

Chromatogram

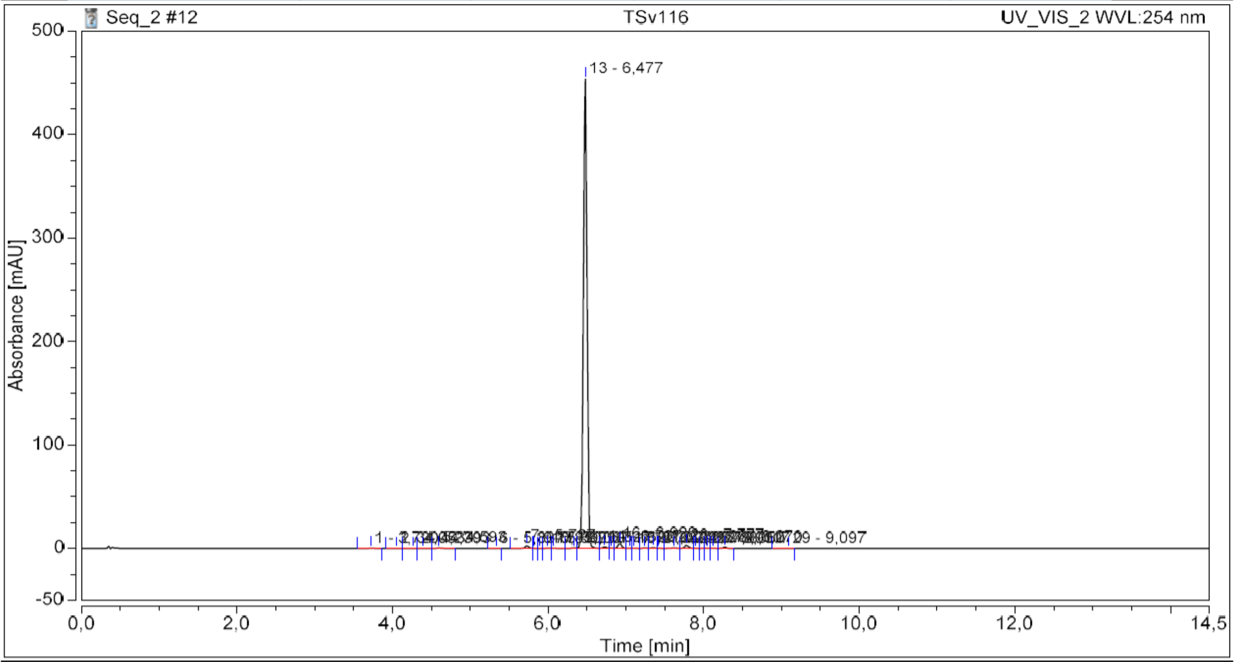

Integration Results

No.	Peak Name	Retention Time min	Area mAU*min	Height mAU	Relative Area %	Relative Height %	Amount n.a.
1		3,720	0,017	0,218	0,07	0,05	n.a.
2		4,053	0,006	0,062	0,02	0,01	n.a.
3		4,270	0,007	0,083	0,03	0,02	n.a.
4		4,393	0,011	0,097	0,04	0,02	n.a.
5		4,593	0,038	0,540	0,15	0,11	n.a.
6		5,337	0,003	0,041	0,01	0,01	n.a.
7		5,727	0,151	2,824	0,59	0,60	n.a.
8		5,810	0,005	0,138	0,02	0,03	n.a.
9		5,897	0,003	0,048	0,01	0,01	n.a.
10		5,990	0,022	0,342	0,08	0,07	n.a.
11		6,067	0,012	0,126	0,05	0,03	n.a.
12		6,327	0,031	0,516	0,12	0,11	n.a.
13		6,477	24,565	453,676	95,56	95,96	n.a.
14		6,727	0,086	1,365	0,33	0,29	n.a.
15		6,810	0,031	0,475	0,12	0,10	n.a.
16		6,920	0,282	5,024	1,10	1,06	n.a.
17		7,053	0,013	0,224	0,05	0,05	n.a.
18		7,097	0,016	0,246	0,06	0,05	n.a.
19		7,240	0,033	0,542	0,13	0,11	n.a.
20		7,350	0,042	0,645	0,16	0,14	n.a.
21		7,413	0,011	0,230	0,04	0,05	n.a.
22		7,617	0,037	0,473	0,14	0,10	n.a.
23		7,777	0,184	3,097	0,72	0,65	n.a.
24		7,893	0,008	0,154	0,03	0,03	n.a.
25		8,010	0,006	0,111	0,02	0,02	n.a.
26		8,050	0,010	0,169	0,04	0,04	n.a.
27		8,117	0,012	0,176	0,05	0,04	n.a.
28		8,270	0,062	1,108	0,24	0,23	n.a.
29		9,097	0,004	0,033	0,02	0,01	n.a.
**Total:**			**25,707**	**472,783**	**100,00**	**100,00**	

Tolyl 3-(4-((2-carboxyquinolin-8-oxy)methyl)-1,2,3-triazol-1-yl)-3-deoxy-1-thio-β-D-galactopyranoside (56)

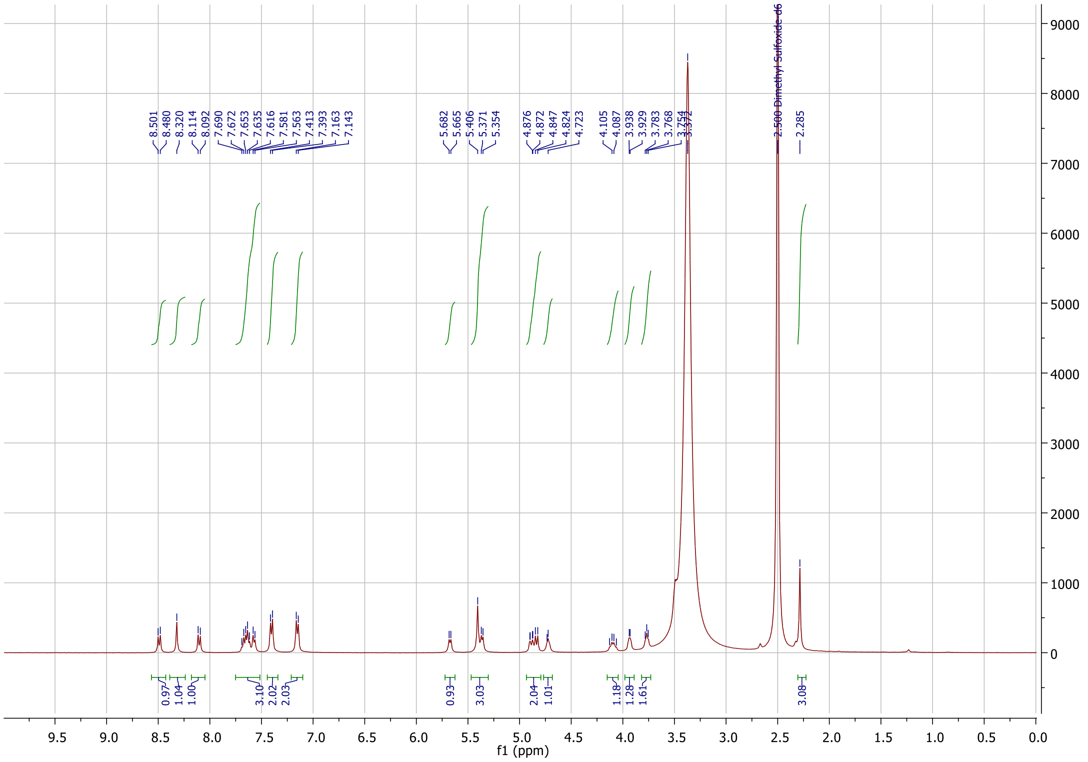

Chromatogram

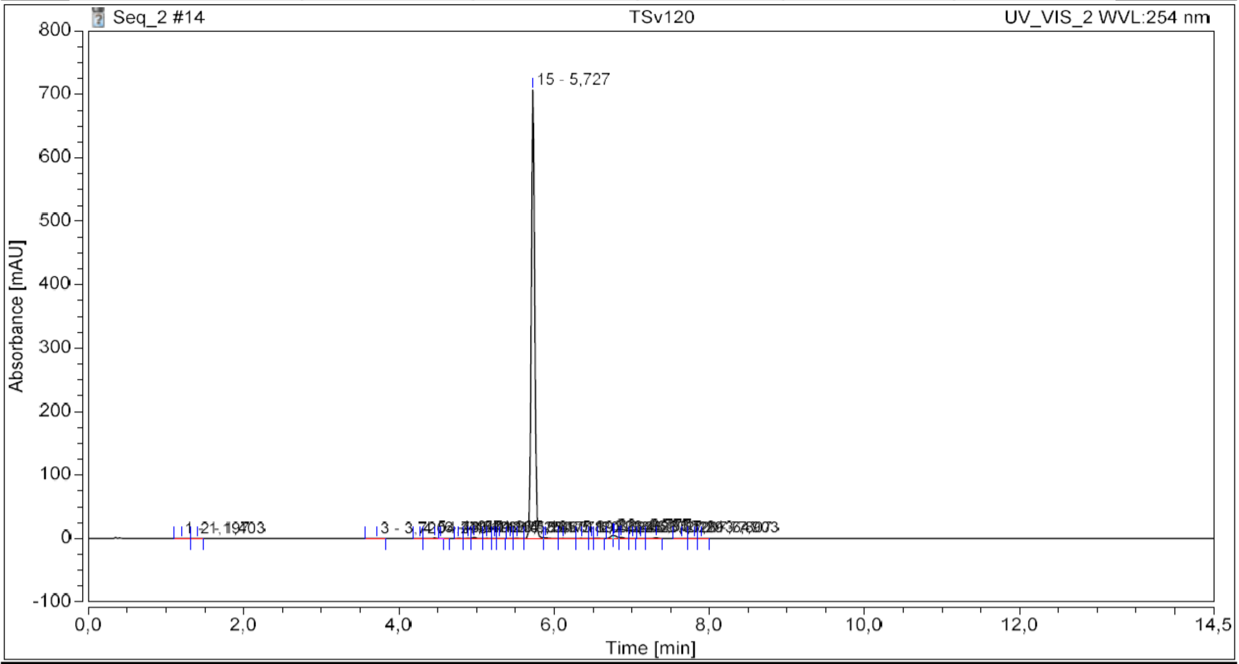

Integration Results

No.	Peak Name	Retention Time min	Area mAU*min	Height mAU	Relative Area %	Relative Height %	Amount n.a.
1		1,197	0,005	0,061	0,01	0,01	n.a.
2		1,403	0,003	0,042	0,01	0,01	n.a.
3		3,720	0,006	0,066	0,02	0,01	n.a.
4		4,270	0,003	0,043	0,01	0,01	n.a.
5		4,457	0,059	0,993	0,16	0,14	n.a.
6		4,540	0,001	0,030	0,00	0,00	n.a.
7		4,763	0,022	0,523	0,06	0,07	n.a.
8		4,887	0,026	0,574	0,07	0,08	n.a.
9		4,967	0,097	2,006	0,26	0,28	n.a.
10		5,133	0,018	0,337	0,05	0,05	n.a.
11		5,230	0,005	0,088	0,01	0,01	n.a.
12		5,297	0,007	0,110	0,02	0,02	n.a.
13		5,430	0,011	0,169	0,03	0,02	n.a.
14		5,517	0,038	0,488	0,10	0,07	n.a.
15		5,727	35,974	706,838	97,26	97,30	n.a.
16		5,893	0,073	0,949	0,20	0,13	n.a.
17		6,110	0,025	0,240	0,07	0,03	n.a.
18		6,350	0,015	0,208	0,04	0,03	n.a.
19		6,483	0,007	0,135	0,02	0,02	n.a.
20		6,553	0,018	0,261	0,05	0,04	n.a.
21		6,753	0,175	4,513	0,47	0,62	n.a.
22		6,777	0,216	4,565	0,58	0,63	n.a.
23		6,863	0,101	1,924	0,27	0,26	n.a.
24		7,010	0,002	0,037	0,01	0,01	n.a.
25		7,120	0,006	0,075	0,02	0,01	n.a.
26		7,313	0,057	0,958	0,15	0,13	n.a.
27		7,647	0,009	0,103	0,02	0,01	n.a.
28		7,807	0,004	0,061	0,01	0,01	n.a.
29		7,903	0,006	0,076	0,02	0,01	n.a.
**Total:**			**36,990**	**726,473**	**100,00**	**100,00**	

3,4-Dichlorophenyl 2,4,6-tri-O-acetyl-3-(4-((2-acetamidophenoxy)methyl)-1,2,3-triazol-1-yl)-3-deoxy-1-thio-α-D-galactopyranoside (58)

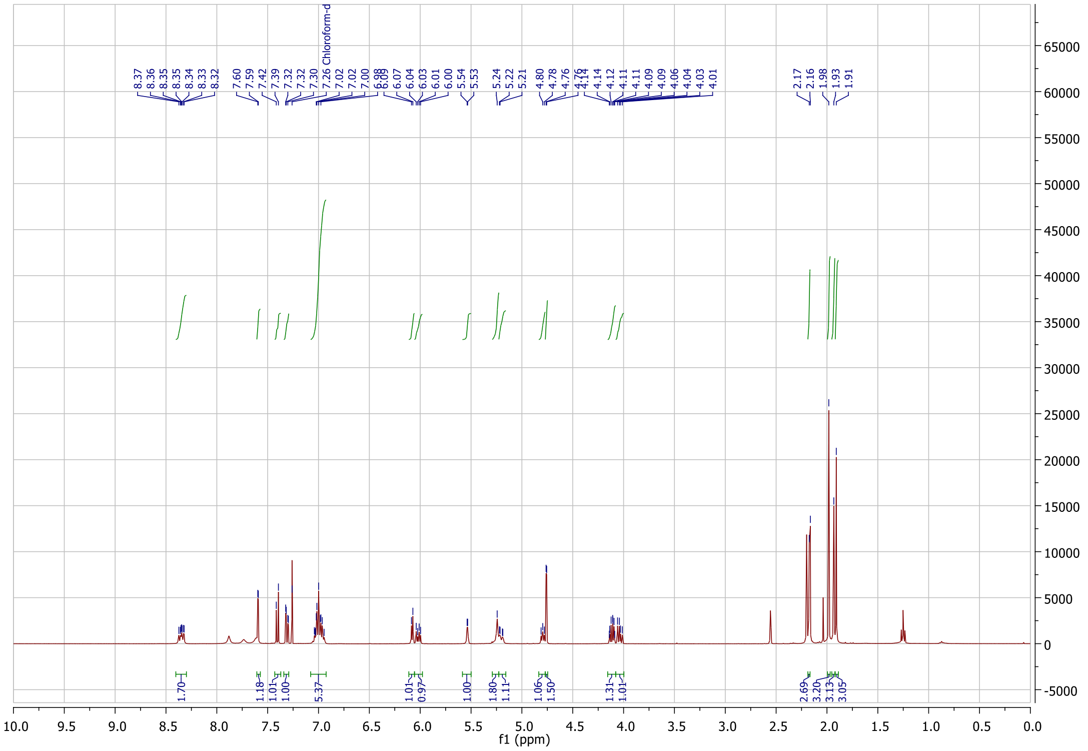

3,4-Dichlorophenyl 3-(4-((2-acetamidophenoxy)methyl)-1,2,3-triazol-1-yl)-3-deoxy-1-thio-α-D-galactopyranoside (59)

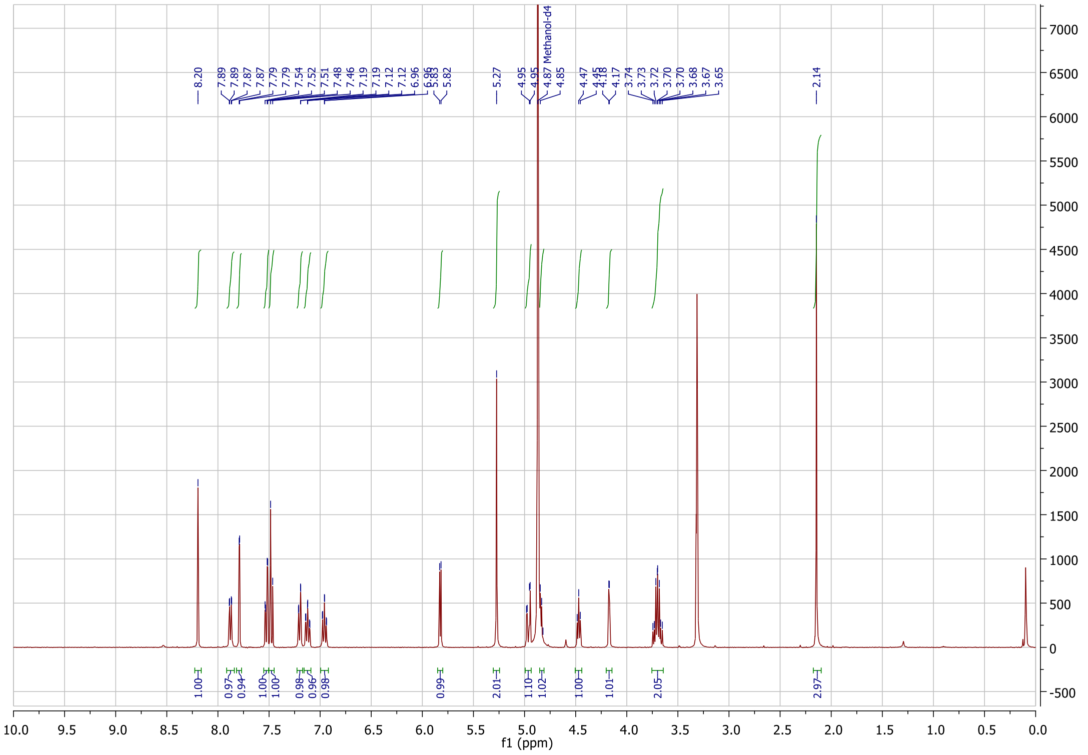

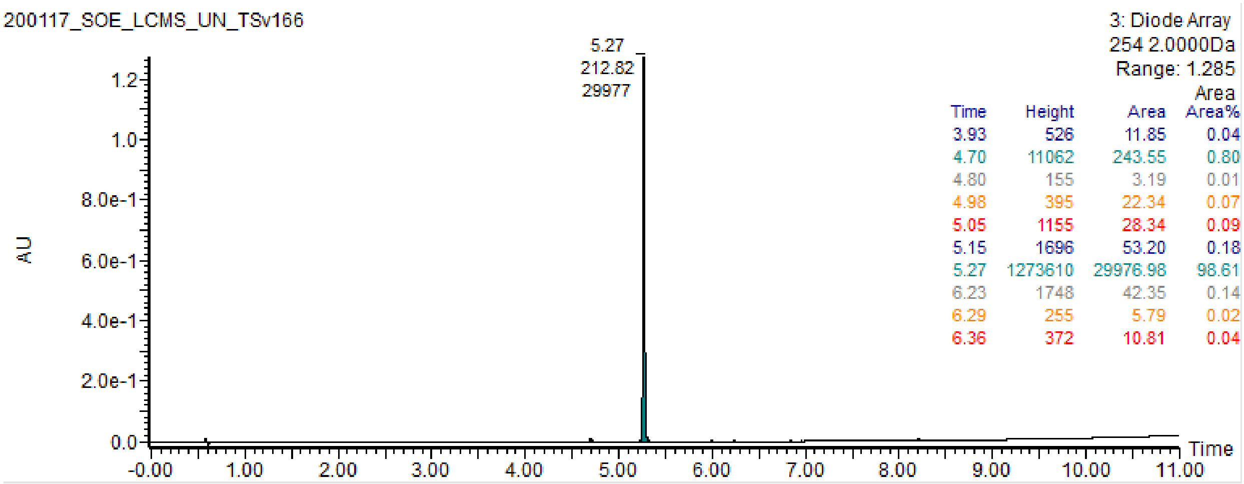

Di-(2,4,6-tri-O-acetyl-3-(4-((2-acetamidophenyloxy)methyl)-1,2,3-triazol-1-yl)-3-deoxy-β-D-galactopyranosyl) sulfane (61)

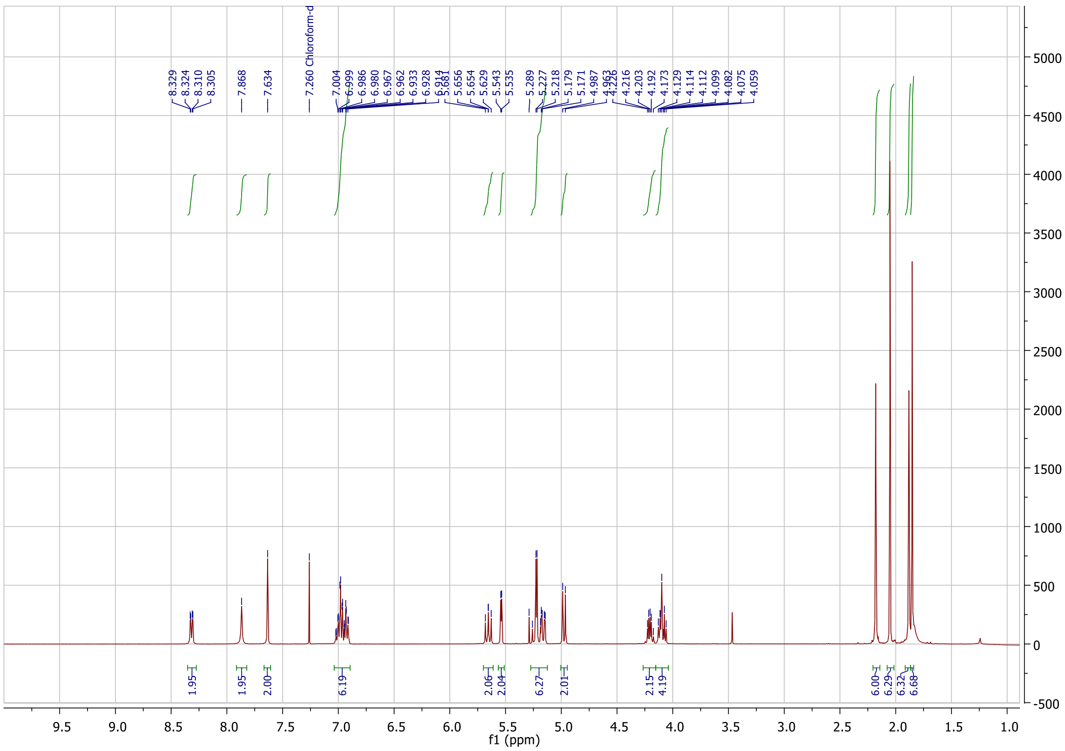

Di-(3-(4-((2-acetamidophenyloxy)methyl)-1,2,3-triazol-1-yl)-3-deoxy-β-D-galactopyranosyl) sulfane (62)

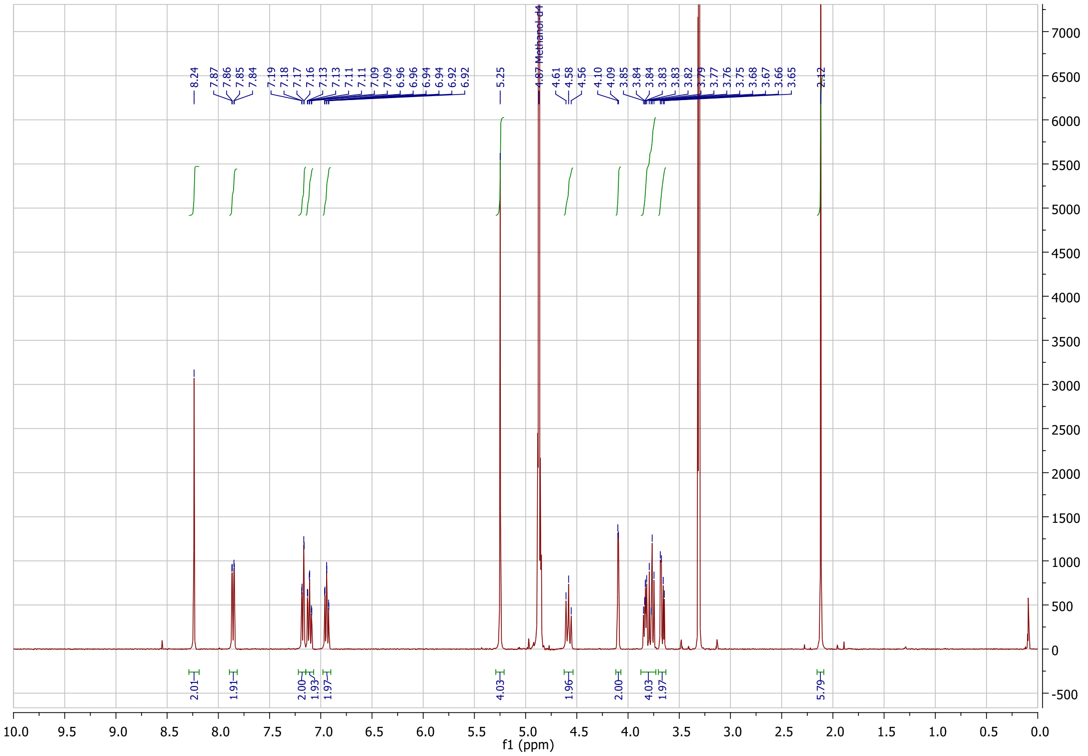

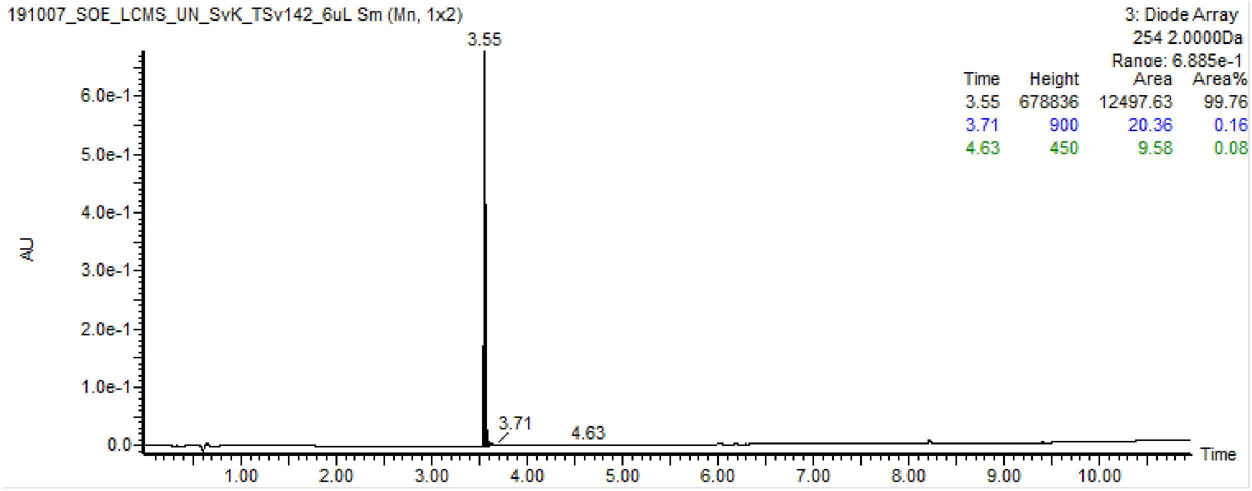

Supplement: RA-012-D2RA03163A-s001 [file RA-012-D2RA03163A-s001.pdf]
